# Revisiting activity of some glucocorticoids as a potential inhibitor of SARS-CoV-2 main protease: theoretical study†

**DOI:** 10.1039/d0ra10674g

**Published:** 2021-03-09

**Authors:** Ayman Abo Elmaaty, Radwan Alnajjar, Mohammed I. A. Hamed, Muhammad Khattab, Mohamed M. Khalifa, Ahmed A. Al-Karmalawy

**Affiliations:** Department of Medicinal Chemistry, Faculty of Pharmacy, Port Said University Port Said 42526 Egypt; Department of Chemistry, Faculty of Science, University of Benghazi Benghazi Libya; Department of Chemistry, University of Cape Town Rondebosch 7701 South Africa; Department of Organic and Medicinal Chemistry, Faculty of Pharmacy, Fayoum University Fayoum 63514 Egypt; Department of Chemistry of Natural and Microbial Products, Division of Pharmaceutical and Drug Industries, National Research Centre Cairo 12622 Egypt; Department of Pharmaceutical Medicinal Chemistry& Drug Design, Faculty of Pharmacy (Boys), Al-Azhar University Cairo 11884 Egypt; Department of Pharmaceutical Medicinal Chemistry, Faculty of Pharmacy, Horus University-Egypt New Damietta 34518 Egypt akarmalawy@horus.edu.eg

## Abstract

The global breakout of COVID-19 and raised death toll has prompted scientists to develop novel drugs capable of inhibiting SARS-CoV-2. Conducting studies on repurposing some FDA-approved glucocorticoids can be a promising prospective for finding a treatment for COVID-19. In addition, the use of anti-inflammatory drugs, such as glucocorticoids, is a pivotal step in the treatment of critical cases of COVID-19, as they can provoke an inflammatory cytokine storm, damaging lungs. In this study, 22 FDA-approved glucocorticoids were identified through *in silico* (molecular docking) studies as the potential inhibitors of COVID-19's main protease. From tested compounds, ciclesonide 11, dexamethasone 2, betamethasone 1, hydrocortisone 4, fludrocortisone 3, and triamcinolone 8 are suggested as the most potent glucocorticoids active against COVID-19's main protease. Moreover, molecular dynamics simulations followed by the calculations of the binding free energy using MM-GBSA were carried out for the aforementioned promising candidate-screened glucocorticoids. In addition, quantum chemical calculations revealed two electron-rich sites on ciclesonide where binding interactions with the main protease and cleavage of the prodrug to the active metabolite take place. Our results have ramifications for conducting preclinical and clinical studies on promising glucocorticoids to hasten the development of effective therapeutics against COVID-19. Another advantage is that some glucocorticoids can be prioritized over others for the treatment of inflammation accompanying COVID-19.

## Abstract 2

The global breakout of COVID-19 and raised death toll has prompted scientists to develop novel drugs capable of inhibiting SARS-CoV-2.

## Graphical abstract

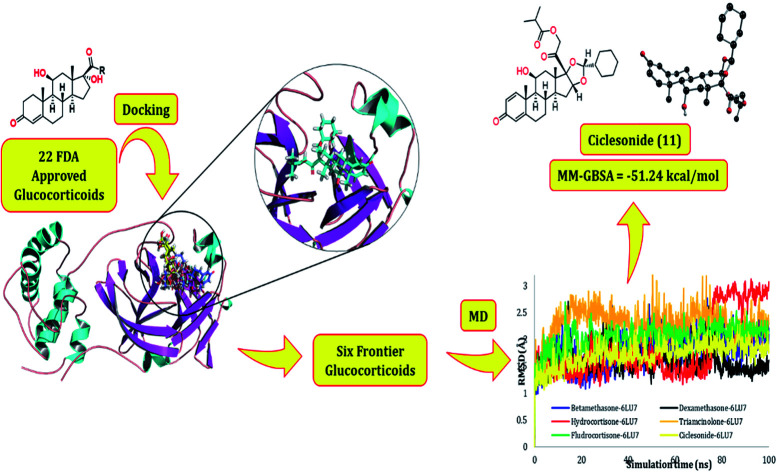

## 1. Introduction

In December 2019, a sudden outbreak of a new virus, severe acute respiratory syndrome coronavirus 2 (SARS-CoV-2), occurred in the Chinese city of Wuhan, causing coronavirus disease 2019 (COVID-19).^1^ The virus began to spread outside China all over the world within a short time. On March 11, 2020, after the overwhelming global expansion of SARS-CoV-2, the World Health Organization classified COVID-19 as a pandemic.^2^ The number of confirmed COVID-19 cases on December 12, 2020, was approximately 71 797 890 patients with a death toll up to 1 607 590 in over about 216 countries.^3^ It is a pity that SARS-CoV-2 is highly infectious and might develop some fatal consequences. Some patients diagnosed with COVID-19 may develop septic shock, respiratory failure, and multi-organ dysfunction, leading to a global mortality rate of about 4%.^4^

Besides, one of the most common features that critical COVID-19 patients may develop is extremely high inflammatory parameters, including C reactive protein and pro-inflammatory cytokines.^4,5^ Unfortunately, developing a new drug or vaccine that can cure COVID-19 effectively is a big global challenge.

The development of a new drug or vaccine is a long process that requires a lot of time, effort, and money. It may take years starting from the *de novo* design of drug candidates to the drug being clinically approved and available in markets. Hence this is one of the critical challenges that pharmaceutical industries may experience lately. So, we should shed light on alternative approaches that may allow intellectual intervention, helping our fight against COVID-19 to be accomplished rapidly and effectively. One of these approaches that allow us to overcome drug development boundaries and obstacles is “drug repurposing”, also called drug repositioning, re-profiling, and re-tasking. Drug repurposing is a promising alternative strategy that allows revealing new therapeutic activities for existing approved drugs other than their main original indications.^6,7,8^ Many pharmaceutical companies pursue this strategy to circumvent expensive conventional drug discovery processes, hence reducing cost and time, which are considered crucial factors in catastrophic circumstances. This is because the safety and pharmacokinetic profiles of repositioned candidates are already established. Approximately one-third of approved drugs are introduced through drug repurposing.^9,10^

In drug repurposing methods, the other uses of drugs can be revealed using diverse approaches, including computational methods.^9,11^ Computational approaches play an important role during the drug discovery steps and development trajectory, helping researchers to reveal new promising drug candidates. Data analysis, modeling, and simulation embraced by computational approaches allow scientists to augment their research and advance drug discovery more quickly. An example of these *in silico* techniques is structure-based virtual screening and molecular docking studies.^12^ On the other hand, scientists can utilize bioinformatics to detect the main key genes from immense genomic data, and hence druggability will be easier to handle. Virtual screening can then identify new drug candidates based on the chemical properties of these drugs and their target proteins within a short time.^13^

Due to inflammatory cytokine storms it may provoke, COVID-19 treatment ought to comprise anti-inflammatory drugs to ensure an effective cure.^4^ Hence, glucocorticosteroids can be used in the treatment of COVID-19, due to their magical anti-inflammatory effect.^14^ Clinical investigations revealed that COVID-19 patients treated with a daily dose of 6 mg dexamethasone had reduced mortality by 8–26%. This may be particularly useful for short-term severely intubated COVID-19 patients.^14,15^ Furthermore, there is preliminary non-peer-reviewed evidence suggesting that ciclesonide may have the ability to reduce coronavirus RNA replication owing to its activity against NSP15.^16^

The main SARS-CoV-2 protease, 3-chymotrypsin-like protease (3CLpro), also known as M^pro^, is essential for the virus proteolytic maturation and replication. This main protease is important for the cleavage of the polyproteins to give some essential functional proteins, such as RNA polymerase, endoribonuclease, and exoribonuclease. Furthermore, the human proteases play a key role in the attachment of SARS-CoV-2 to its host cell through the virus spike glycoprotein.^17,18^

**Fig. 1:**
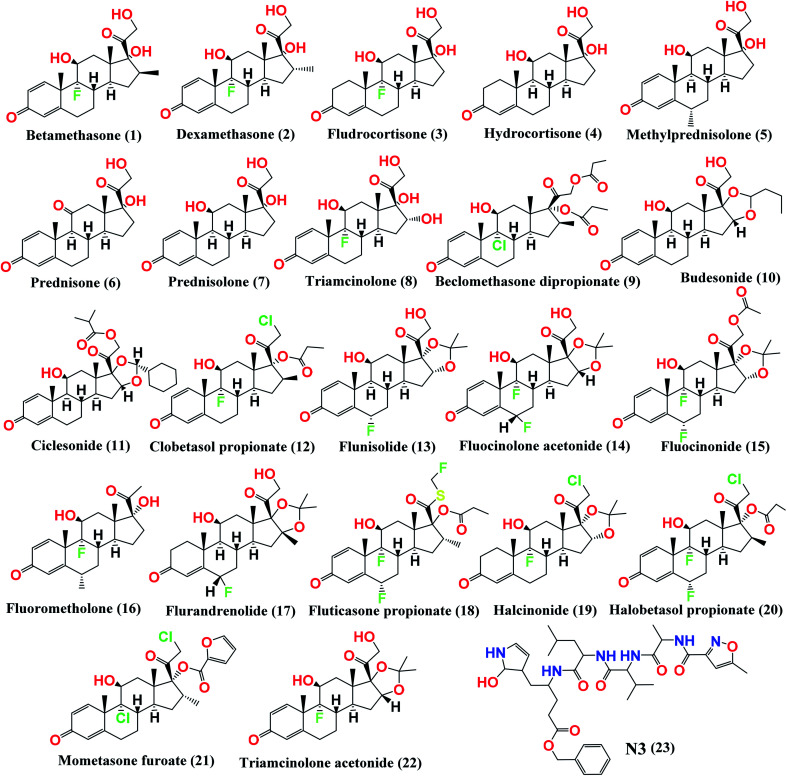
Chemical structures of the screened FDA-approved glucocorticosteroids (1–22) tested against the COVID-19 virus main protease and the co-crystallized inhibitor (N3, 23).

Therefore, in a continuation of our previous work concerning COVID-19,^19,20,21^ we aimed to utilize the crystal structure of the M^pro^ (PDB ID: 6LU7) with a small library of the approved glucocorticosteroids (depicted in Fig. 1) by conducting virtual screening, molecular docking, and quantum mechanical calculations.^22^ Thus, we aimed to investigate the best glucocorticosteroids that might have antiviral activity against COVID-19, or at least to prioritize the best members of the glucocorticosteroids to be used in the short-term treatment of inflammation in COVID-19 patients. In addition, we aimed to investigate the structural and electronic properties of the most promising candidate, ciclesonide.

Molecular dynamics (MD) simulations were also carried out on the docked drug–protein complexes to get a deep understanding of the affinity between the ligands and the COVID-19 main protease active site in the explicit solvent model to estimate the stability of the drugs within the active site of the protein. These drug–protein complexes were then subjected to molecular mechanics/generalized Born and surface area (MM/GB-SA) calculations to estimate the corresponding relative binding free energies.

## 2. Materials and methods

Molecular docking studies using the MOE 2014 suite,^23^ molecular dynamics simulation studies using the Desmond simulation package of Schrödinger LLC,^24^ and DFT calculations using Gaussian/g09 software^25^ were carried out to examine and confirm the binding affinities and modes of the 22 selected FDA-approved glucocorticosteroids against the COVID-19 main protease compared to its N3 inhibitor as a reference. The 22 approved glucocorticoid drugs were selected based on their structural similarity except for having different substitutions at the 16- and 17- positions on the glucocorticoid moiety.

### 2.1. Docking studies

#### 2.1.1. Preparation of the tested glucocorticoids

The 3D structures of the 22 tested glucocorticoids were downloaded from the PubChem website (https://pubchem.ncbi.nlm.nih.gov/), and prepared as described earlier.^21^ A molecular database containing all of the tested compounds and the co-crystallized N3 inhibitor was prepared as an MDB file for the docking process.

#### 2.1.2. Preparation of the SARS-CoV-2 main protease

The crystal structure of the SARS-CoV-2 main protease (M^pro^) was download from the Protein Data Bank (code 6LU7).^22^ It was protonated and hydrogen atoms with their standard 3D geometry were added, and then automatic corrections for any errors in the connection and for the type of atoms, and potential fixation of the receptor atoms were done.

#### 2.1.3. Docking of the tested glucocorticoids to the viral main protease binding site

The aforementioned database containing the tested glucocorticoids and the co-crystallized N3 inhibitor was docked. The methodology was carried out step wise as previously described.^26^ The MDB file containing the 23 ligands was loaded and general dock calculations were performed accordingly. For each ligand, we selected one pose where the combined binding affinity score, ligand–pocket interactions, and the rmsd_refine values were thought to represent the optimum structure of the ligand–protein complex. Visualization of the selected poses was carried out using PyMOL-2.4.0.^27^

Moreover, in the beginning, a validation process was carried out by docking the native N3 inhibitor inside its pocket of the target main protease (M^pro^) and a valid performance was indicated (RMSD = 1.23 Å) between the docked and crystal conformations.^28,29^

### 2.2. Molecular dynamics simulations

Molecular dynamics simulations were applied using the Desmond simulation package of Schrödinger LLC.^24^ The NPT ensemble with the temperature of 300 K and pressure of 1 bar was applied in all the runs. The simulation length was 100 ns with a relaxation time of one ps for all the selected ligands. The OPLS3 force-field specifications were utilized in all the simulations.^30^ The cutoff radius in Coulomb interactions was 9.0 Å. The orthorhombic periodic box boundaries were set 10 Å away from the protein atoms. The water molecules were explicitly described using the transferable intermolecular potential with a three-point (TIP3P) model.^31,32^ The salt concentration was set at 0.15 M NaCl and established using the system builder utility of Desmond.^33^ The Martyna–Tuckerman–Klein chain coupling scheme with a coupling constant of 2.0 ps was used for controlling the pressure and the Nosé–Hoover chain coupling scheme was used for the surveillance of the temperature.^34,35^ Nonbonded forces were calculated using a RESPA integrator and every step of the short-range forces were updated, whereas the long-range forces were updated every three steps. The trajectories were saved at 20 ps intervals for analysis. Analysis of the behavior and interactions between the ligands and protein were performed using the simulation interaction diagram tool implemented in the Desmond MD package. The stability of the MD simulations was kept an eye on by monitoring the RMSD of the ligand and protein atom positions over time.

### 2.3. MD trajectory analysis and prime MM-GBSA calculations

The simulation interactions diagram panel in the Maestro software was utilized to follow the interactions' contributions to the ligand–protein stability. Hence to calculate the ligand binding free energies and ligand strain energies for the docked compounds, the molecular mechanics generalized Born/solvent accessibility (MM-GBSA) was carried out over the 100 ns period with the thermal_mmgbsa.py python script provided by Schrödinger, which takes a Desmond trajectory file, splits it into individual snapshots, runs the MM-GBSA calculations on each frame, and outputs the average computed binding energy along with the standard deviation.

### 2.4. Quantum mechanics calculations details

Becke's three-parameter hybrid exchange–correlation functional (B3LYP)^36,37^ with different basis sets was employed in the quantum mechanics calculations. The structure of ciclesonide was initially optimized at B3LYP coupled with the 3-21G, 6-31G, 6-311G, and 6-311G* basis sets. The geometry of ciclesonide was finally optimized at B3LYP/6-311+G* where the geometries, charges, and all other structural properties of the ground and excited state structure of ciclesonide were computed using DFT and TD-DFT methods, respectively, using the B3LYP/6-311+G* model. No imaginary frequencies were detected for the optimized structures, indicating that the corresponding geometry was a true local minimum structure. An implicit solvent effect was considered in our calculations to replicate the reported UV-vis absorption spectrum of ciclesonide. Hence, the conductor-like polarizable continuum model (CPCM)^38^ utilizing the dielectric constant of methanol was used. All the calculations were performed using GAUSSIAN 09 Revision C.01 (ref. 25) on Swinburne supercomputing facilities.

## 3. Results and discussion

### 3.1. Docking study

Beside a Cys–His catalytic dyad it holds, the COVID-19 main protease substrate-binding pocket is found in a cleft between domains I and II. The N3 inhibitor shows asymmetric units containing only one polypeptide and is stabilized inside the substrate-binding site. Molecular docking of the picked glucocorticoids (1–22) and N3 inhibitor 23 (depicted in Fig. 1) into the M^pro^ active site was done. They were fitted at the inhibitor-binding pocket by several nonconstant interactions (Table 1). The order of strength according to their binding scores was: N3 inhibitor (23, docked) > ciclesonide (11) > fluorometholone (16) > dexamethasone (2) > betamethasone (1) > halobetasol propionate (20) > hydrocortisone (4) > fludrocortisone (3) > triamcinolone (8) > fluticasone propionate (18) > methylprednisolone (5) > fluocinonide (15) > prednisolone (7) > budesonide (10) > beclomethasone dipropionate (9) > fluocinolone acetonide (14) > triamcinolone acetonide (22) > flurandrenolide (17) > mometasone furoate (21) > halcinonide (19) > flunisolide (13) > prednisone (6) > clobetasol propionate (12).

**Table 1:** Receptor binding scores and amino acid interactions of the tested glucocorticoids and N3 inhibitor into the binding site of the COVID-19 main protease

No.	Glucocorticosteroid	S score^a^ kcal mol^−1^	RMSD_Refine^b^	Amino acid bond	Distance Å
1	Betamethasone	−16.88	0.69	Glu166/H-acceptor	1.70
				Glu166/H-donor	1.60
				Gln189/H-acceptor	2.00
				Thr26/H-donor	2.20
2	Dexamethasone	−17.26	1.02	Glu166/H-donor	1.80
				Gln189/H-acceptor	1.70
				Gln189/H-acceptor	1.60
3	Fludrocortisone	−16.30	1.33	Glu166/H-donor	1.70
				Glu166/H-acceptor	1.50
4	Hydrocortisone	−16.70	0.98	Glu166/H-acceptor	1.50
				Ser144/H-donor	2.90
				His163/H-donor	1.90
5	Methylprednisolone	−15.50	1.66	Glu166/H-acceptor	2.70
				Asn142/H-acceptor	2.00
6	Prednisone	−11.45	1.12	Glu166/H-acceptor	1.30
				Glu166/H-donor	2.00
7	Prednisolone	−14.91	1.10	Thr26/H-donor	2.00
				Thr26/H-acceptor	2.00
				Asn142/H-acceptor	1.70
8	Triamcinolone	−16.26	1.21	Gln189/H-acceptor	1.70
				Glu166/H-acceptor	1.40
				Phe140/H-acceptor	2.10
9	Beclomethasone dipropionate	−13.98	1.47	Glu166/H-donor	1.80
				Gln189/H-acceptor	2.60
				Thr26/H-donor	1.90
10	Budesonide	−14.06	0.51	Glu166/H-donor	2.60
				His163/H-acceptor	2.10
				Gly143/H-donor	2.40
				Ser46/H-donor	2.60
11	Ciclesonide	−18.88	1.56	Ser46/H-donor	1.80
12	Clobetasol propionate	−9.57	1.78	—	—
13	Flunisolide	−12.66	1.00	Thr26/H-acceptor	1.60
				Thr26/H-donor	3.10
				Gly143/H-donor	2.50
				Asn142/H-acceptor	2.0
14	Fluocinolone acetonide	−13.98	1.77	Asn142/H-acceptor	1.80
15	Fluocinonide	−15.38	1.62	—	—
16	Fluorometholone	−17.45	1.68	Asn142/H-acceptor	1.50
17	Flurandrenolide	−13.15	1.50	Gln189/H-acceptor	1.70
				His164/H-acceptor	1.60
18	Fluticasone propionate	−15.88	1.09	Gln189/H-acceptor	1.90
				His41/H-donor	3.20
19	Halcinonide	−12.81	1.77	Glu166/H-donor	2.00
20	Halobetasol propionate	−16.80	1.43	Asn142/H-acceptor	1.80
21	Mometasone furoate	−13.02	1.55	His163/H-donor	2.20
				Asn142/H-donor	1.90
22	Triamcinolone acetonide	−13.21	1.68	Glu166/H-donor	2.70
				Glu166/H-acceptor	1.30
23	N3	−22.71	1.65	Glu166/H-acceptor	1.62
				Glu166/H-acceptor	1.88
				Glu166/H-donor	1.52
				Gln189/H-acceptor	1.50
				Thr190/H-acceptor	2.10
				Phe140/H-acceptor	1.91
				His164/H-acceptor	1.62
				His41/H-donor	2.01

a S: the score of a compound positioned into the binding pocket of the protein utilizing the London Δ*G* scoring function.

b RMSD_Refine: the root-mean-squared-deviation (RMSD) between the refined predicted pose and those of the unrefined crystal structure.

Although many poses were gained for each selected compound inside the receptor pocket with even better binding modes and/or interactions, the poses with the best scores (indicating the stability of the pose) and rmsd_refine values (indicating the proximity of the elected pose to the position of the authentic ligand inside the receptor pocket) were selected. The scores, RMSD values, and different binding interactions with the COVID-19 M^pro^ pocket amino acids are presented in Table 1 and their detailed figures are included in ESI data 1 (Fig. S1†).

By analyzing the docking results of the selected glucocorticoids, it was found that most of the selected compounds manifested very close binding scores and modes compared to the co-crystallized inhibitor (N3) at the COVID-19 M^pro^ target receptor. Ciclesonide 11, dexamethasone 2, betamethasone 1, hydrocortisone 4, fludrocortisone 3, and triamcinolone 8 were found to have the best binding affinities and modes against COVID-19 protease with binding scores of −18.88, −17.26, −16.88, −16.70, −16.30, and −16.26 kcal mol^−1^, respectively (Table 1). These energy values were very close to that of the docked N3 inhibitor (binding energy = −22.71 kcal mol^−1^). The detailed binding modes of the docked N3 23 and all of the tested glucocorticoids (1–22) are presented in Table 1. Furthermore, all of their 3D binding interactions, surfaces and maps, and 3D positioning inside the protein pocket can be found in ESI data 1 (Fig. S1–S3†). Also, the 3D binding interactions and 3D protein positioning of the best selected six glucocorticoids are presented in Table 2.

**Table 2:** The 3D view of binding interactions and the 3D positioning between the tested glucocorticoid drugs and N3-binding pocket within the COVID-19 protease compared to the N3 (docked). Red dashed lines refer to hydrogen bonds

Drug	3D interaction	3D protein positioning
Ciclesonide 11	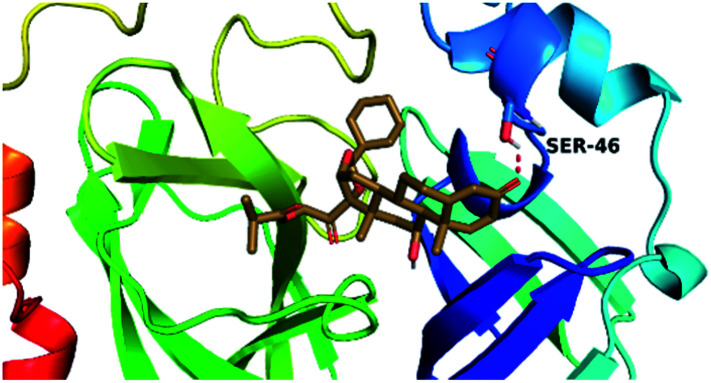	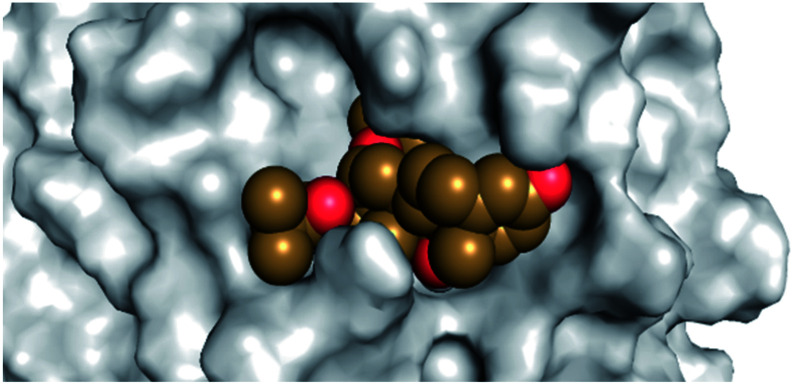
Dexamethasone 2	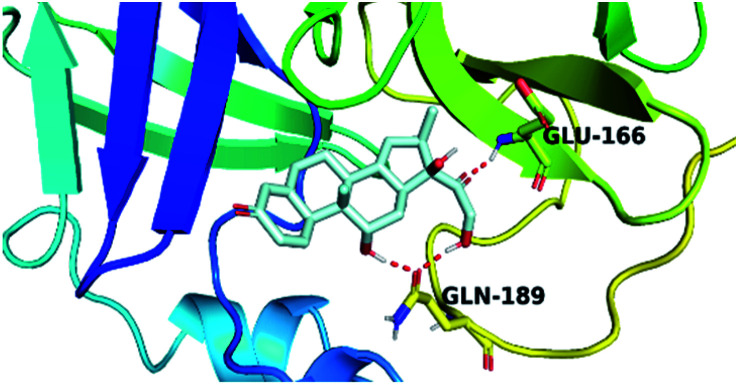	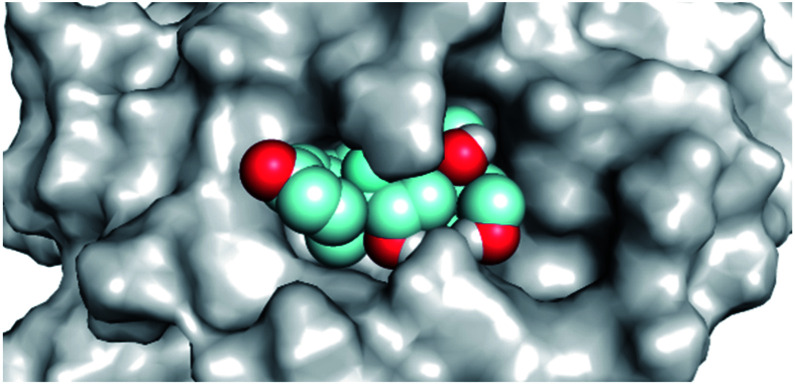
Betamethasone 1	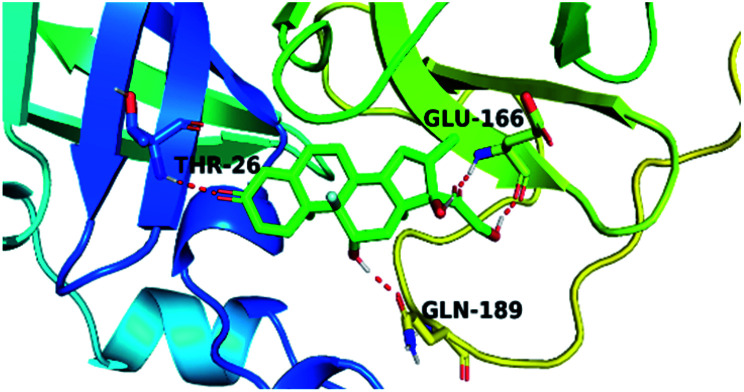	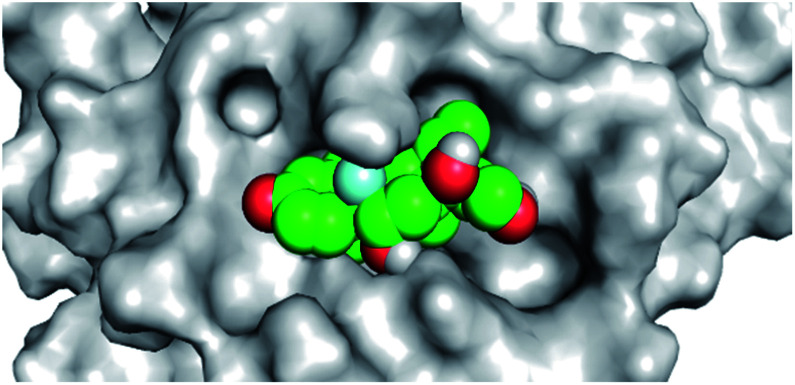
Hydrocortisone 4	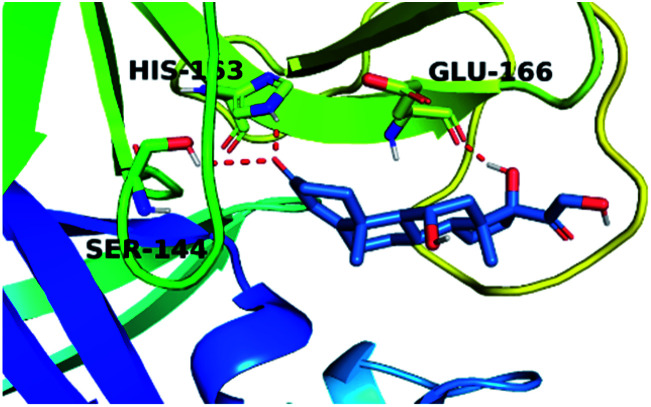	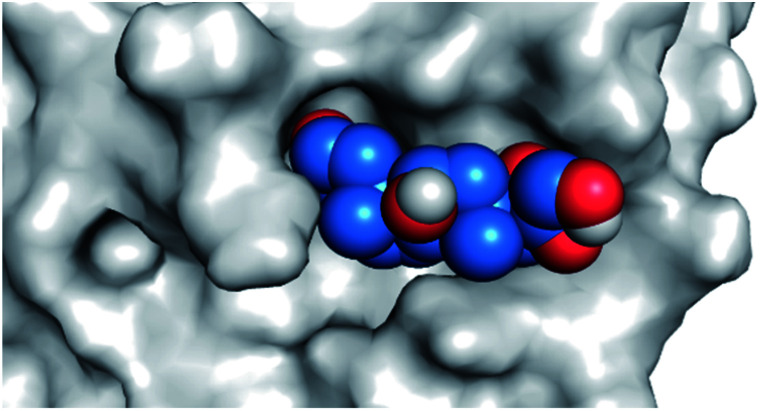
Fludrocortisone 3	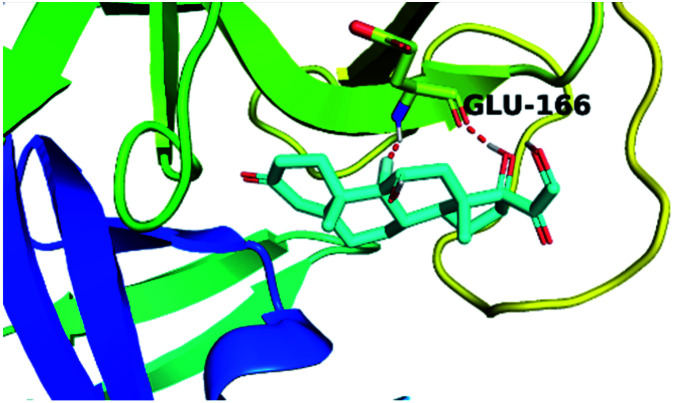	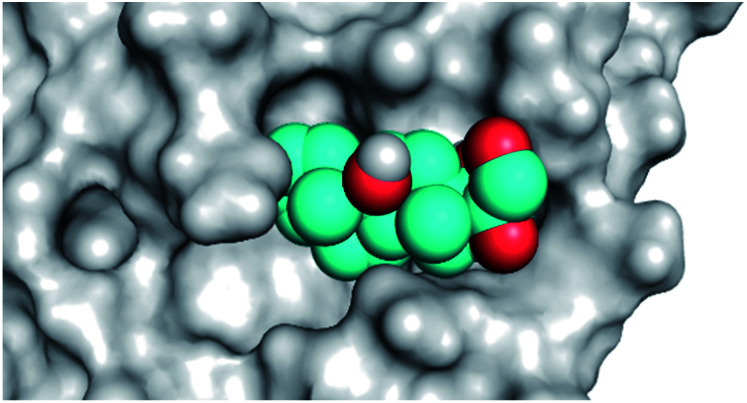
Triamcinolone 8	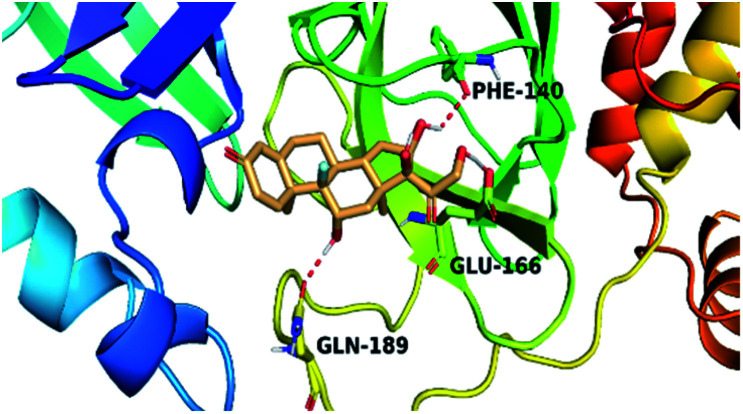	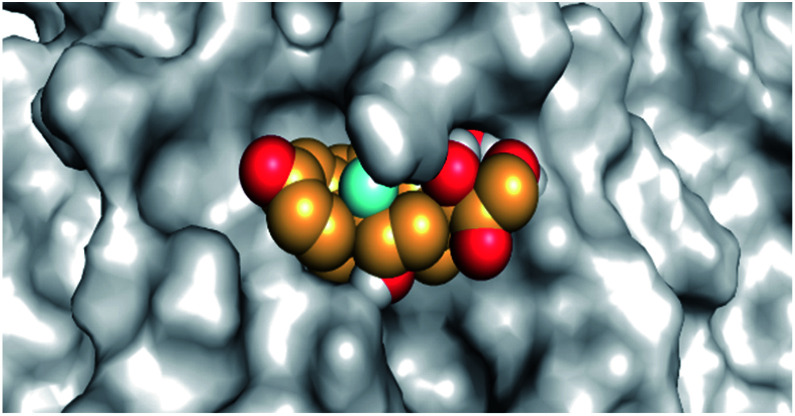
N3 23	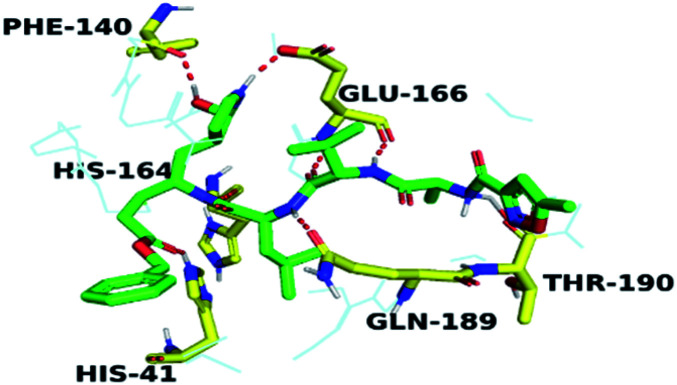	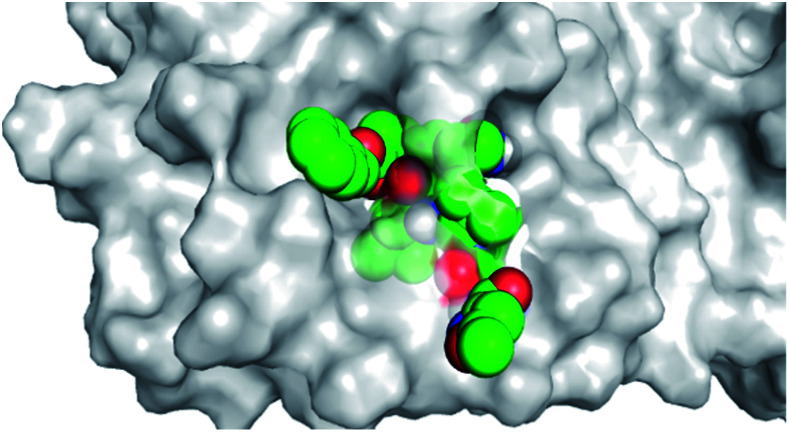

### 3.2. Molecular dynamics (MD) simulation

Despite their usual fast and approximate utility, docking protocols lack protein flexibility, which may be related to the thoroughness of the resulting ligand–protein complexes. Therefore, docking is often accompanied by the more computationally expensive but more accurate molecular dynamic (MD) simulations techniques to provide a better complementary result. In summary, MD simulations can be used to study the macromolecule features, but it counts on classical mechanics and the use of Newton's equation of motion to calculate the position of and speed of each atom of the studied system.

Therefore, we can say that MD can perform a more rigorous conformational search than docking does, thus providing a more accurate representation of protein motion. Hence, MD simulations were carried out utilizing the Desmond package on the ligand–potential complex to imitate the interaction of the best selected six candidates (ciclesonide 11, dexamethasone 2, betamethasone 1, hydrocortisone 4, fludrocortisone 3, and triamcinolone 8) selected from the docking study with the COVID-19 main protease active site for 100 ns.

#### 3.2.1. Protein and ligand RMSD analysis

**Fig. 2:**
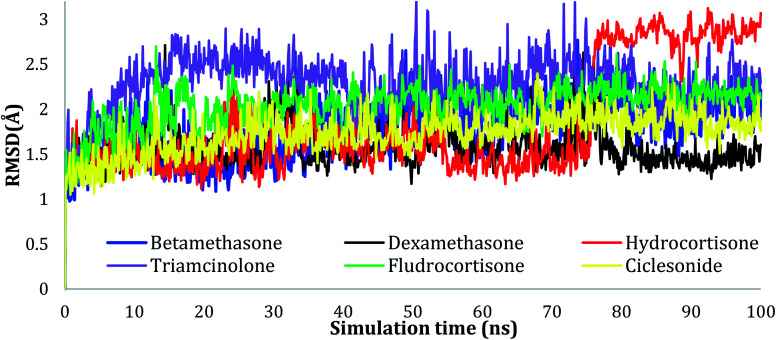
Plots of RMSD for Cα atoms (Å) for the initial structure *vs.* the simulation time (ns) for all the complexes.

The RMSD values of Cα atoms were evaluated for all the complexes to monitor the effect of the compounds on the conformational stability of 6LU7 during the simulations, taking into consideration the initial structure. The results were plotted as a function of the simulations time, as seen in Fig. 2. As can be seen in the plots, all the complexes tended to reach their stable states after 25 ns, and the fluctuation of the proteins was within acceptable variation with RMSD values of less than 3.00 Å, indicating the stability of the protein conformation.

**Fig. 3:**
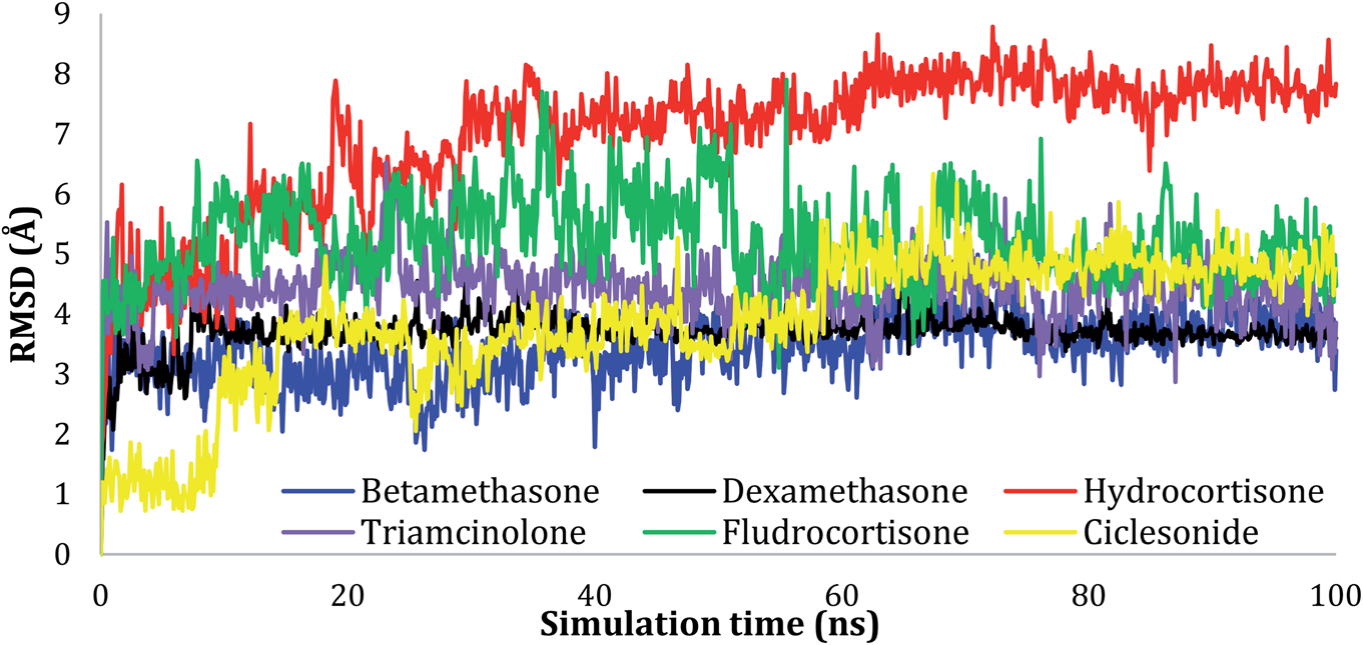
Plots of RMSD for ligand atoms (Å) concerning the initial structure *vs.* simulation time (ns) for all the complexes.

The RMSD values of the ligands were also plotted as a function of simulation time to show the RMSD of a ligand aligned and measured just on its reference confirmation within the active site, and as can be seen from Fig. 3, betamethasone 1, dexamethasone 2, and triamcinolone 8 were stable within the active site of the protein and reached equilibrium in the first 20 ns. Their average RMSD values over the last 50 ns were 3.74, 3.75, and 4.43 Å respectively. Ciclesonide 11 and fludrocortisone 3 showed equilibrium stability at about 60 ns and 70 ns, with RMSD values in the last 50 ns found to be 4.68 Å and 5.10 Å, respectively. Hydrocortisone 4 was the most fluctuate ligand among others, it only reached equilibrium at 80 ns with an RMSD of 7.70 Å. The RMSD of all the ligands over all the simulation times and the last 50 ns are reported in Table 1SI, ESI data 2.†

**Fig. 4:**
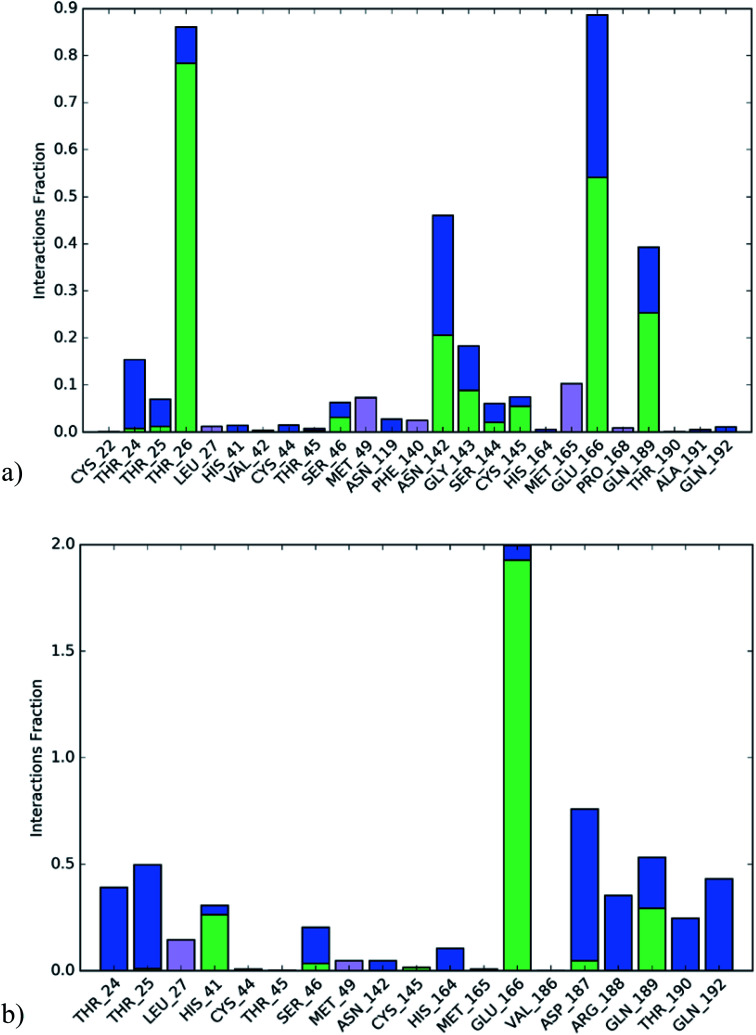
The histogram of ligand-6LU7 contact throughout the trajectory. (a) Ciclesonide, (b) Betamethasone.

**Fig. 5:**
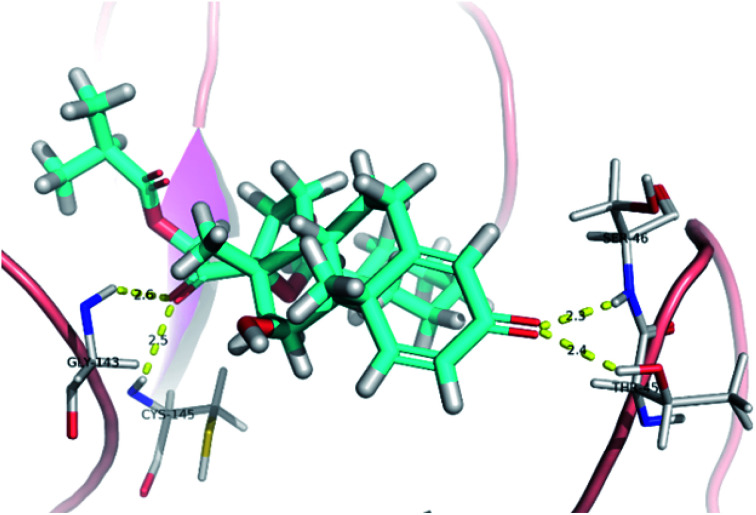
Snapshot of ciclesonide interactions with the protein residues (hydrogen bonds are shown as yellow dashed) at 100 ns.

The active site contains the following polar amino acids (threonine (Thr26), asparagine (Asn142), and glutamine (Gln189, Gln192)), a nonpolar amino acid (alanine (Ala193)), and negatively charged amino acids (glutamic (Glu166) and aspartic acid (Asp187)). Since ciclesonide 11 and betamethasone 1 showed the highest binding score and highest MM-GBSA energy (Tables 1 and 3), hence their interactions will be discussed in detail. As can be seen from Fig. 4, the histogram explains the contacts that occur during the simulations between the ligands and protein, which were generated with simulation interactions, through a diagram panel implemented in Maestro software. For ciclesonide 11, hydrogen bonding between the Thr26 and Glu166 residues and the ligand were maintained during most of the time, either directly or through water bridging H-bonds as can be seen in Fig. 5. Other residues such as Asn142 and Gln189 were able to develop an H-bond for almost 50% of the time, again, directly or through a water bridge. Hydrophobic interactions with Met49 and Met165 were very weak and could be nongalactic (Fig. 4a). Betamethasone 1, was able to form a donor–acceptor hydrogen bond with Glu166, leading to 180% of the time, 90% of the time as donor and 90% as acceptor, while other hydrogen bonds were formed with His41 and Gln189 during 50% and 40% of the simulation time, respectively. Bridging hydrogen bonds through water were formed with Thr24, Thr25, Asp187, and Gln192 between 40–75% of the simulation time as can be seen in Fig. 4b, other selected drug interactions histograms and figures are presented in the ESI data 2 file.†

**Fig. 6:**
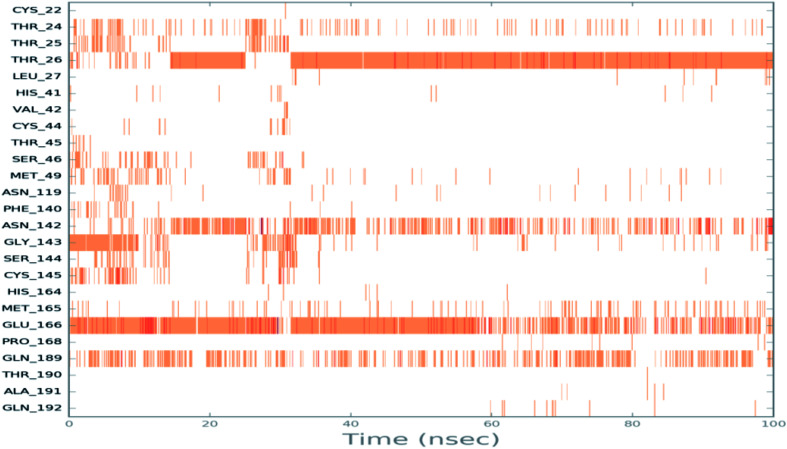
Ciclesonide–6LU7 interactions shown by the active site amino acids in each trajectory frame, white refers to zero interaction while the deep color indicates more interactions.

To monitor the protein–ciclesonide interactions during the simulation, a plot of active site residues was plotted against trajectories frames (Fig. 6). Notably, Thr26 and Glu166, Asn142, and Gln129 were in contact with ciclesonide during most of the time. While, for example, Gly143 had a strong interaction with ciclesonide at the beginning of the simulation time, and then it was lost at about 10 ns.

#### 3.2.2. Ligand properties

**Fig. 7:**
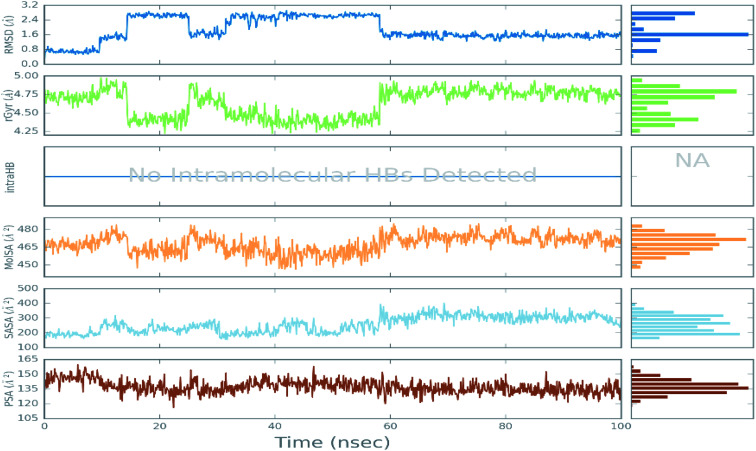
The ligand property trajectory of the ciclesonide–6LU7 complex during the 100 ns simulation.

Ligand features, including the RMSD, solvent accessible surface area (SASA), the radius of gyration (rGyr), intramolecular hydrogen bond, molecular surface area (MolSA), and polar surface area (PSA), are reported in Fig. 7. Other ligand properties are reported in the ESI data 2 file.† For ciclesonide 11, the root mean square deviation (RMSD) concerning its atoms' initial positions fluctuated up to 60 ns of simulation time before reaching equilibrium at around 1.6 Å. The rGyr, which measures the extendedness of a ligand, was equal to its principal moment of inertia. The rGyr of the ligand also showed a heavy fluctuation of up to 60 ns simulation and then gradually reached equilibrium at 4.75 Å. Ciclesonide 11 had no intramolecular hydrogen bonds. The molecular surface (MolSA) was calculated with a 1.4 Å probe radius, with this value equivalent to a van der Waals surface area of a water molecule. The MolSA fluctuated over most of the simulation time before reaching equilibrium at 60 ns at around 470 Å^2^. The solvent accessible surface area (SASA), which is the surface area of a molecule accessible by a water molecule, fluctuated between 200 and 400 Å^2^, before reaching equilibrium at 300 Å^2^, indicating that almost one side of the molecule (volume = 529 Å^2^) was water accessible during the simulations. Finally, the polar surface area (PSA), representing the solvent-accessible surface area in a molecule, was contributed only by oxygen and nitrogen atoms, as can be seen in Fig. 7. This oscillated between 120 and 150 Å^2^ before equilibrating at around 135 Å^2^. The ligand properties showed some fluctuation at the beginning of the simulation before reaching equilibrium, indicating the stability of ciclesonide 11 to the active site of the COVID-19 main protease active site.

### 3.3. MM-GBSA study

Here, 200 snapshots were selected for further analysis taken within a 50 ps interval after calculating the average binding energy for the equilibrated MD trajectory. The binding energy was calculated using the previously mentioned equations.^39^ The average MM-GBSA binding energy was created using the thermal_mmgbsa.py python script provided by Schrödinger, which also produces the lipophilic energy, Coulomb energy, van der Waals energy, generalized Born electrostatic solvation energy, covalent binding energy, and hydrogen-bonding energy. All the obtained data are listed in Table 3.

From the MM-GBSA calculations, the most favored binding was exerted by both betamethasone 1 and ciclesonide 11 compared to the N3 ligand, with ciclesonide 11 showing highly favored van der Waals interactions and lipophilic energy compared to 1 (Table 3), while betamethasone 1 showed a more favorable Columbo energy. Since all the drugs awere aliphatic, no π– π interaction energy was reported.

**Table 3:** Prime MM-GBSA energies in kcal mol^−1^ for ligands binding at the active site of the COVID-19 main protease compared to the co-crystallized inhibitor (N3)^a^

	Δ*G* Binding	Coulomb	Covalent	H-bond	Lipo	Solv_GB	vdW	St. Dev.
Ciclesonide 11	−51.24	−11.00	1.57	−1.16	−14.32	19.33	−45.67	8.67
Betamethasone 1	−51.05	−19.48	0.86	−1.58	−12.53	21.71	−40.03	4.35
Hydrocortisone 4	−49.00	−15.59	2.19	−1.01	−14.05	20.84	−41.38	9.07
Fludrocortisone 3	−48.48	−15.46	1.58	−0.91	−12.98	18.66	−39.36	7.32
Triamcinolone 8	−47.53	−21.96	1.86	−1.49	−11.77	19.86	−34.03	9.56
Dexamethasone 2	−37.37	−15.83	1.27	−0.82	−10.36	22.99	−34.62	4.23
N3 23	**−80.00**	**−43.59**	**4.67**	**−3.45**	**−16.27**	**45.02**	**−64.12**	**6.62**

a Coulomb: Coulomb energy; covalent: covalent binding energy; vdW: Van der Waals energy; Lipo: lipophilic energy; Solv_GB: generalized Born electrostatic solvation energy; H-bond: hydrogen-bonding energy; St. Dev.: standard deviation.

### 3.3. Quantum mechanics studies

**Fig. 8:**
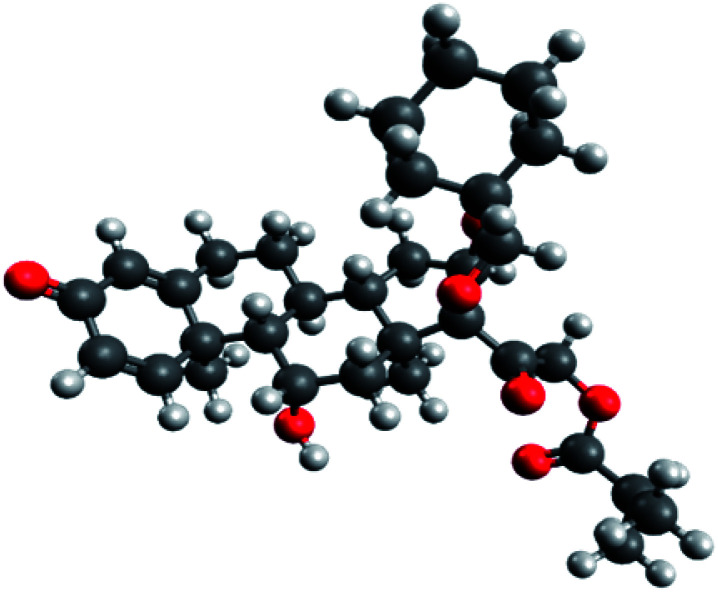
Optimized structure of ciclesonide obtained at the B3LYP/6-311G* level.

Since the molecular docking results revealed that ciclesonide 11 was the most promising candidate exhibiting COVID-19 main protease inhibitory activity, we therefore studied its structural and electronic configurations in detail. The chemical structures (2D and 3D) and nomenclature of ciclesonide (CAS 126544-47-6) are given in Fig. 8. It is obvious that ciclesonide possessed some chiral centers and rotatable single bonds, rendering it a flexible medium-size molecule (MW = 540.69 Da). In addition, ciclesonide contained a small system of conjugated double bonds found in one ring (labeled as ring A), see Fig. 8.

Ciclesonide's structure was energetically optimized at different levels of theory as described in the computational methods. By reviewing the literature, B3LYP coupled with different types of basis sets have been predominantly utilized for describing the glucocorticoid systems.^40,41^ Therefore, studies on ciclesonide 11 using B3LYP/6-311G* and B3LYP/6-311+G* are herein reported in our study. Geometries of the ciclesonide structure obtained at B3LYP/6-311G* and B3LYP/6-311+G* are deposited in the ESI data 1.† Some of the spatial and electronic parameters of ciclesonide are listed in Table 4.

**Table 4:** Comparison of the spatial and electronic parameters of ciclesonide calculated at the B3LYP/6-311G* and B3LYP/6-311+G* levels

		B3LYP/6-311G*	B3LYP/6-311+G*
Rot. Const.	A (GHz)	0.1080486	0.1080863
	B (GHz)	0.0614718	0.0608067
	C (GHz)	0.0442127	0.0438208
Spatial moment *r*^2^ (a.u.)		5216651.8603	5216696.0275
*E*_h_ (a.u.)		−1772.881464	−1772.909008
ZPE (a.u.)		0.719611	0.718603
*E*_h_ + ZPE (a.u.)		−1772.161853	−1772.190406
Polarizability (a.u.)		477.742460	512.314524
*μ* (D)		9.2967	10.1645
HOMO–LUMO gap (eV)		5.0764	5.1115
Entropy (cal mol^−1^ Kelvin)		224.240	225.821

The rotational constants parameter is a geometrical descriptor of the molecular size at vibrational equilibrium. The rotational constants at the A, B, and C states of ciclesonide were obtained at 0.1080486, 0.0614718, and 0.0442127 GHz (B3LYP/6-311G*), whereas nearly similar values were calculated at 0.1080863, 0.0608067, and 0.0438208 GHz using the B3LYP/6-311+G* method. The electronic spatial extent *r*^2^ (also called the spatial moment) conveys roughly the molecular size (volume) of a molecule. The values obtained by using either B3LYP/6-311G* or B3LYP/6-311+G* were nearly similar. Other parameters, such as the total energy (*E*_h_), zero-point energy (ZPE), polarizability, dipole moment (*μ*), HOMO–LUMO energy gap, and entropy, did not show significant discrepancies in the computed values, as can be seen from Table 4.

The main UV-vis absorption peak of ciclesonide was reported at 242 nm.^42,43^ The UV-vis peak using the B3LYP/6-311+G* model was calculated at 238 nm, showing a discrepancy from the reported value by only 4 nm; unlike for the B3LYP/6-311G* model, where the absorption peak was computed at 233 nm with a 9 nm difference from the reported value. This result suggests that the B3LYP/6-311+G* model is—most probably—the best model to describe the ground and transition states of ciclesonide. The computed absorption (excitation) energy, oscillator strength, and major electronic contributions of ciclesonide are listed in Table 5. The calculated UV-vis spectrum results are also shown in Table 6.

**Table 5:** Transitions of the main absorption band maximum of ciclesonide with an oscillator strength *f* > 0.10

Model	Absorption energy	Oscillator strength	Major contributions
6-311G*	233 nm	0.1998	H-5 → LUMO (43%)
			H-4 → LUMO (13%)
			H-2 → LUMO (20%)
6-311+G*	238 nm	0.2393	H-5 → LUMO (53%)
			H-4 → LUMO (12%)
			H-2 → LUMO (18%)

**Table 6:** Computed UV-vis absorption spectra and the electronic circular dichroism (ECD) spectra of ciclesonide

	B3LYP/6-311G*	B3LYP/6-311+G*
UV-vis	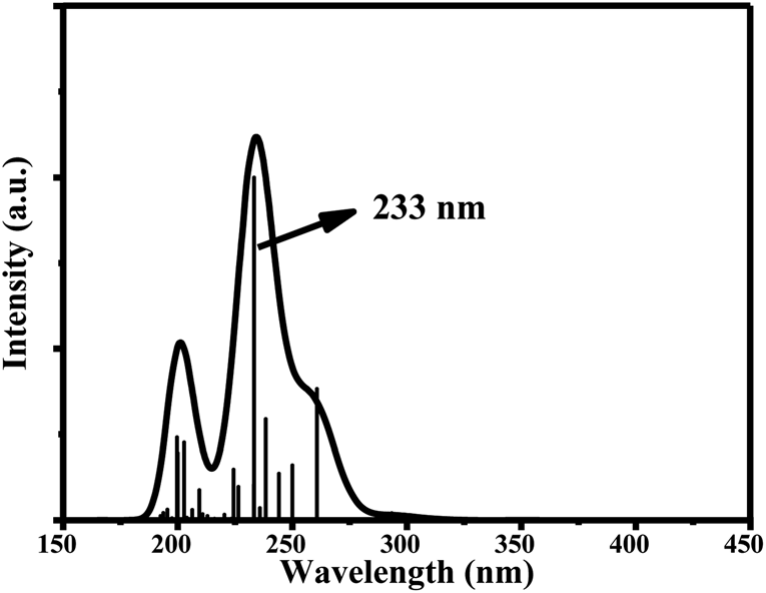	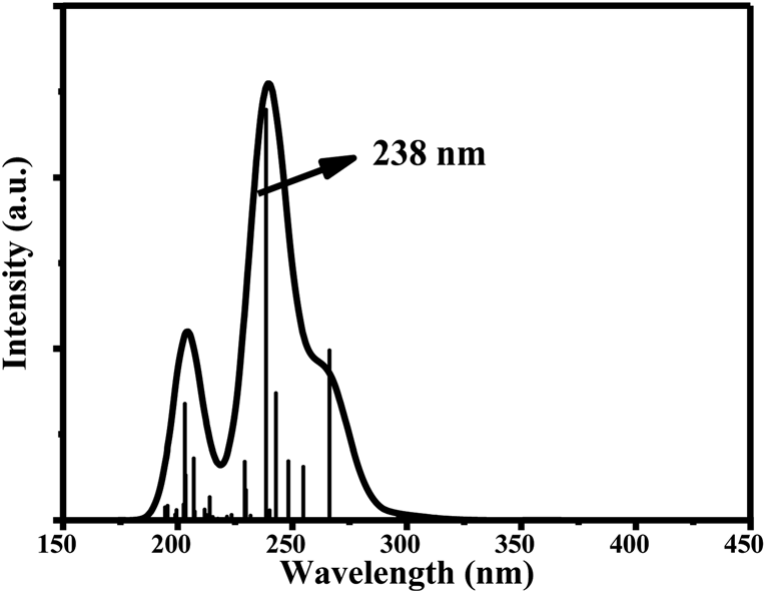
ECD	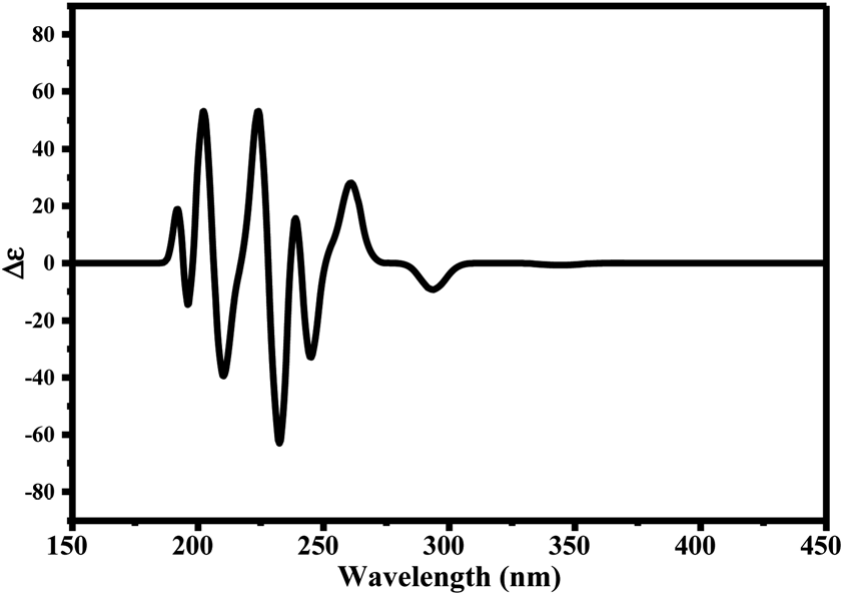	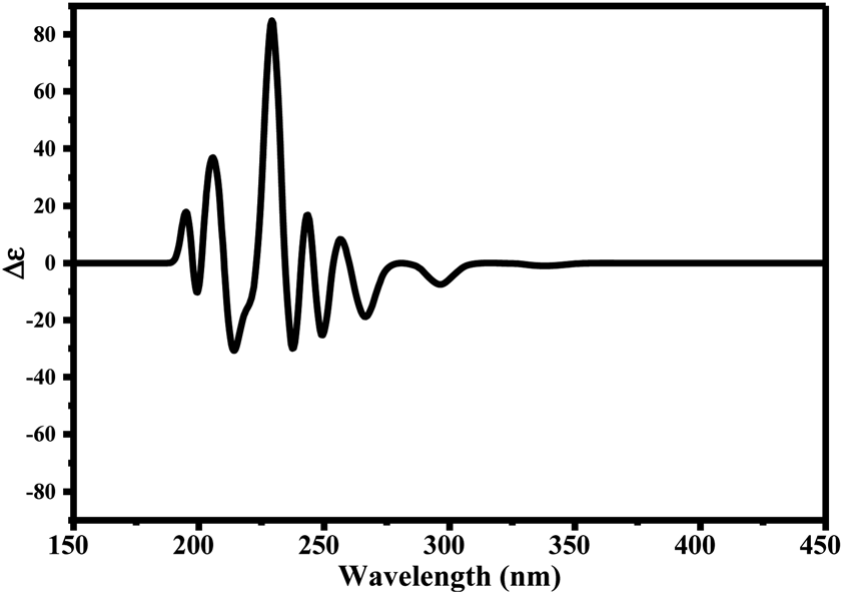

Since ciclesonide 11 possessed various chiral centers, it was important to calculate the electronic circular dichroism spectrum (ECD), which has not been reported in the literature to the best of our knowledge. The ECD spectrum measures the difference in absorbance of right- and left-circularly polarized light by a molecule rather than the commonly used absorbance of isotropic light as in UV-vis measurements.^44^ The ECD spectra of ciclesonide 11 calculated at the B3LYP/6-311G* and B3LYP/6-311+G* levels are reported for the first time, as depicted in Table 6.

It is known that the most energetically stable structure of a drug is not necessary the most biologically active form ref. 45, which can be attributed to the combined importance of the electronic configuration besides the geometrical configuration in determination of the binding interactions between a drug and its target protein. Therefore, the electrons distribution over some molecular orbitals were computed. The electron charge density of the highest occupied molecular orbital (HOMO) and lowest unoccupied molecular orbital (LUMO), besides the two outermost molecular orbitals (occupied and virtual) of ciclesonide 11, are depicted in Table 7.

**Table 7:** Charge density of the outermost molecular orbitals of ciclesonide along with the molecular electrostatic potential (MEP) map. HOMO refers to the highest occupied molecular orbital while LUMO indicates the lowest unoccupied molecular orbital

	B3LYP/6-311G*	B3LYP/6-311+G*
LUMO+2	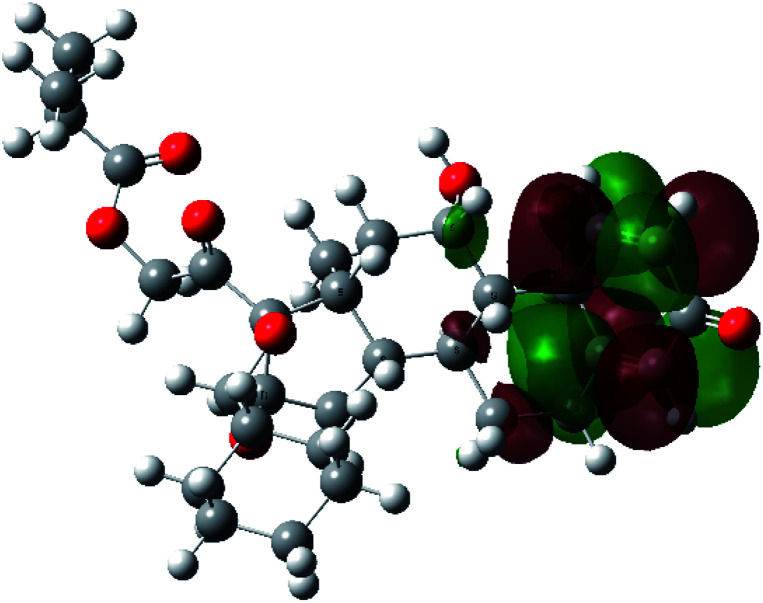	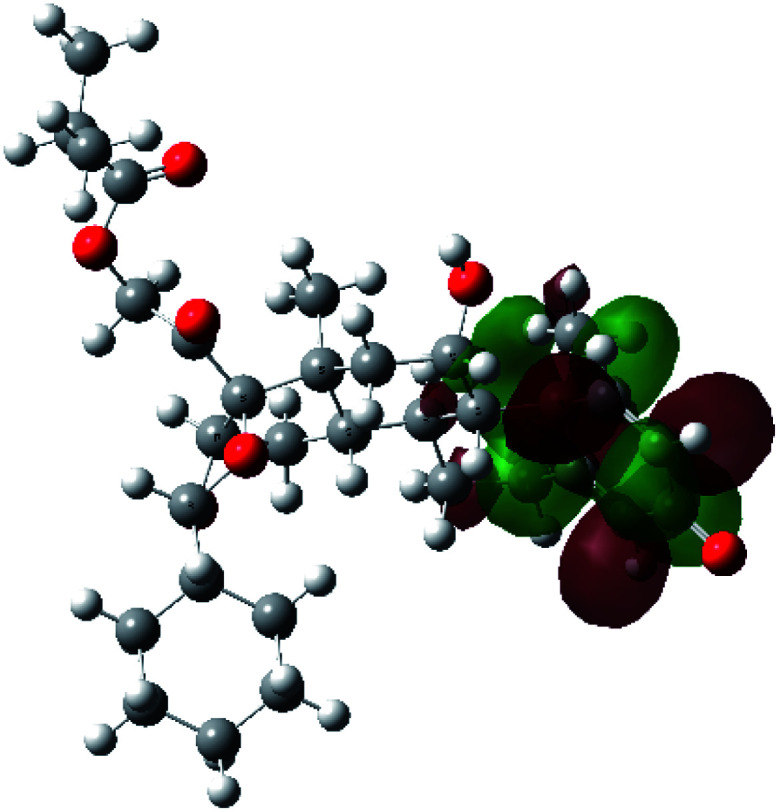
LUMO+1	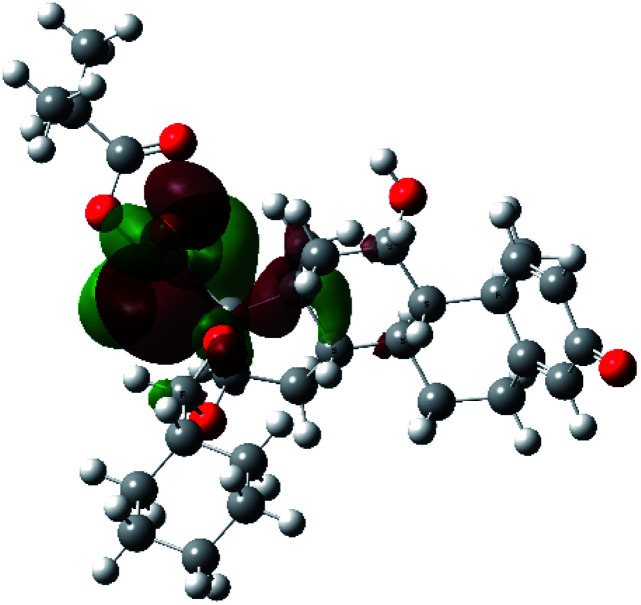	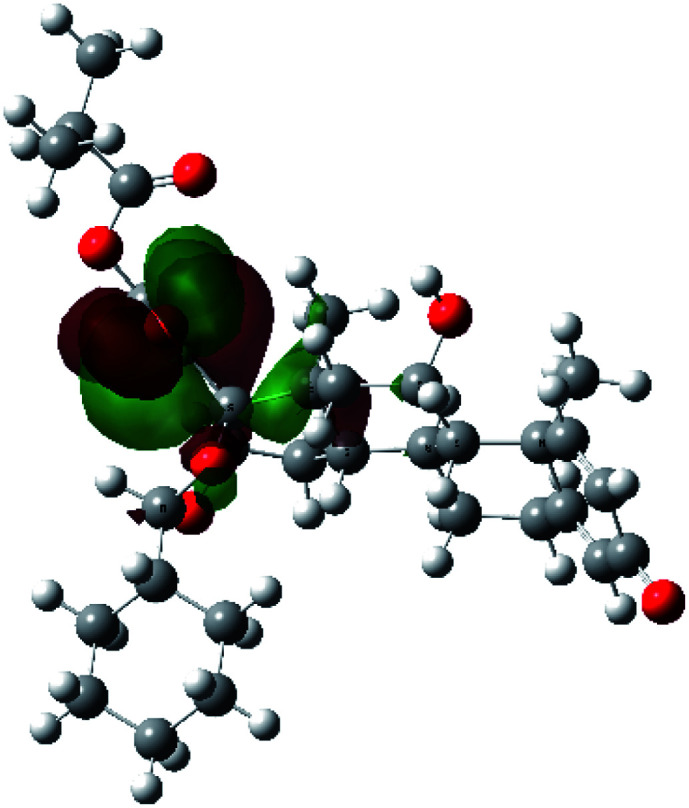
LUMO	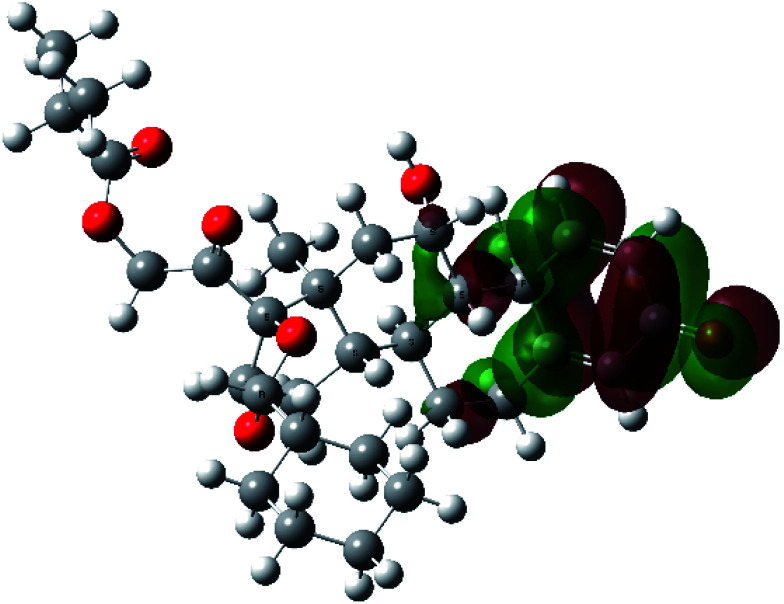	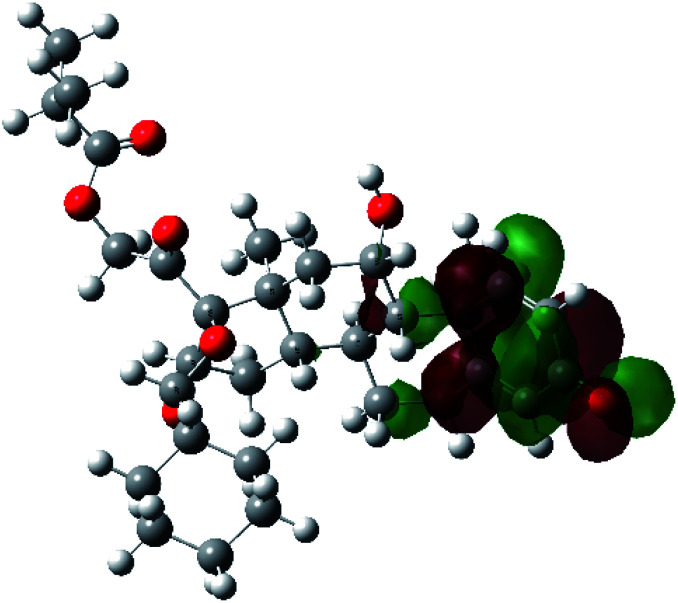
HOMO	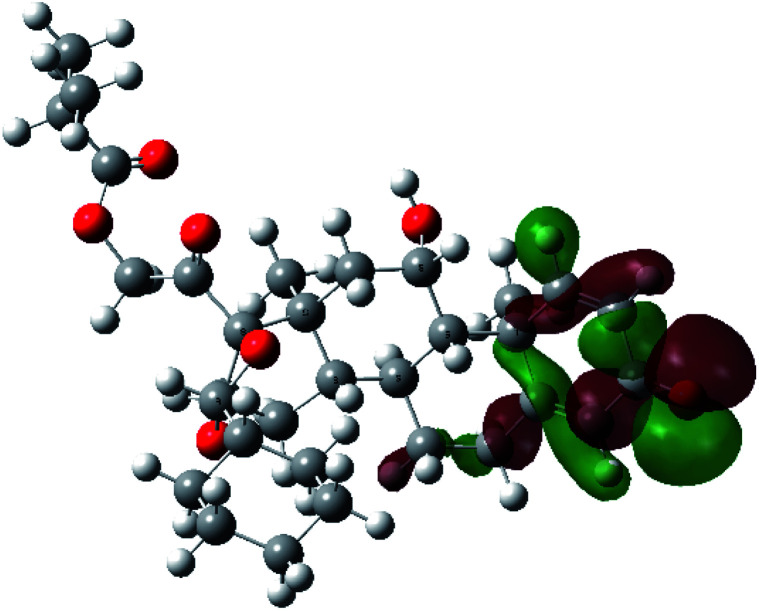	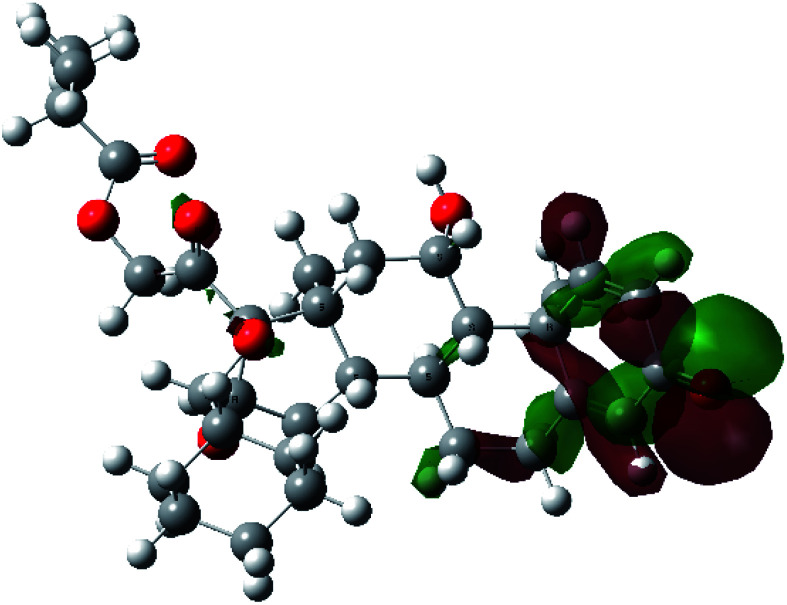
HOMO−1	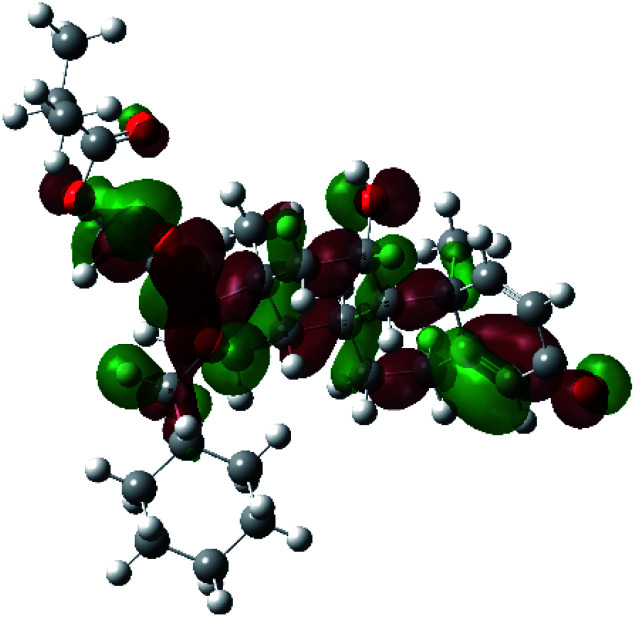	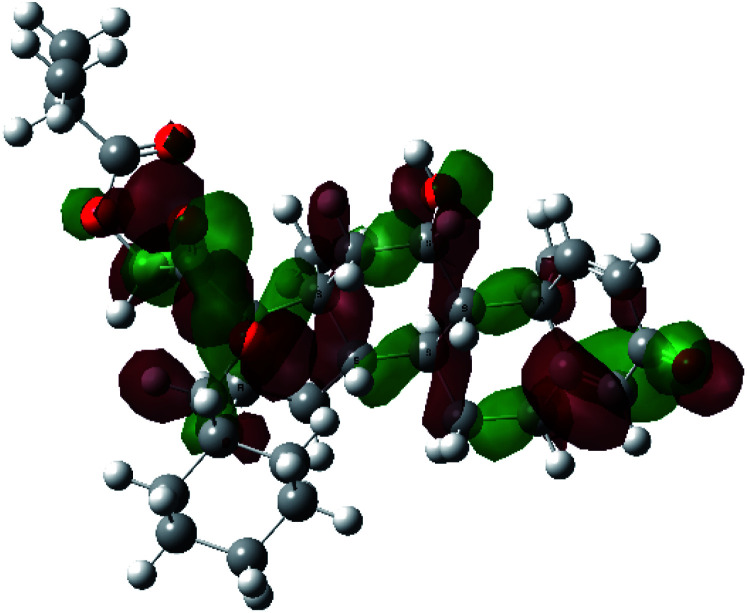
HOMO−2	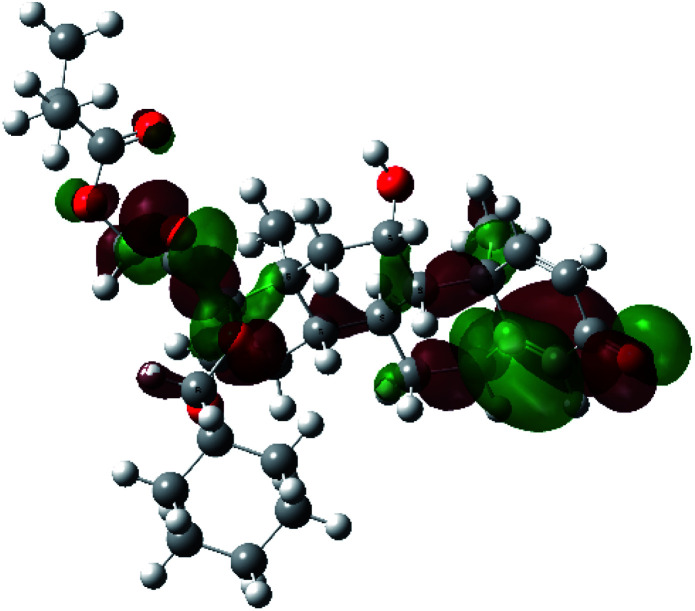	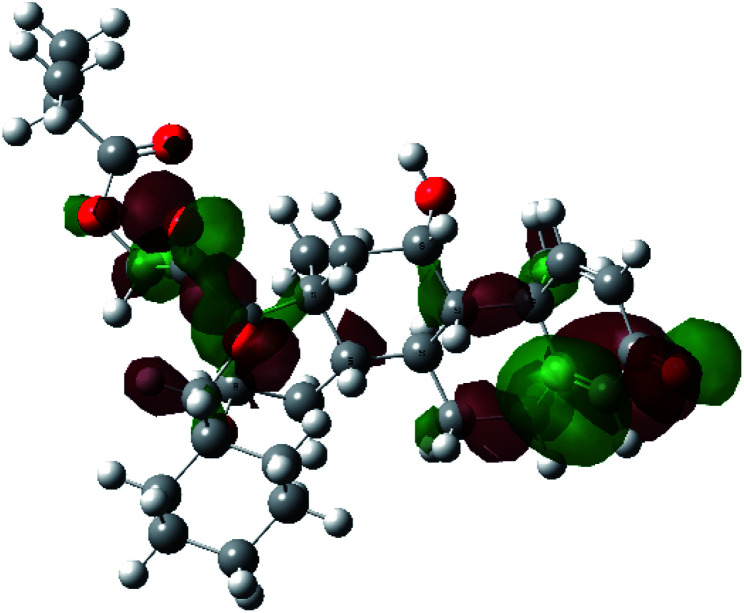
MEP	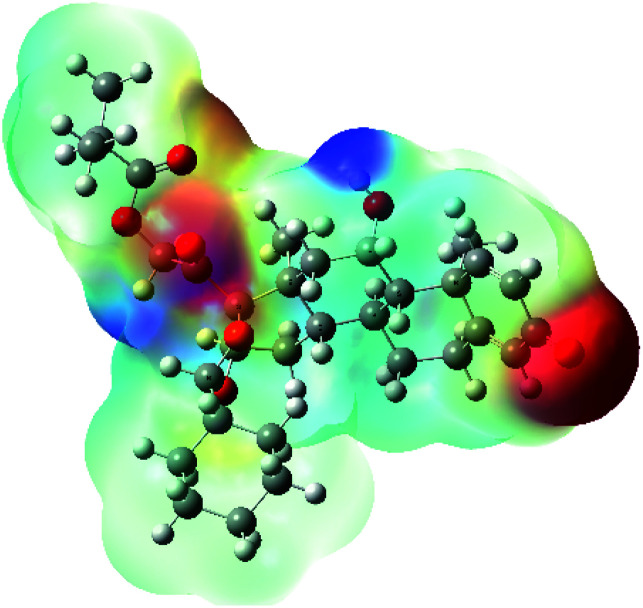	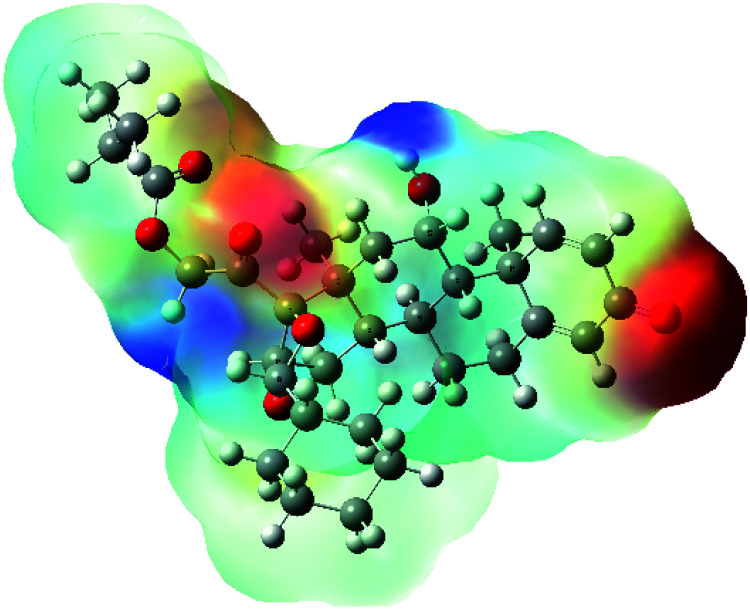

It was noted that the electron density is mainly localized on ring A at the HOMO of ciclesonide 11, while the electron density was slightly spread to the adjacent ring in the case of the LUMO. Since the HOMO and LUMO are the main contributors to the binding interactions between a drug and the target receptor, the obtained calculations postulate the occurrence of electron transfer from ring A to the adjacent ring. Further studies should be conducted to confirm the occurrence of such a charge transfer process, which is beyond the scope of our study.

The molecular electrostatic potential (MEP) map quantifies the electronic density distribution at a molecular level. MEP maps of ciclesonide 11 are depicted in Table 7. The electronic density distribution changes from the more electronegative (red) to the more electropositive (blue). It was found that the carbonyl group on ring A was electron rich and therefore a hydrogen bond with Ser46 was formed, as demonstrated by the molecular docking calculations. Another electronegative site was observed on the carboxylic group connecting an isobutyryl moiety to the main nucleus of ciclesonide. The observed electronegativity may account for the metabolic attack for cleavage of the isobutyryl group, resulting in formation of the active metabolite desisobutyryl–ciclesonide. It is noteworthy that the MD simulation revealed a transient binding interaction between that carboxylic group and Gly143. In addition to the two electron-rich sites, two electron-deficient sites were observed, which can act as hydrogen bond donor sites.

Overall, the conducted quantum mechanical calculations emphasized that the use of a proper computational model is crucial for obtaining an accurate description of the structural and electronic configurations of ciclesonide 11. In addition, QM calculations can provide us with quantitative analysis of physicochemical properties that cannot be experimentally measured, such as the electron density of molecular orbitals.

### 3.4. Structure–activity relationship

The study of the structure–activity relationship of the tested glucocorticoids (1–22) according to their binding affinities to the SARS-CoV-2 main protease showed the following interesting results.

**Fig. 9:**
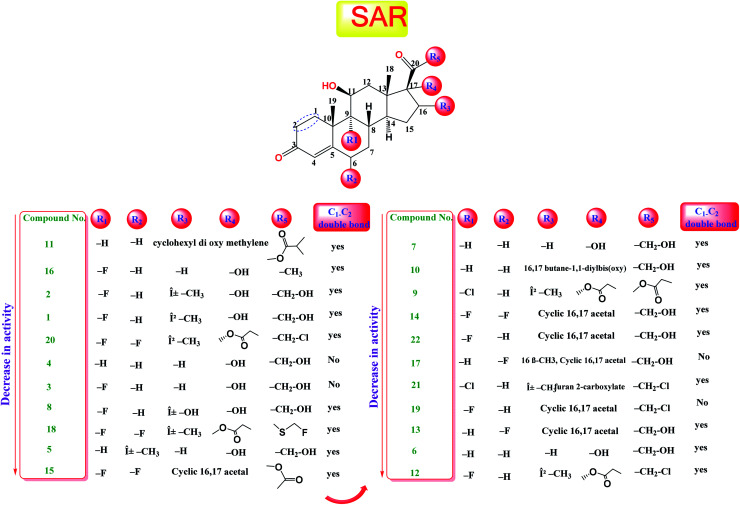
Structure–activity relationship (SAR) analysis for the studied 22 glucocorticoids as promising SARS-CoV-2 main protease inhibitors.

The cyclization at C_16_ and C_17_ of the steroidal nucleus with a cyclohexyl methylene dioxy moiety and C_20_ substitution with an isobutyrate ester with an extra double bond between C_1_ and C_2_ (compound 11) showed the most favorable activity against the SARS-CoV-2 main protease. Besides, the studied SAR revealed that the addition of an α-fluoro group at C_9_ experienced better activity. Moreover, the substitution of C_16_ of the steroidal nucleus with a methyl group showed enhanced activity against the virus main protease, yet the α orientation of the methyl group was more favorable than the β one for better activity and that may be attributed to the steric hindrance with β substituents at C_17_ and the β angular methyl of C_18_. In addition, the studied SAR let us observe the favorable extra double bond added between C_1_ and C_2_ and its effect for enhancing the activity. On the other hand, SAR revealed that substitution at C_6_ with an alkyl group had little effect on the activity against the SARS-CoV-2 main protease (Fig. 9).

## 4. Conclusion

Our study revealed the potential of repurposing glucocorticoid drugs to bind in the active site of the SARS-CoV-2 main protease. Six of the screened drugs (betamethasone 1, dexamethasone 2, fludrocortisone 3, hydrocortisone 4, triamcinolone 8, and ciclesonide 11) showed better binding through molecular docking simulation in the enzyme active site. Furthermore, the molecular dynamic simulations showed good interactions between the selected drugs and the COVID-19 main protease active site. It was noted that the drugs did not affect the structure of the protein, as the RMSD was less than 3 Å. For the MM-GBSA frontier analysis, ciclesonide 11 and betamethasone 1 were found to be the most active candidates among the other drugs. MD simulations also showed that Glu166 residue to be critical to the active site interaction and this might help in the rational designing of new molecules or modification of the current drugs. Quantum mechanics calculations were consistent with the molecular docking studies as the carbonyl group on ring A and the carboxylic group connecting isobutyryl moiety to the main nucleus of ciclesonide 11 were found to be electron-rich sites and involved in interactions with biological targets. Based on our study, the screened FDA-approved drugs—especially ciclesonide 11—could undergo preclinical and clinical trials for further evaluation of their activity against COVID-19 and to ensure their safe use. Besides, glucocorticoids may be used as lead compounds for the development of potent SARS-CoV-2 (M^pro^) inhibitors.

## Conflicts of interest

There are no conflicts to declare.

## Supplementary Material

RA-011-D0RA10674G-s001
