# Supplementary material for: Revisiting activity of some glucocorticoids as a potential inhibitor of SARS-CoV-2 main protease: theoretical study
Source: RSC Adv. 2021 Mar 9;11(17):10027–42. doi: 10.1039/d0ra10674g (PMC8695394; doi:10.1039/d0ra10674g)
Supplement: RA-011-D0RA10674G-s001 [file RA-011-D0RA10674G-s001.pdf]

Electronic Supplementary Material (ESI) for RSC Advances.
This journal is © The Royal Society of Chemistry 2021

Supplementary data

Table 1SI: The RMSD of ligands fit to protein active during 100 ns and last 50 ns trajectories 2
Figure 1: Snapshots of betamethasone contact with active site at 0, 50, 100ns.....................................3
Figure 2: The histogram of Betamethasone-6LU7 contact over the course of the trajectory.....................4
Figure 3: The ligand property trajectory of the Betamethasone-6LU7 complex during the 100 ns simulation 4
Figure 4: Betamethasone - 6LU7 Interaction shown by the active site amino acids in each trajectory frame, white refers to zero interaction while the deep color indicated more interactions........................5
Figure 5: Snapshots of dexamethasone contact with active site at 0, 50, 100 ns. 6
Figure 6 The histogram of Dexamethasone-6LU7 contact over the course of the trajectory. 7
Figure 7: The ligand property trajectory of the dexamethasone-6LU7 complex during the 100 ns simulation 7
Figure 8: Dexamethasone - 6LU7 Interaction shown by the active site amino acids in each trajectory frame, white refers to zero interaction while the deep color indicated more interactions........................8
Figure 9: Snapshots of hydrocortisone contact with active site at 0, 50, 100ns..................................9
Figure 10: The histogram of hydrocortisone-6LU7 contact over the course of the trajectory..................10
Figure 11: The ligand property trajectory of the hydrocortisone-6LU7 complex during the 100 ns simulation 10
Figure 12: Hydrocortisone - 6LU7 Interaction shown by the active site amino acids in each trajectory frame, white refers to zero interaction while the deep color indicated more interactions......................11
Figure 13: Snapshots of triamcinolone contact with active site at 0, 50, 100ns.................................12
Figure 14: The histogram of triamcinolone-6LU7 contact over the course of the trajectory....................13
Figure 15: The ligand property trajectory of the triamcinolone-6LU7 complex during the 100 ns simulation 13
Figure 16: Triamcinolone - 6LU7 Interaction shown by the active site amino acids in each trajectory frame, white refers to zero interaction while the deep color indicated more interactions......................14
Figure 17: Snapshots of fludrocortisone contact with active site at 0, 50, 100ns. 15
Figure 18: The histogram of fludrocortisone-6LU7 contact over the course of the trajectory. 16
Figure 19: The ligand property trajectory of the fludrocortisone-6LU7 complex during the 100 ns simulation. 16
Figure 20: Fludrocortisone - 6LU7 Interaction shown by the active site amino acids in each trajectory frame, white refers to zero interaction while the deep color indicated more interactions......................17
Figure 21: Snapshots of ciclesonide contact with active site at 0, 50, 100ns. 18
Figure 22: The histogram of ciclesonide-6LU7 contact over the course of the trajectory. 19
Figure 23: The ligand property trajectory of the ciclesonide-6LU7 complex during the 100 ns simulation 19
Figure 24: 3 D docking representations of Glucocorticosteroids and N3 inhibitor against the binding site of the Covid-19 main protease 20
Figure 25: A) Surface of Mpro showing the positioning and fitting of the selected compounds, B) surface and maps of the selected compounds compared to N3 inhibitor 29
Figure 26: 2 D diagram (a) and 3 D representation (b) of the superimposition of the co-crystallized (red) and the docking pose (green) of N3 in the Covid-19 main protease binding site with RMSD of 1.23 Å 33

Table 1SI: The RMSD of ligands fit to protein active during 100 ns and last 50 ns trajectories

**Ligand**	**100 ns Å**	**Last 50 ns Å**
Betamethasone	3.42	3.74
Dexamethasone	3.70	3.75
Hydrocortisone	6.97	7.70
Triamcinolone	4.45	4.43
Fludrocortisone	5.25	5.10
Ciclesonide	3.88	4.68

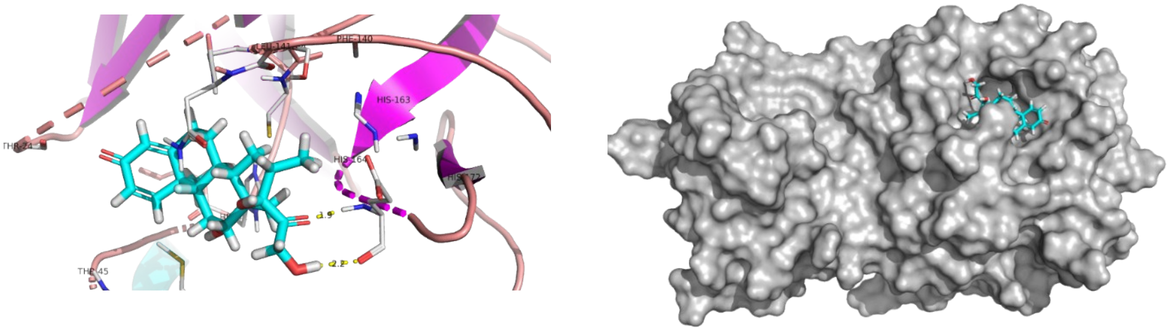

0 ns

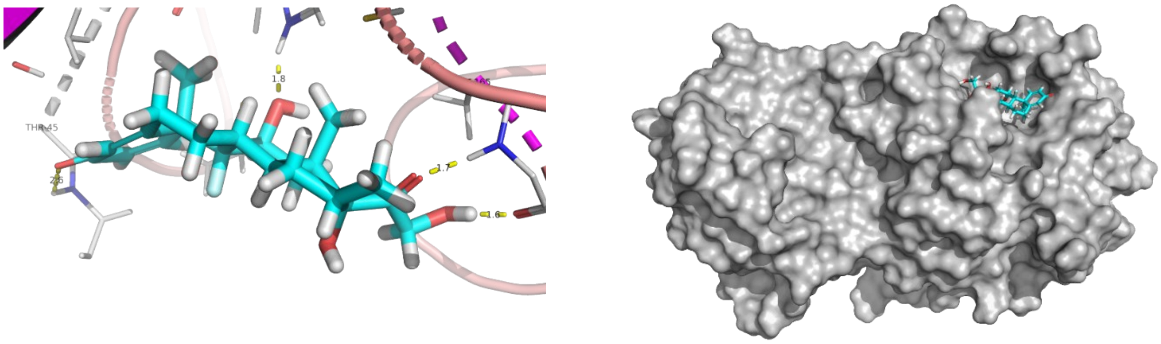

50 ns

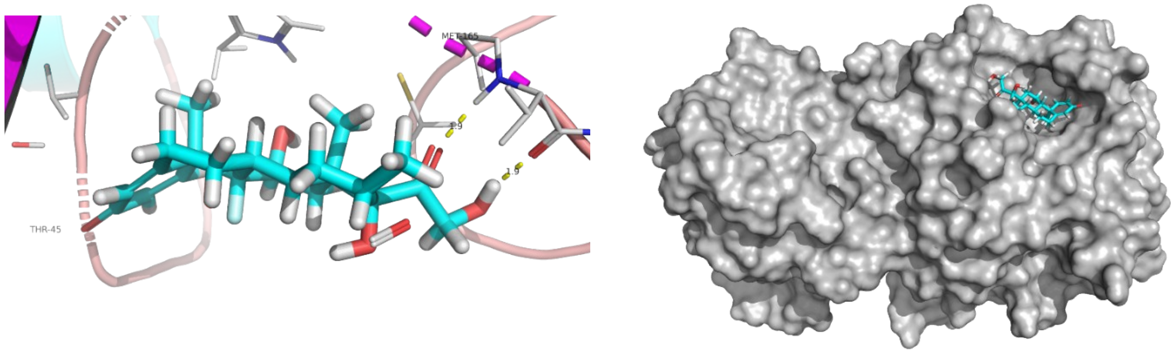

100 ns

Figure 1: Snapshots of betamethasone contact with active site at 0, 50, 100ns.

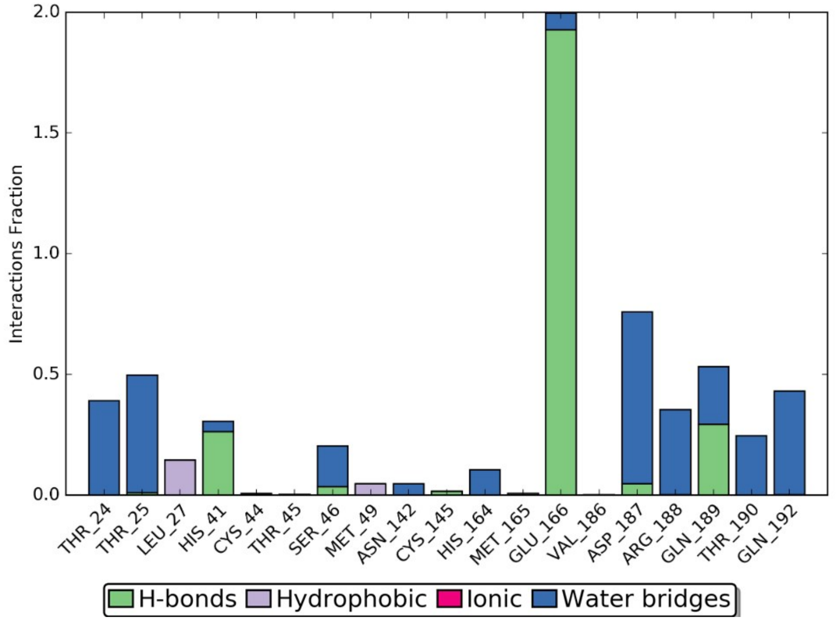

Figure 2: The histogram of Betamethasone-6LU7 contact over the course of the trajectory.

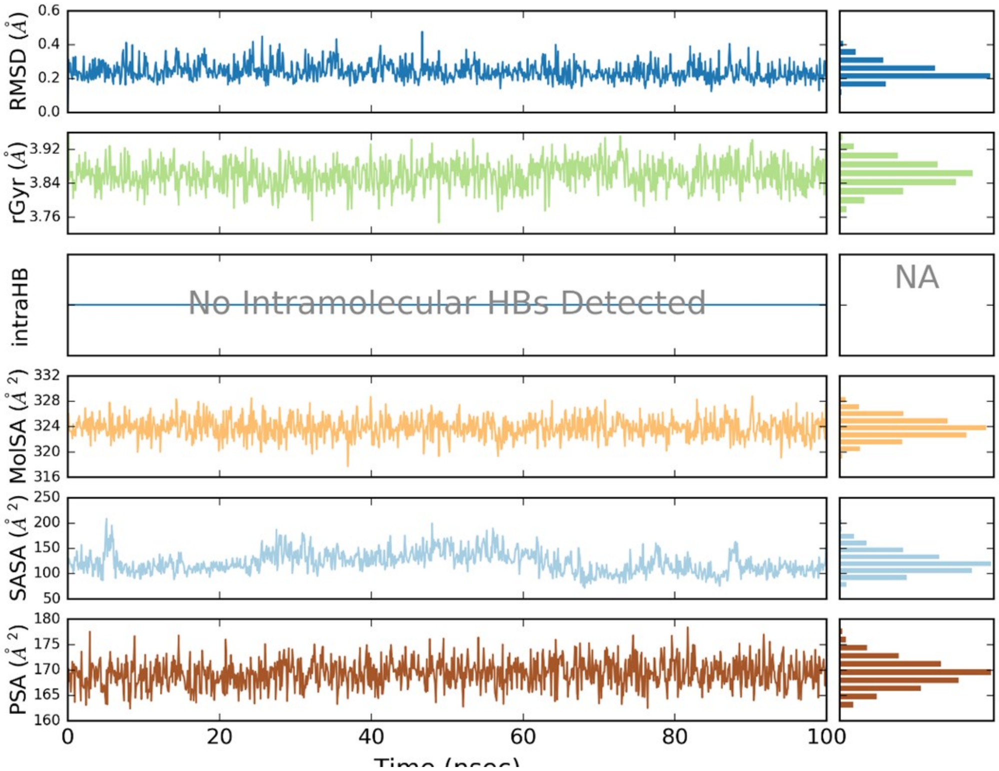

Figure 3: The ligand property trajectory of the Betamethasone-6LU7 complex during the 100 ns simulation

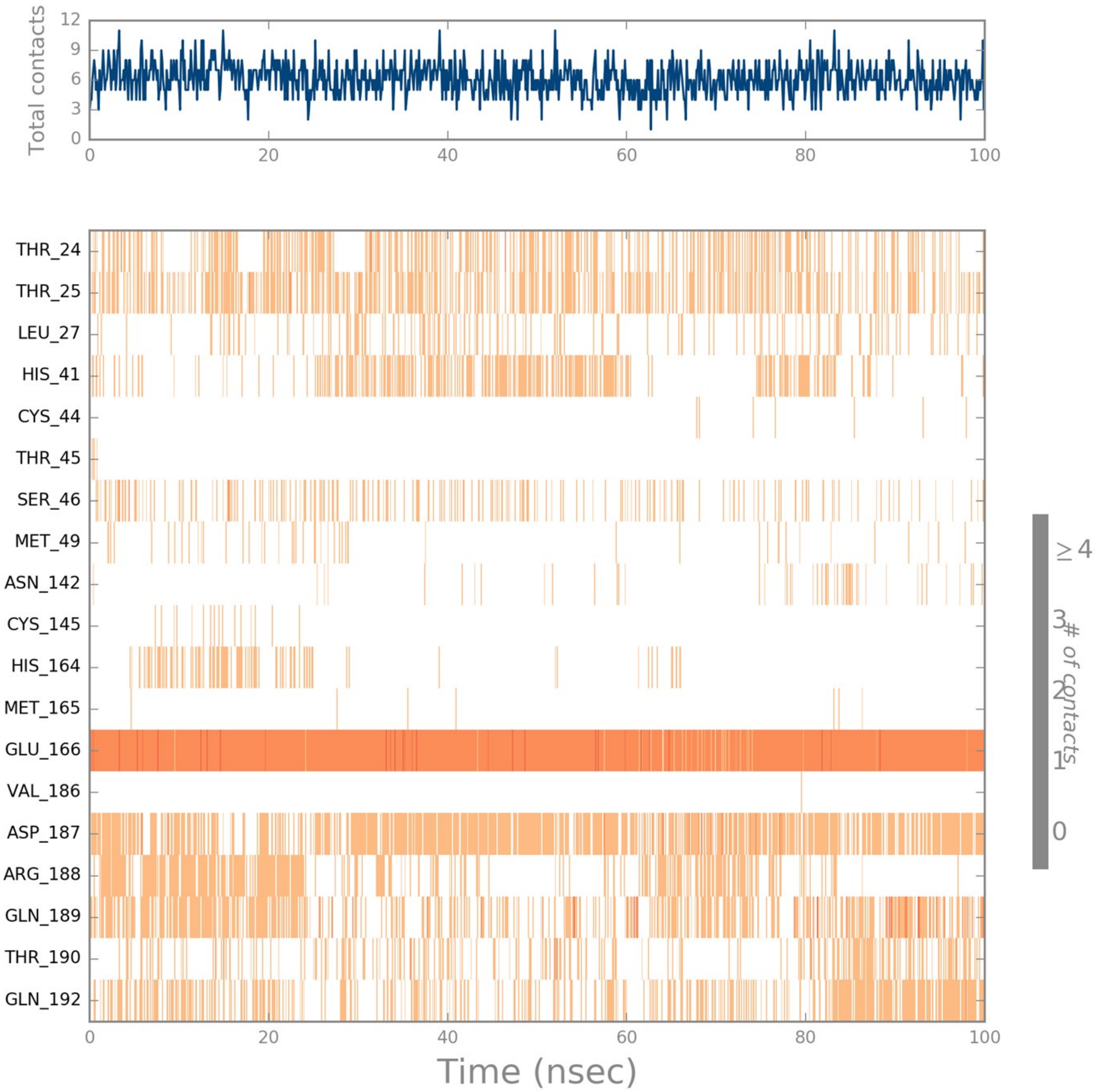

Figure 4: Betamethasone - 6LU7 Interaction shown by the active site amino acids in each trajectory frame, white refers to zero interaction while the deep color indicated more interactions.

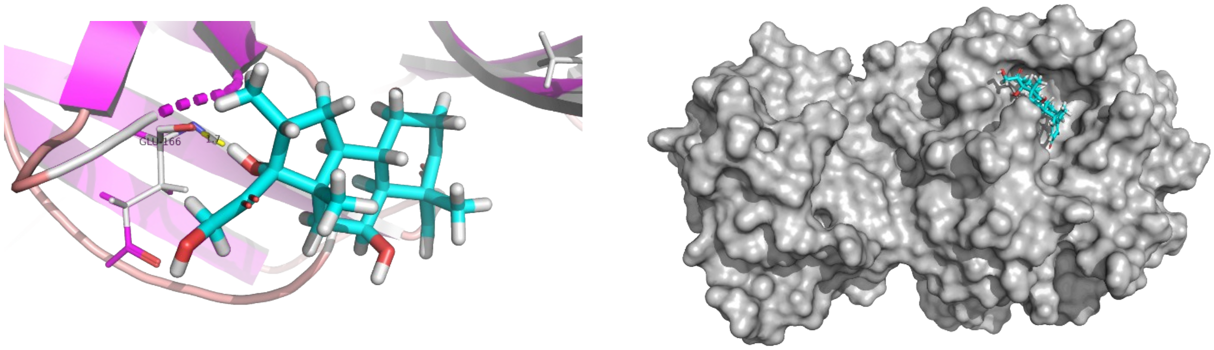

0 ns

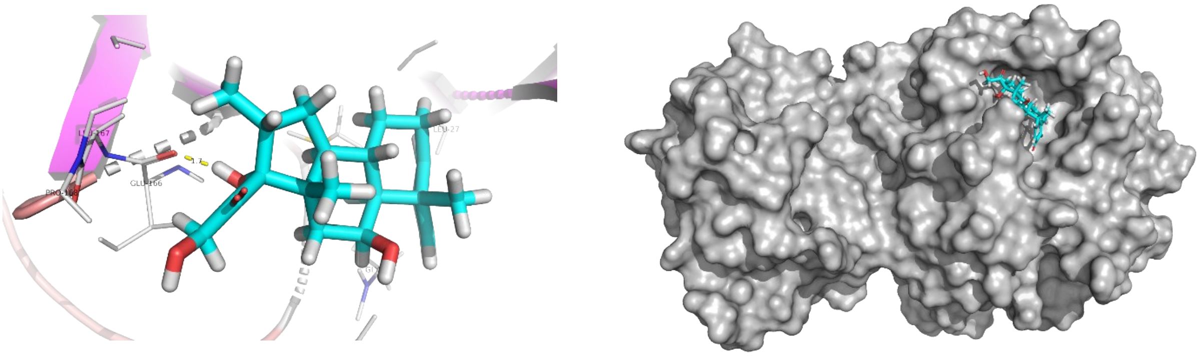

50 ns

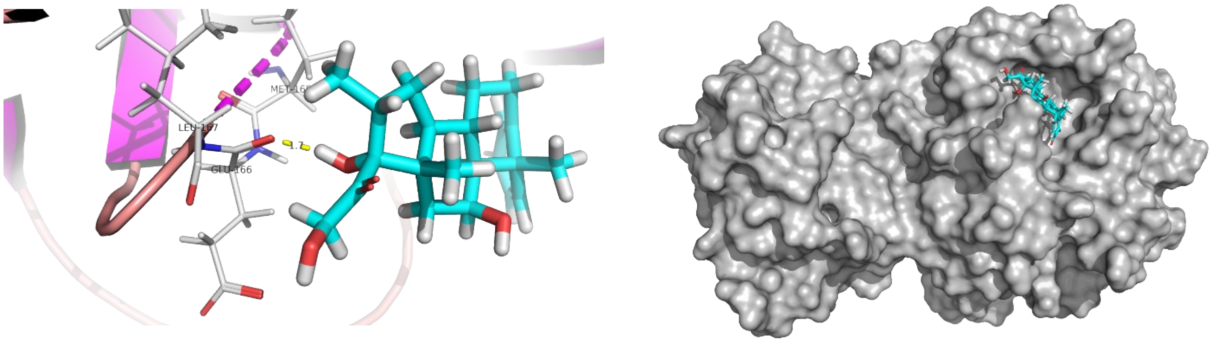

100 ns

Figure 5: Snapshots of dexamethasone contact with active site at 0, 50, 100 ns.

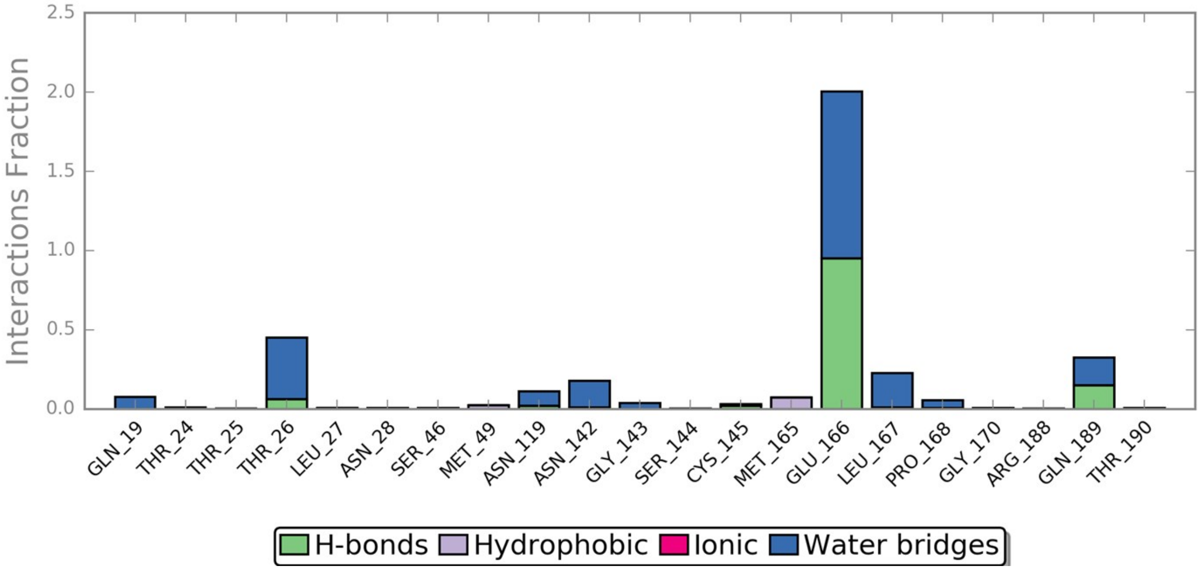

Figure 6 The histogram of Dexamethasone-6LU7 contact over the course of the trajectory.

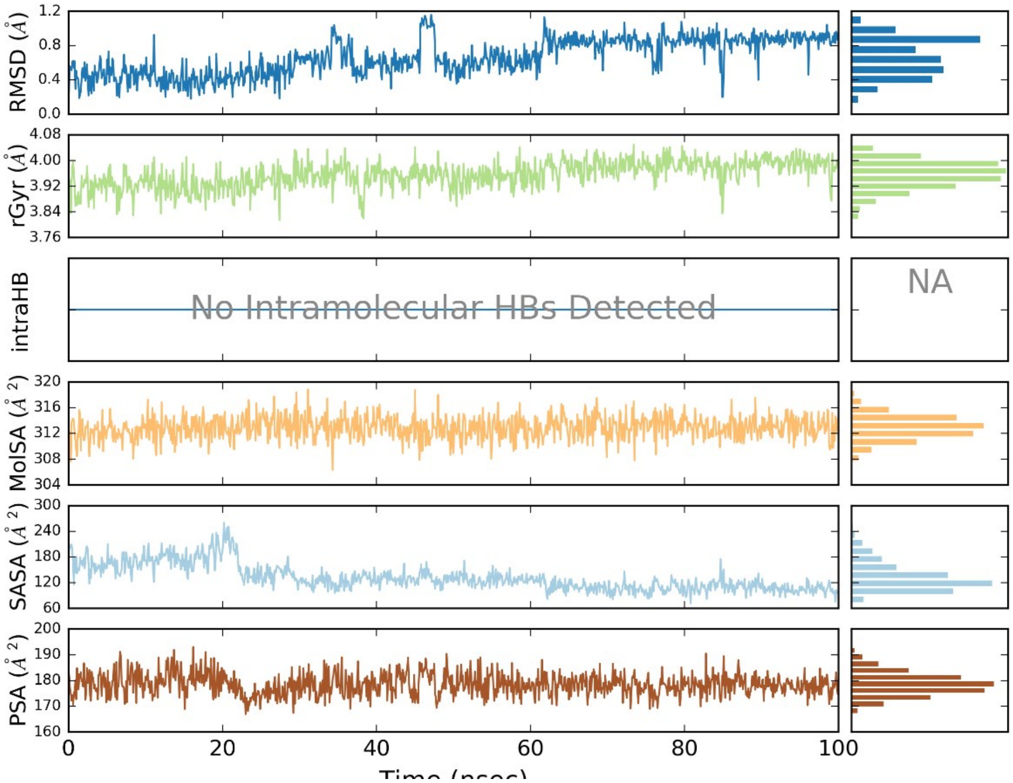

Figure 7: The ligand property trajectory of the dexamethasone-6LU7 complex during the 100 ns simulation

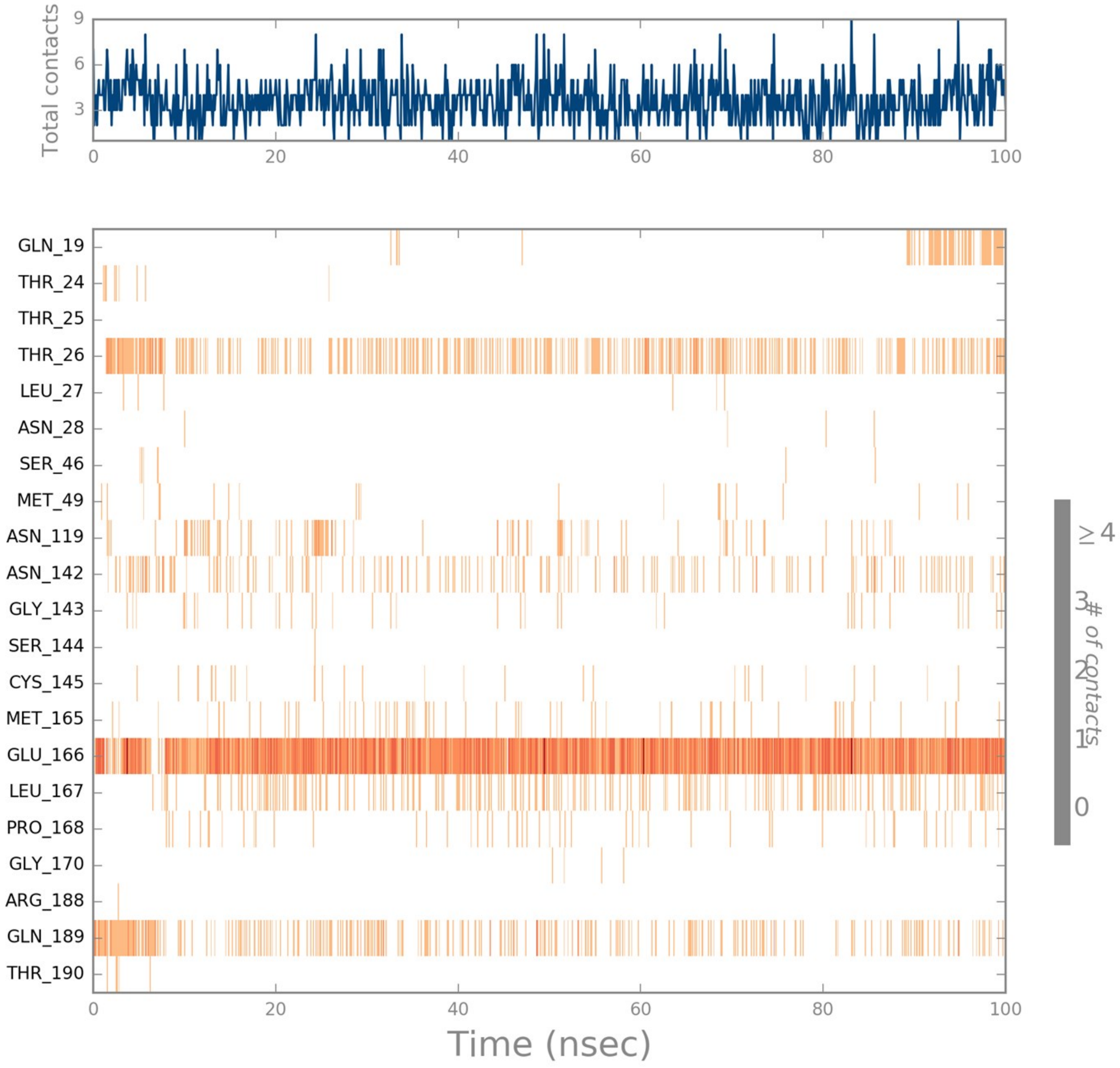

Figure 8: Dexamethasone - 6LU7 Interaction shown by the active site amino acids in each trajectory frame, white refers to zero interaction while the deep color indicated more interactions.

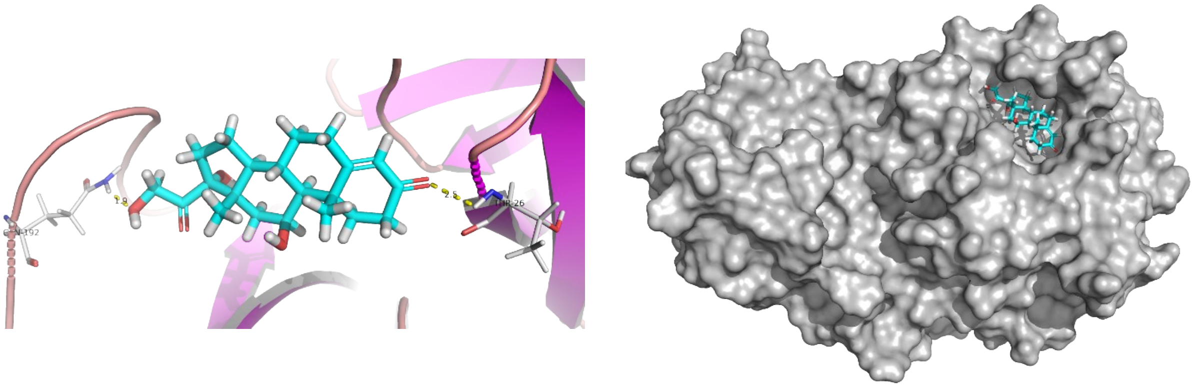

0 ns

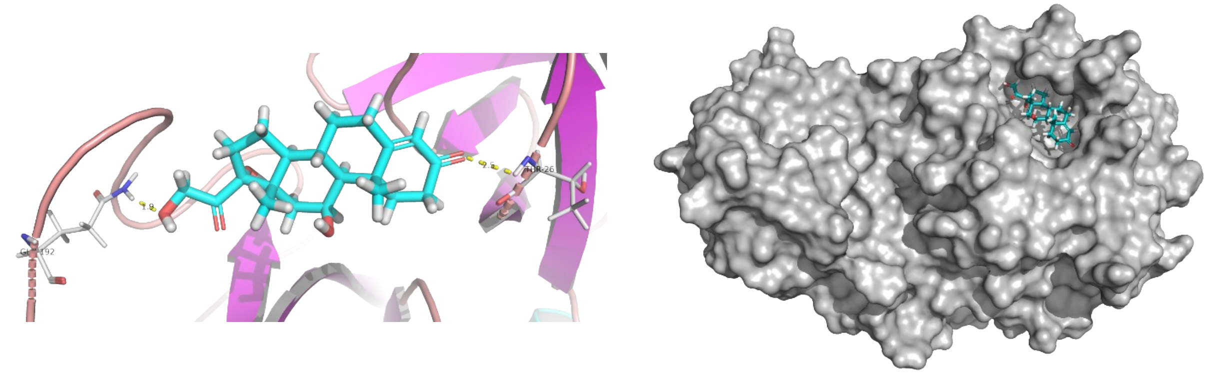

50 ns

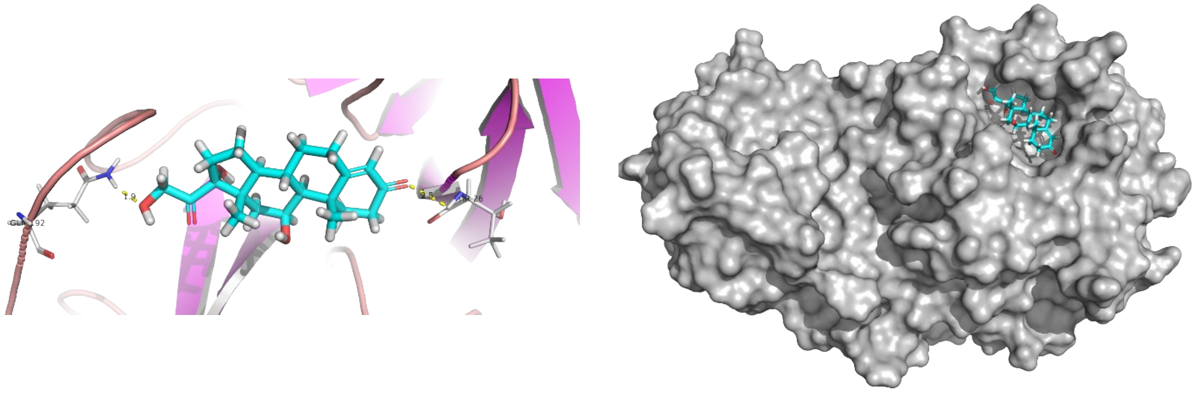

100 ns

Figure 9: Snapshots of hydrocortisone contact with active site at 0, 50, 100ns.

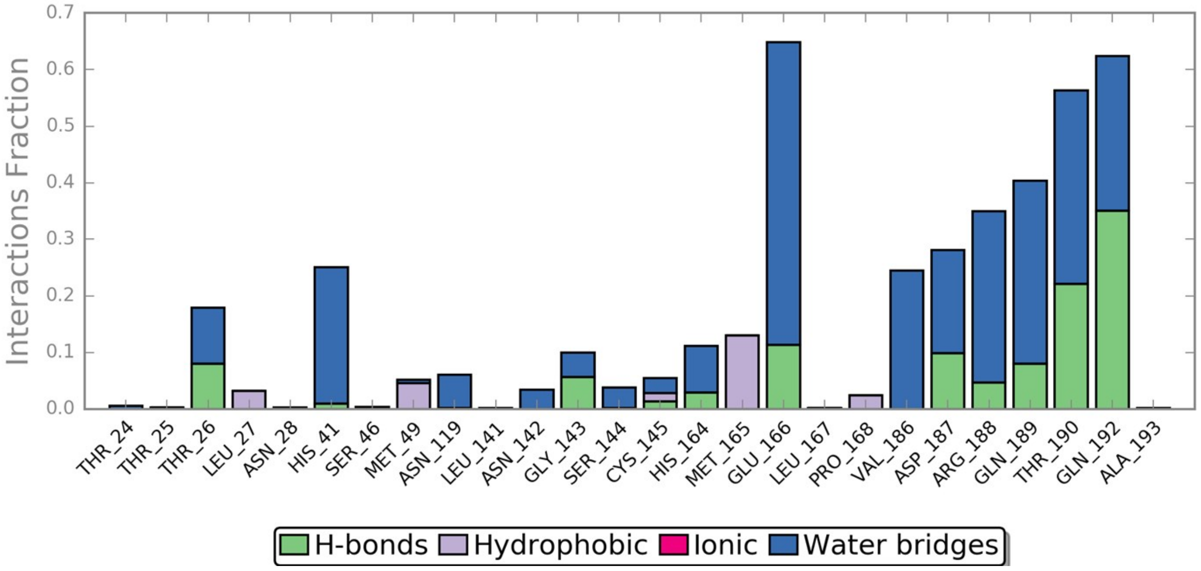

Figure 10: The histogram of hydrocortisone-6LU7 contact over the course of the trajectory.

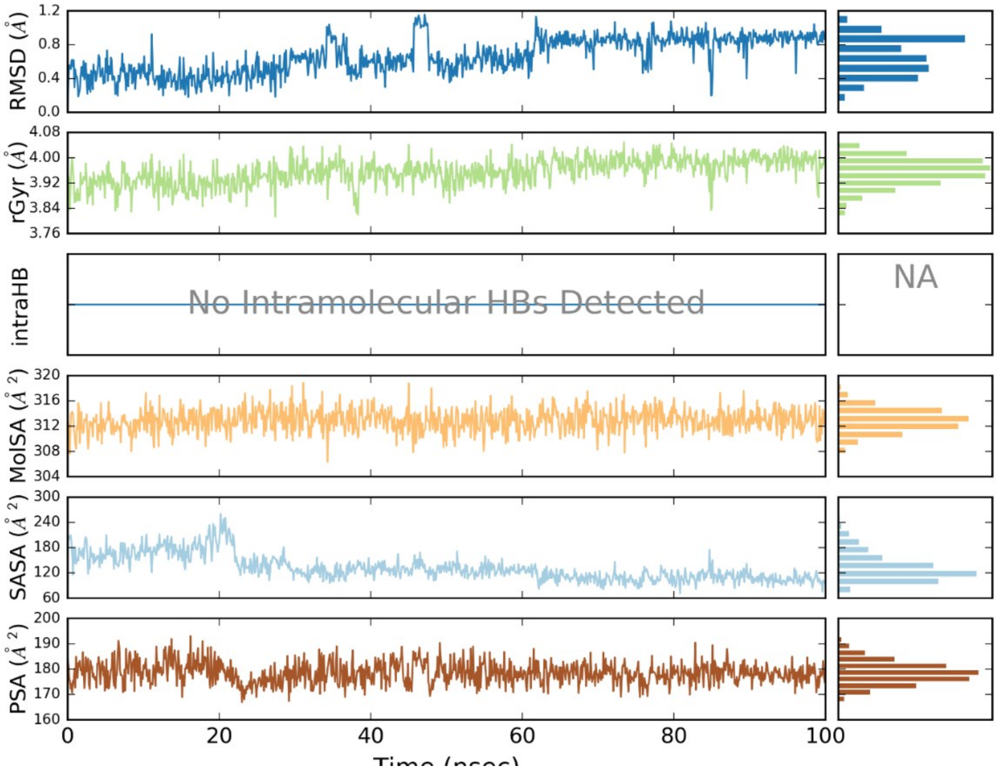

Figure 11: The ligand property trajectory of the hydrocortisone-6LU7 complex during the 100 ns simulation

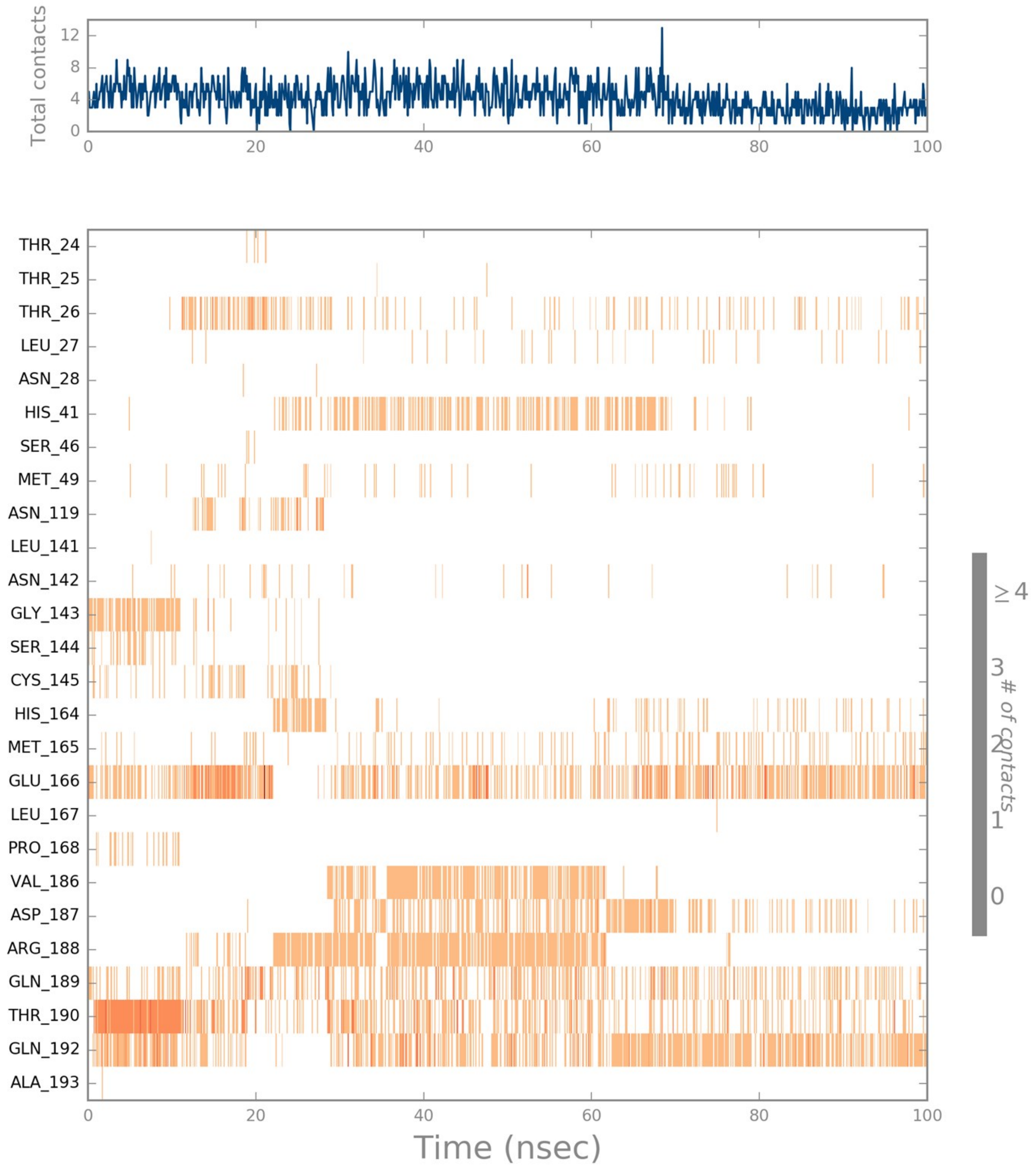

Figure 12: Hydrocortisone - 6LU7 Interaction shown by the active site amino acids in each trajectory frame, white refers to zero interaction while the deep color indicated more interactions.

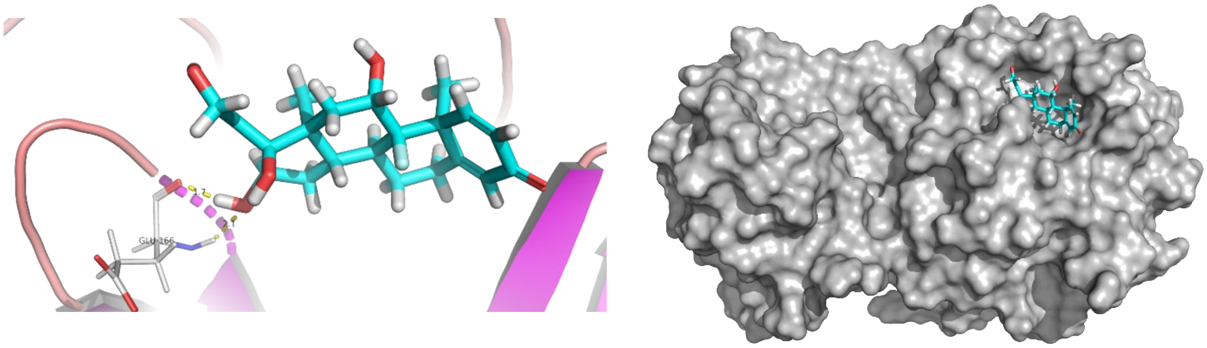

0ns

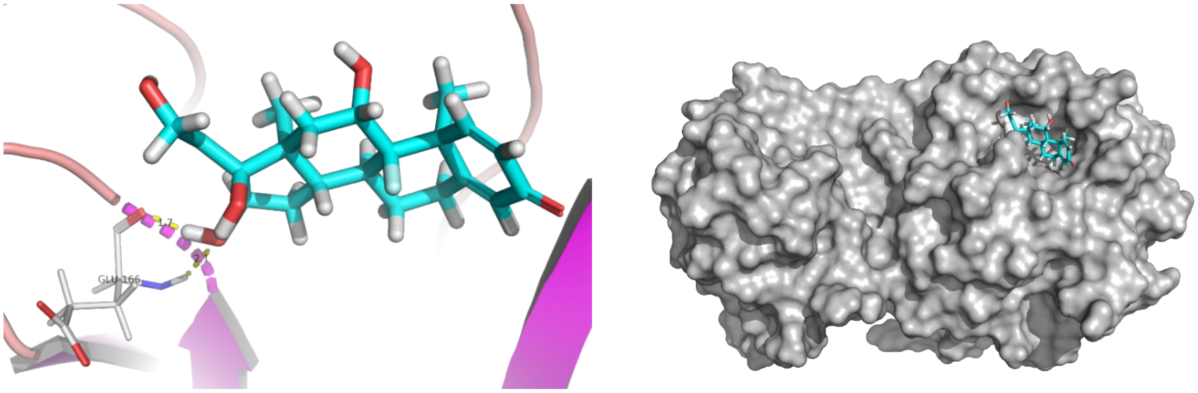

50ns

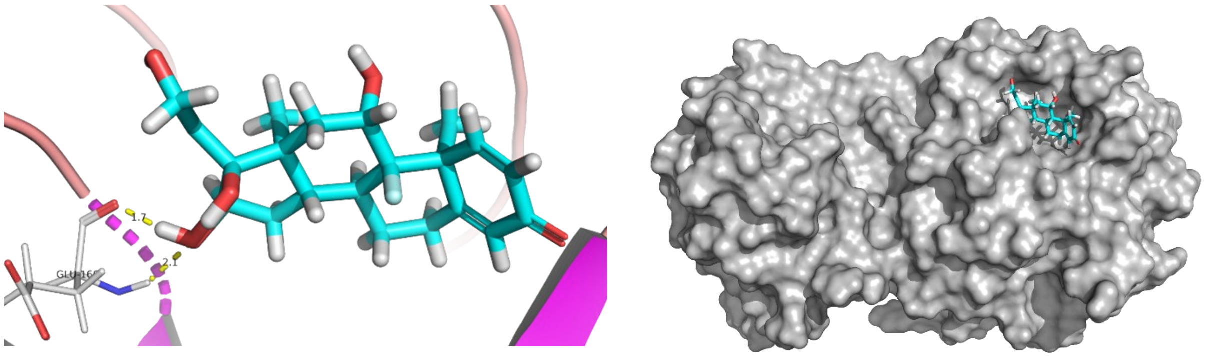

100ns

Figure 13: Snapshots of triamcinolone contact with active site at 0, 50, 100ns.

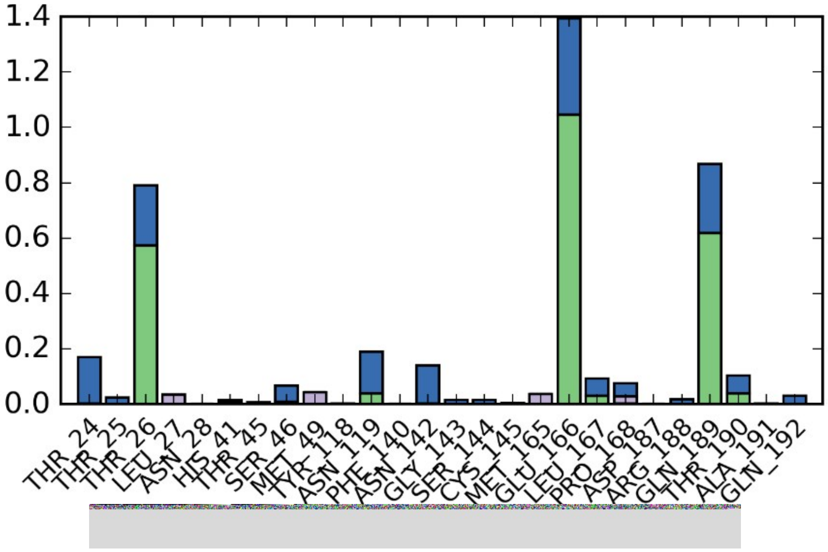

Figure 14: The histogram of triamcinolone-6LU7 contact over the course of the trajectory.

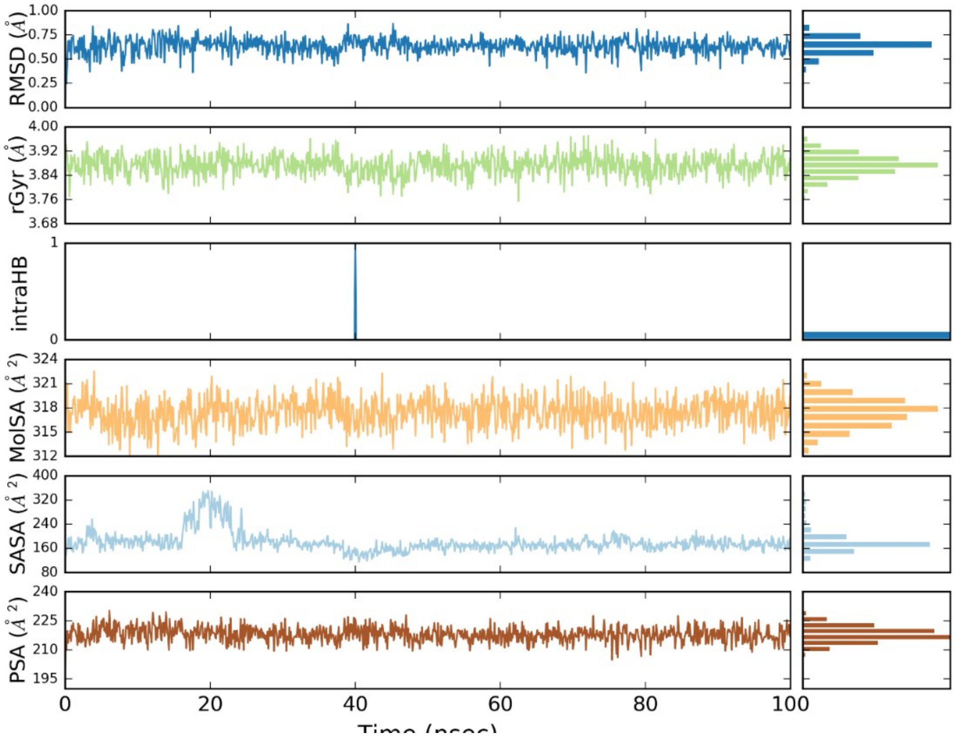

Figure 15: The ligand property trajectory of the triamcinolone-6LU7 complex during the 100 ns simulation

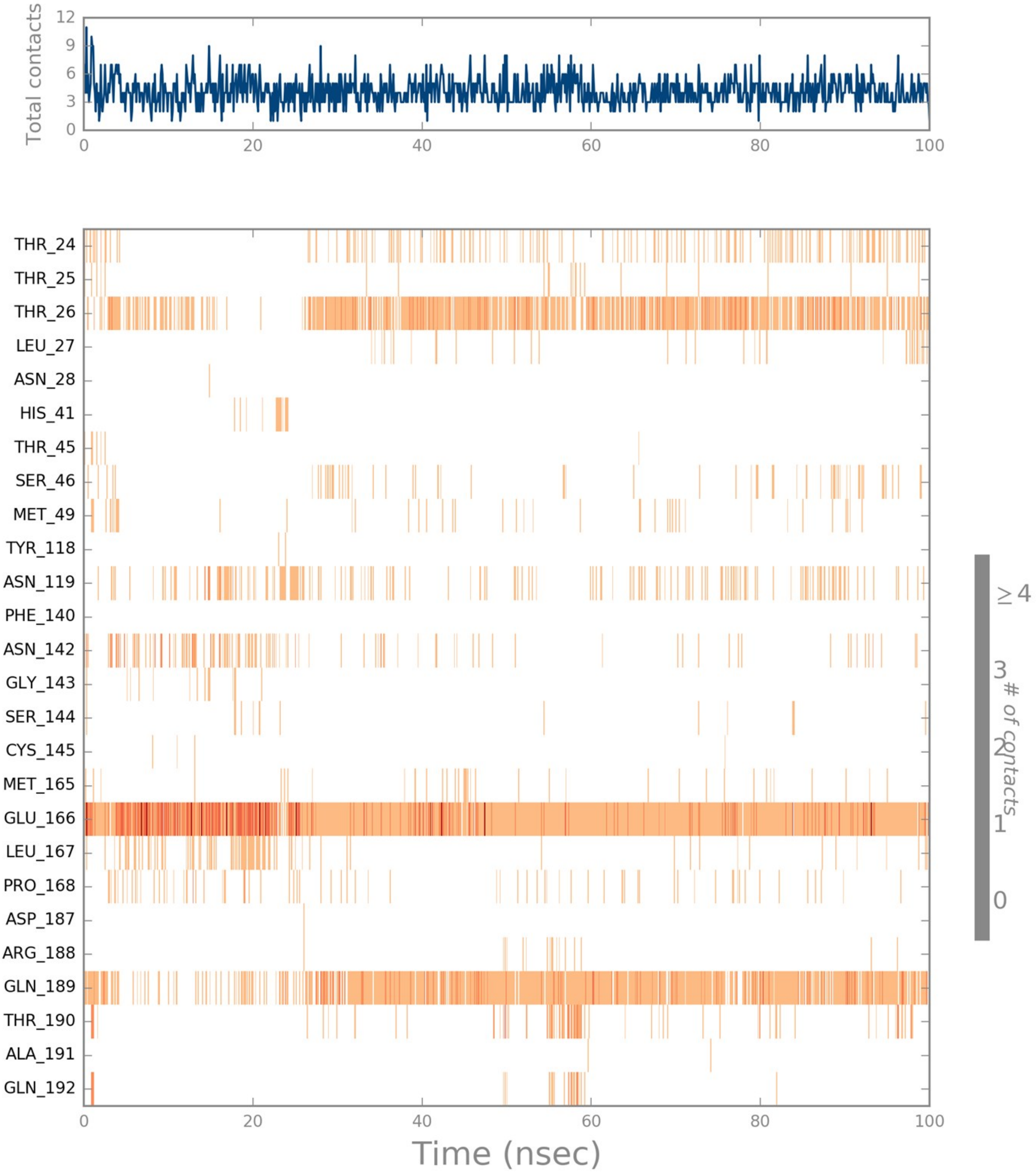

Figure 16: Triamcinolone - 6LU7 Interaction shown by the active site amino acids in each trajectory frame, white refers to zero interaction while the deep color indicated more interactions.

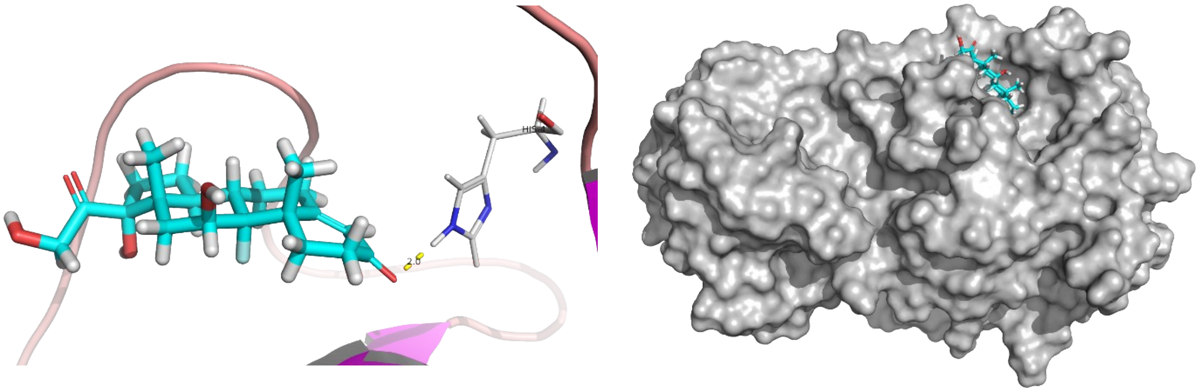

0ns

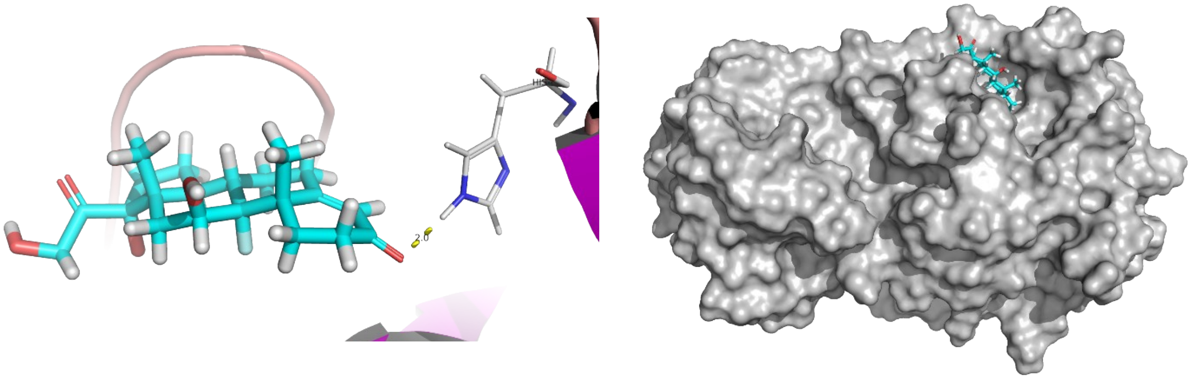

50ns

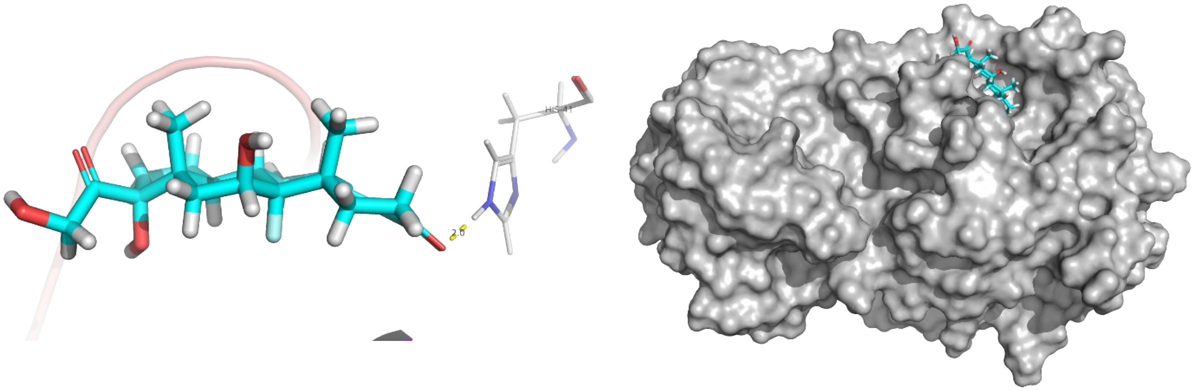

100 ns

Figure 17: Snapshots of fludrocortisone contact with active site at 0, 50, 100ns.

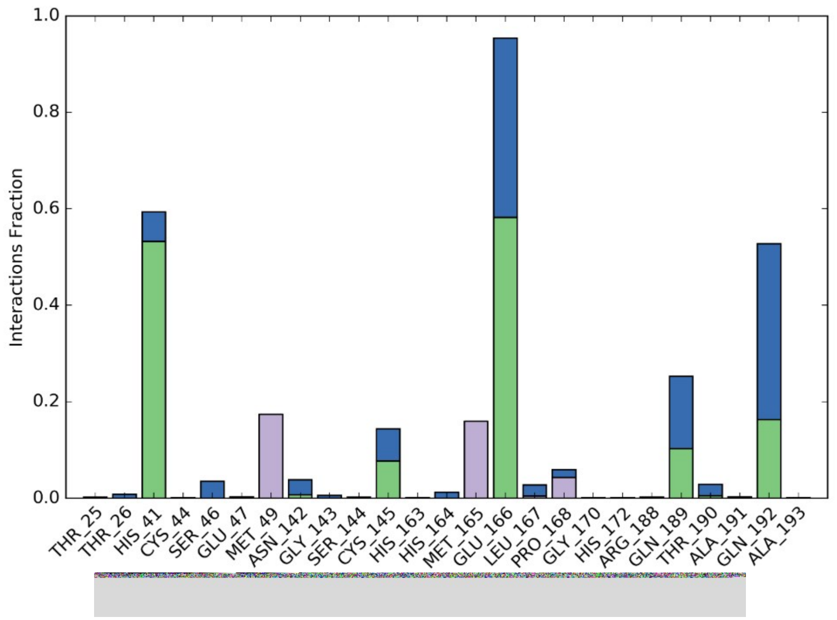

Figure 18: The histogram of fludrocortisone-6LU7 contact over the course of the trajectory.

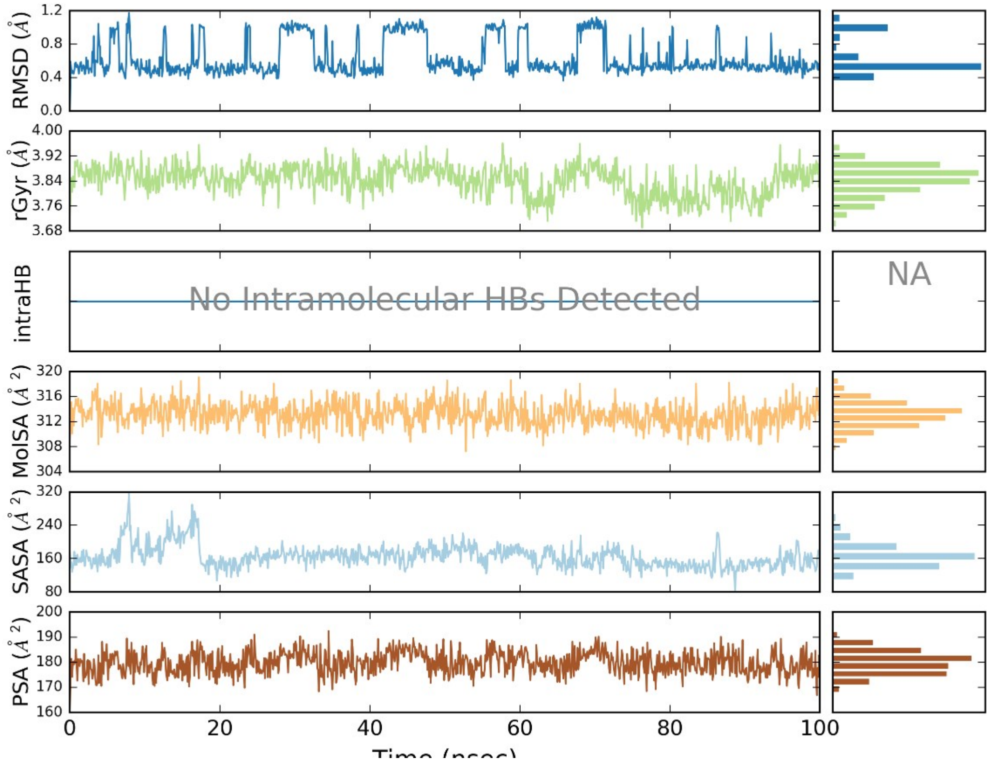

Figure 19: The ligand property trajectory of the fludrocortisone-6LU7 complex during the 100 ns simulation.

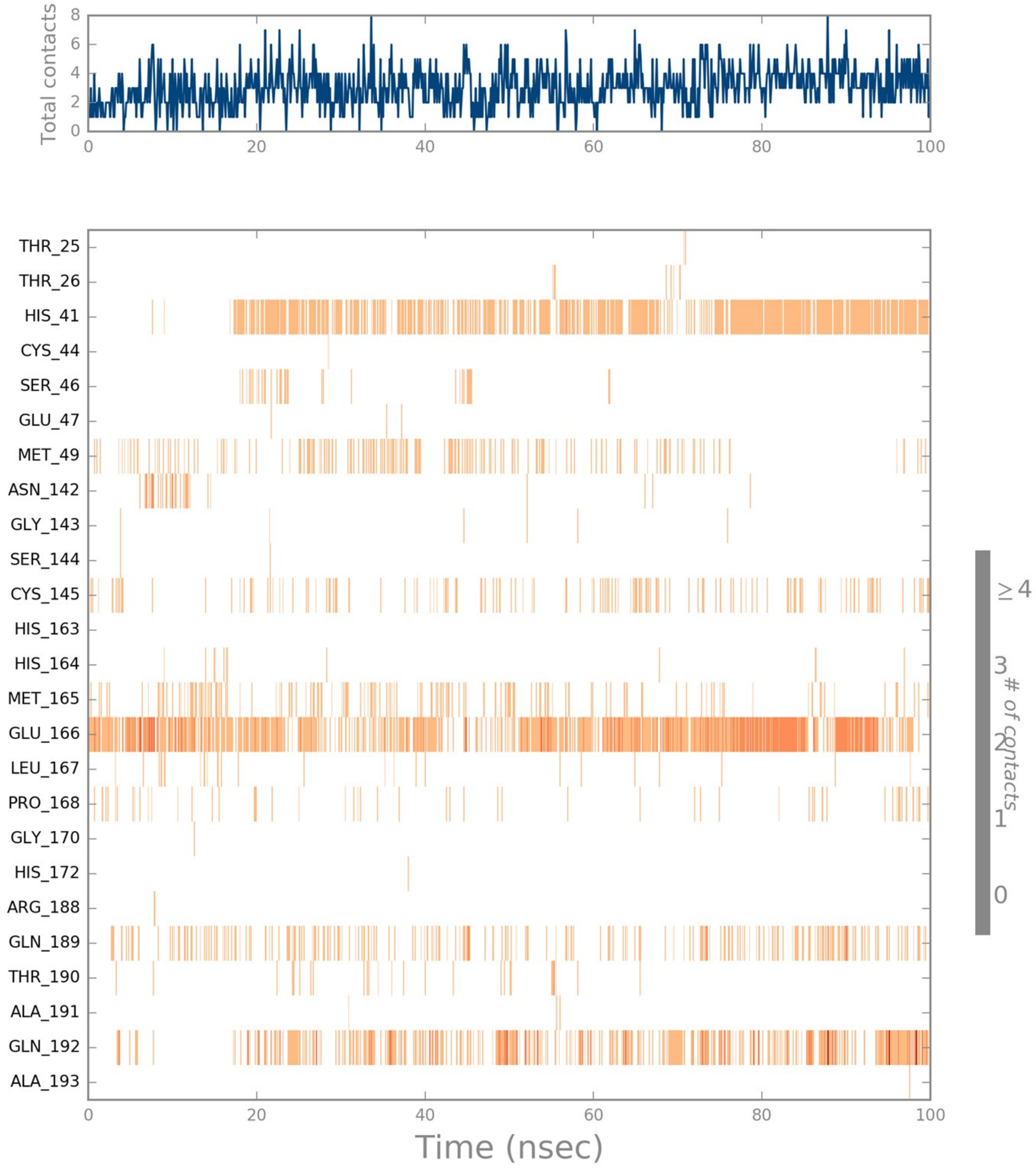

Figure 20: Fludrocortisone - 6LU7 Interaction shown by the active site amino acids in each trajectory frame, white refers to zero interaction while the deep color indicated more interactions.

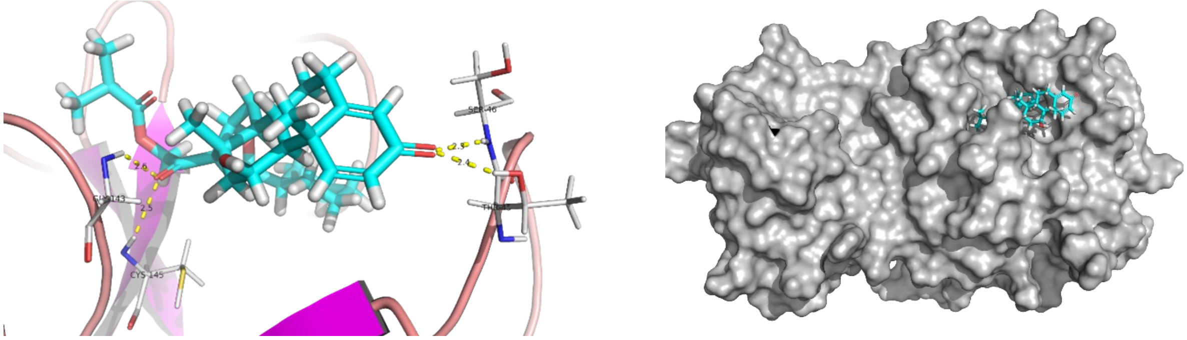

0 ns

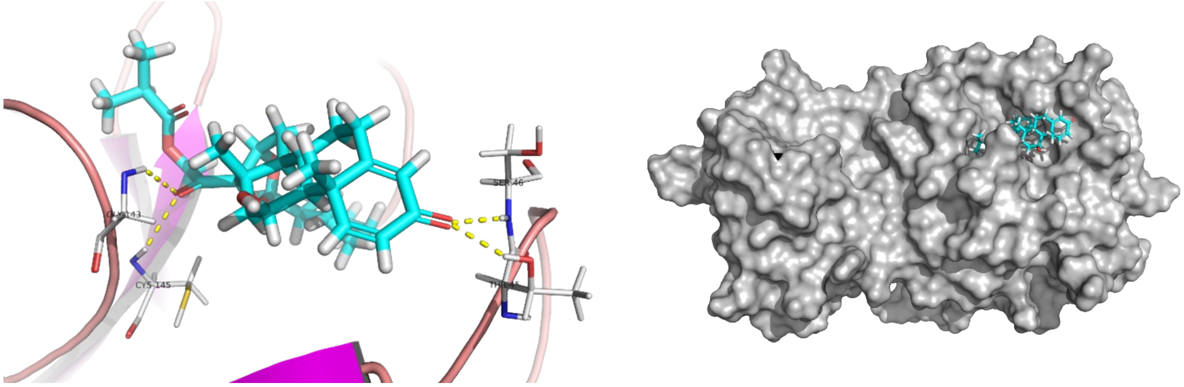

50 ns

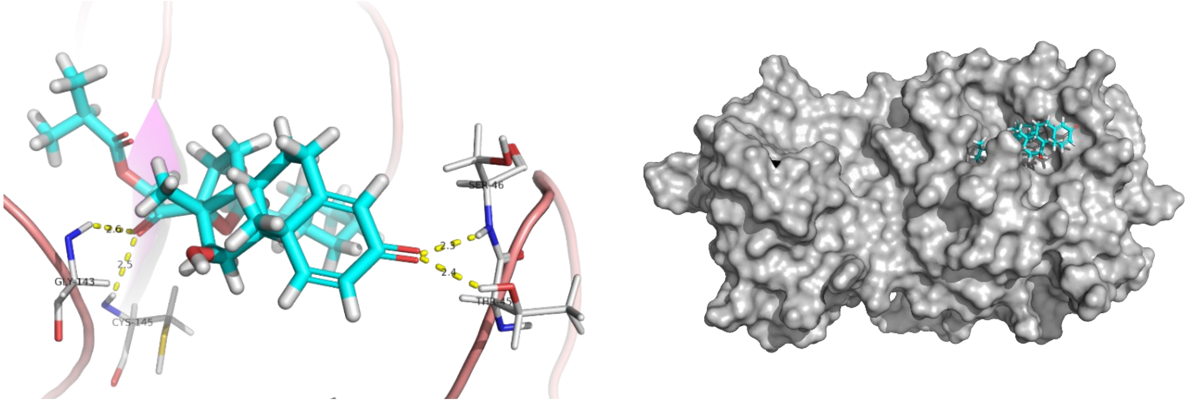

100 ns

Figure 21: Snapshots of ciclesonide contact with active site at 0, 50, 100ns.

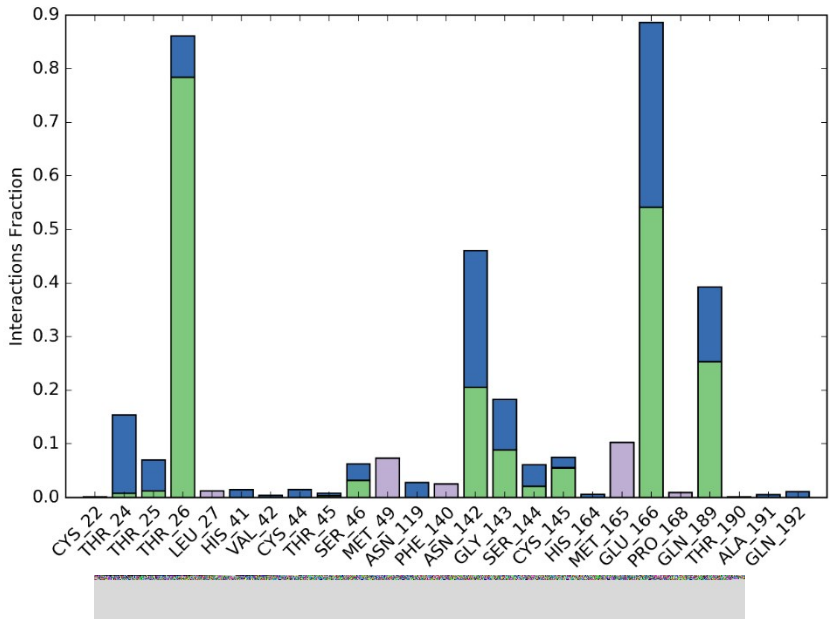

Figure 22: The histogram of ciclesonide-6LU7 contact over the course of the trajectory.

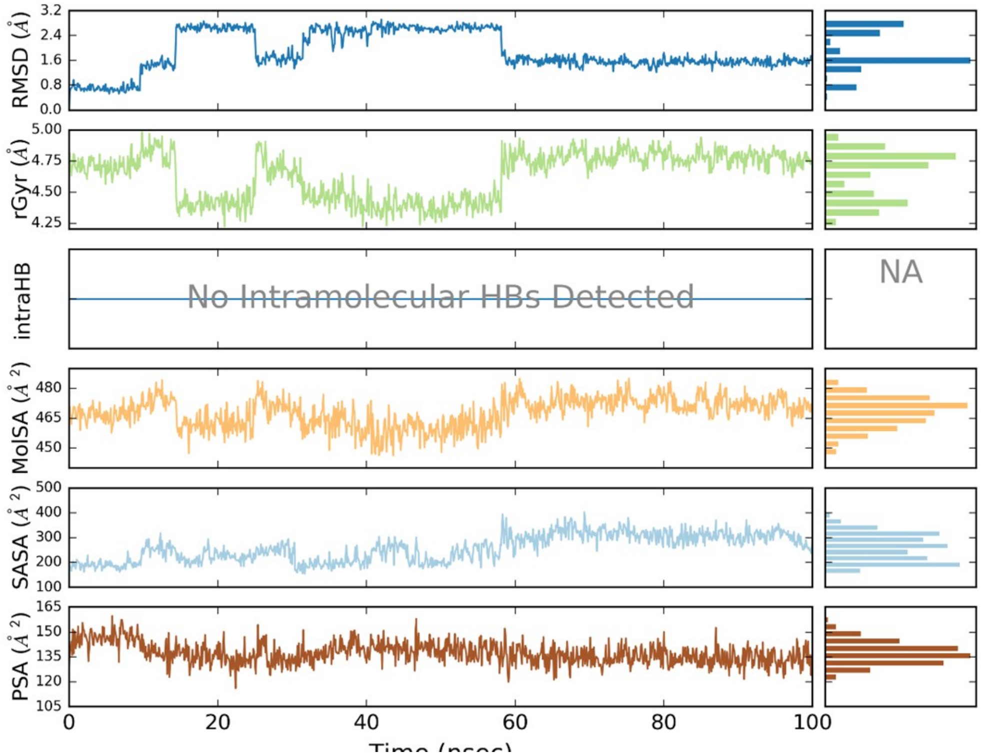

Figure 23: The ligand property trajectory of the ciclesonide-6LU7 complex during the 100 ns simulation

Figure 24: 3 D docking representations of Glucocorticosteroids and N3 inhibitor against the binding site of the Covid-19 main protease.

No.	Glucocorticosteroids	3D
1	Betamethasone	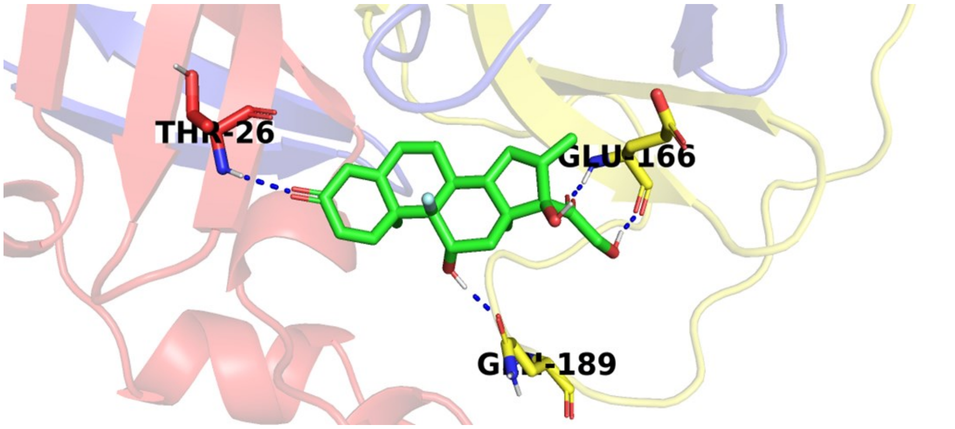
2	Dexamethasone	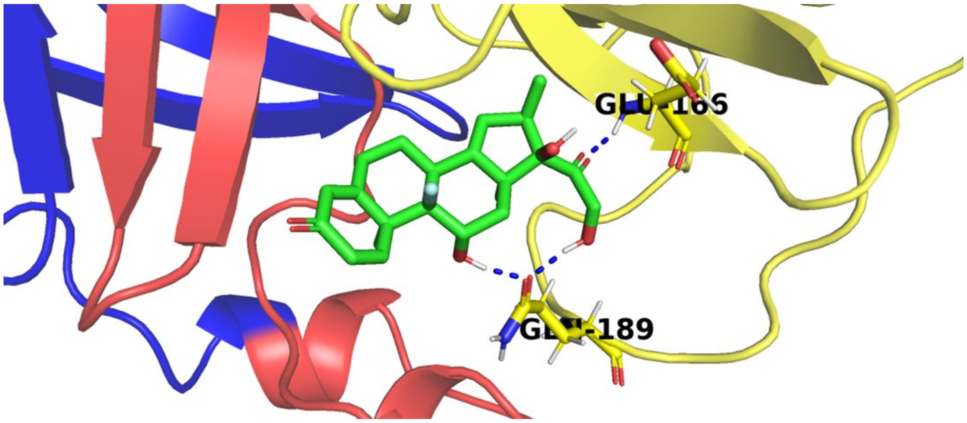
3	Fludrocortisone	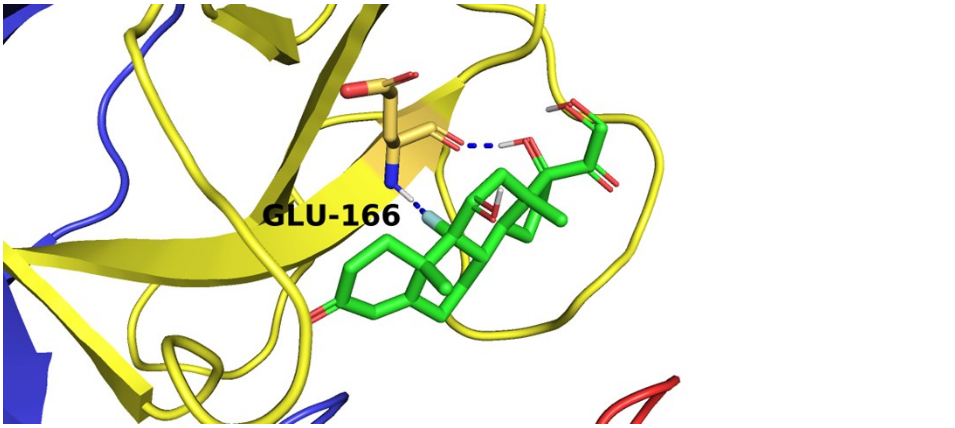

**4**	**Hydrocortisone**	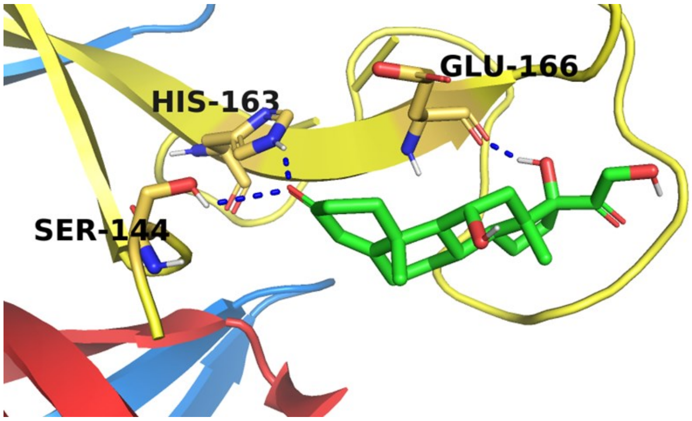
**5**	**Methylprednisolone**	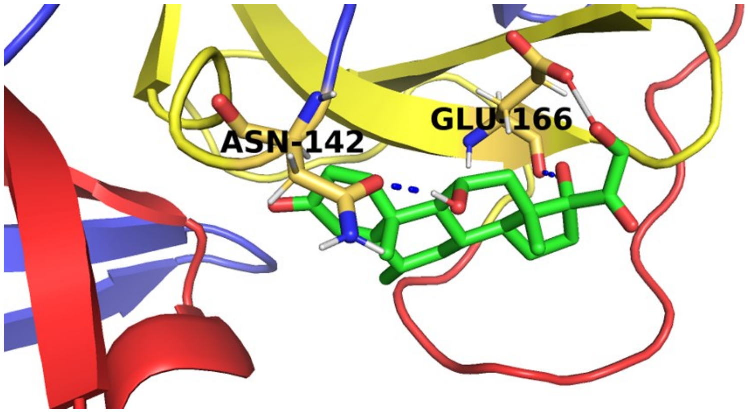
**6**	**Prednisone**	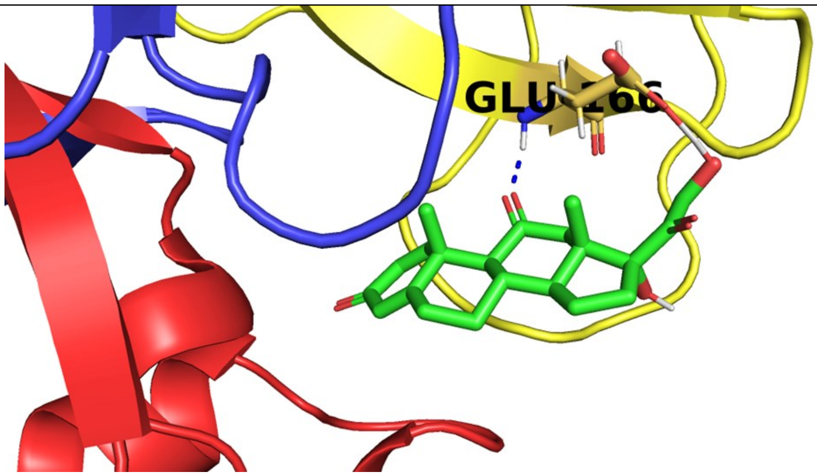

**7**	**prednisolone**	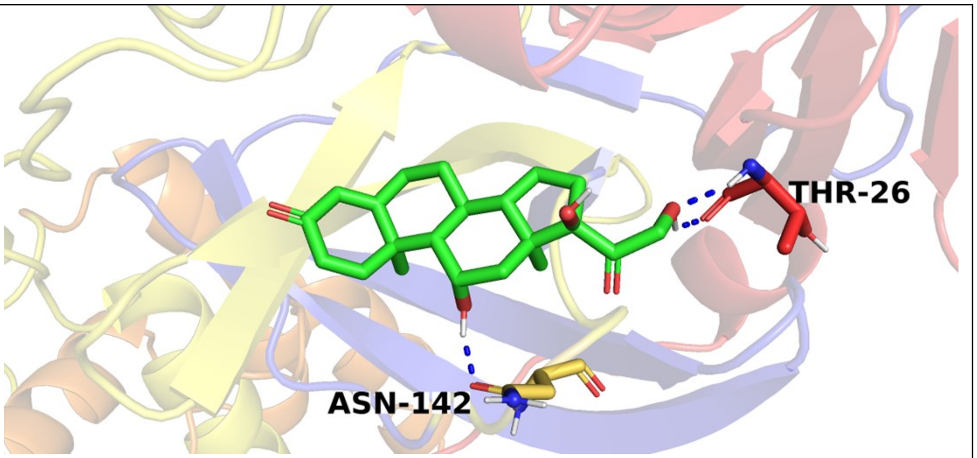
**8**	**Triamcinolone**	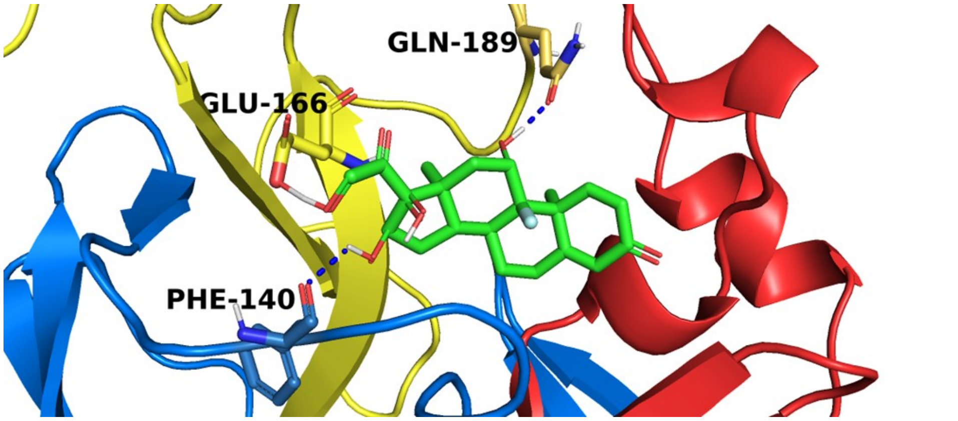
**9**	**Beclomethasone dipropionate**	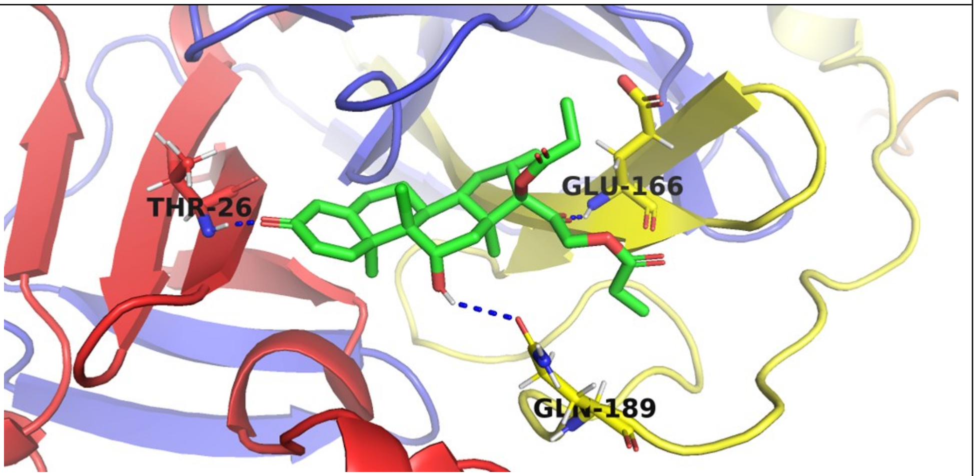

**10**	**Budesonide**	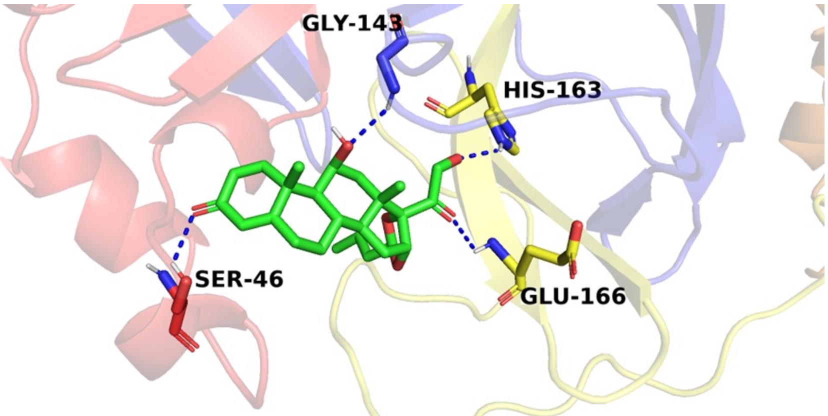
**11**	**Ciclesonide**	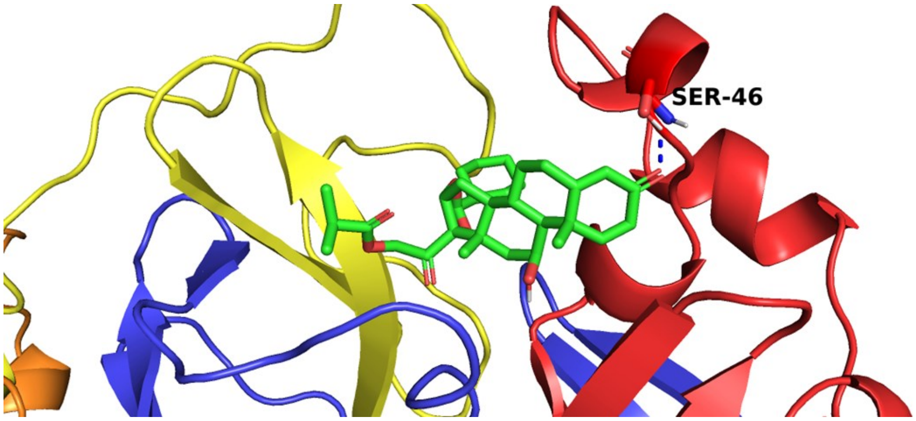
**12**	**Clobetasol propionate**	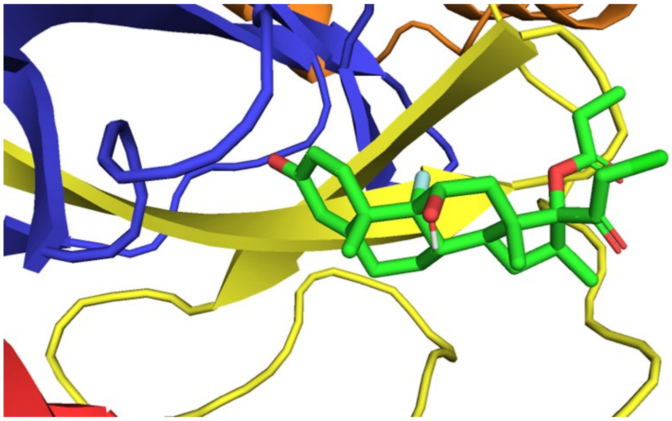

13	**Flunisolide**	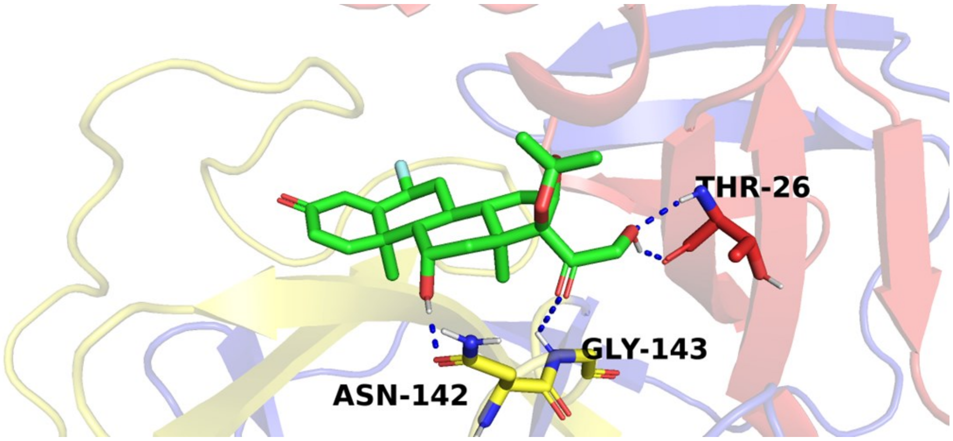
14	**Fluocinolone acetonide**	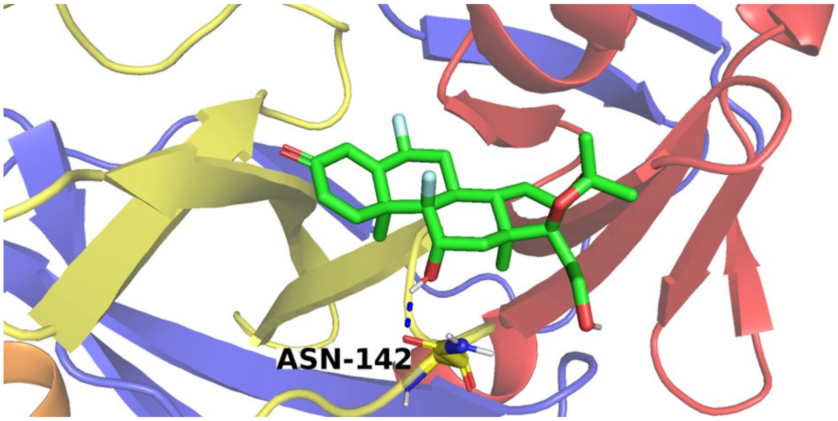
15	**Fluocinonide**	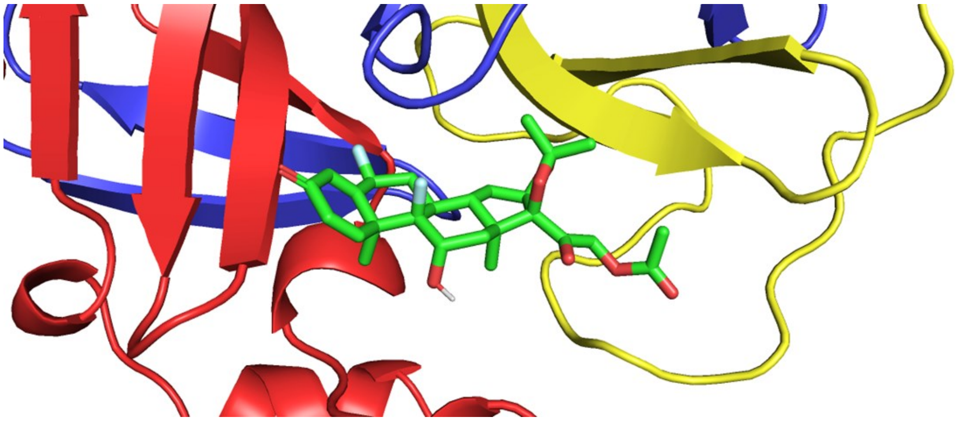

16	**Fluorometholone**	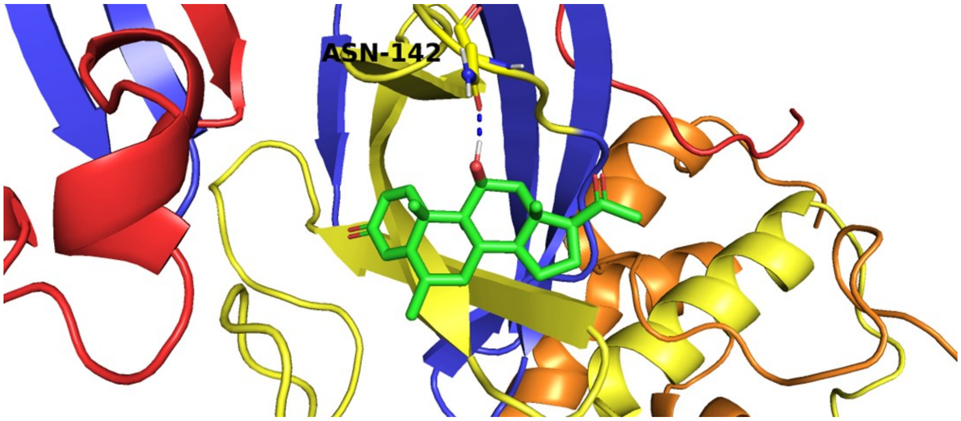
17	**Flurandrenolide**	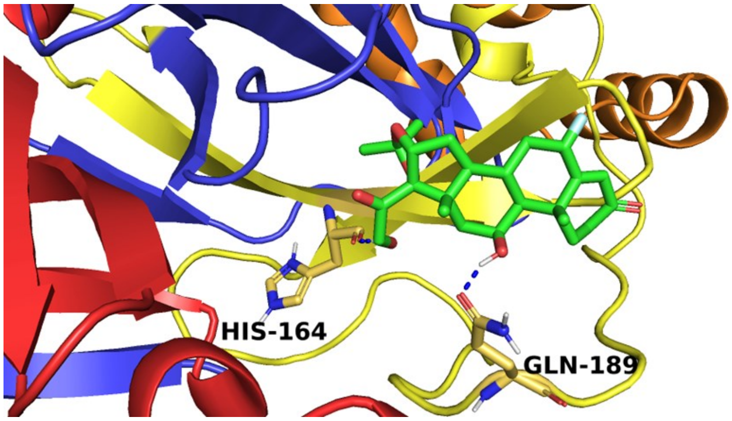
18	**Fluticasone propionate**	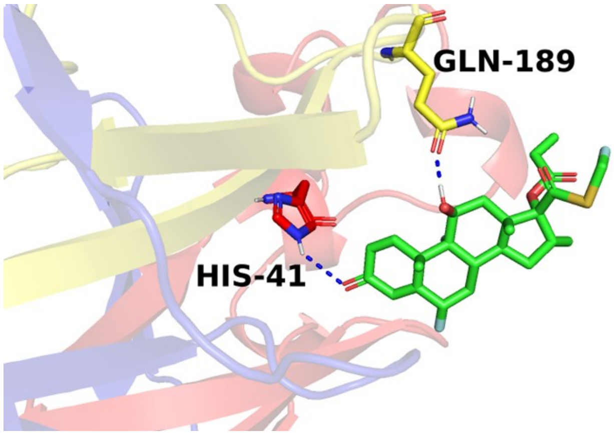

19	**Halcinonide**	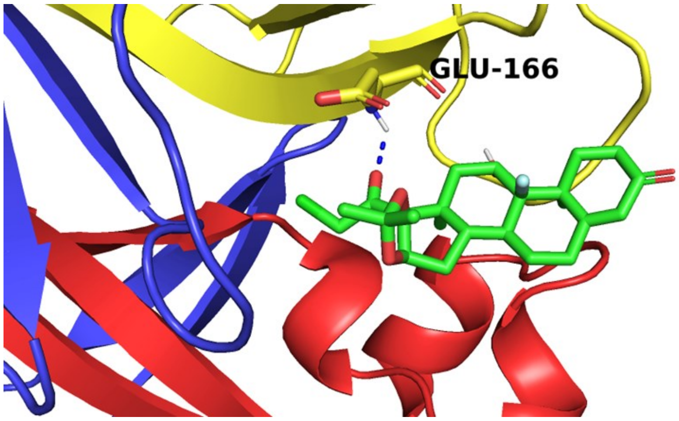
20	**Halobetasol propionate**	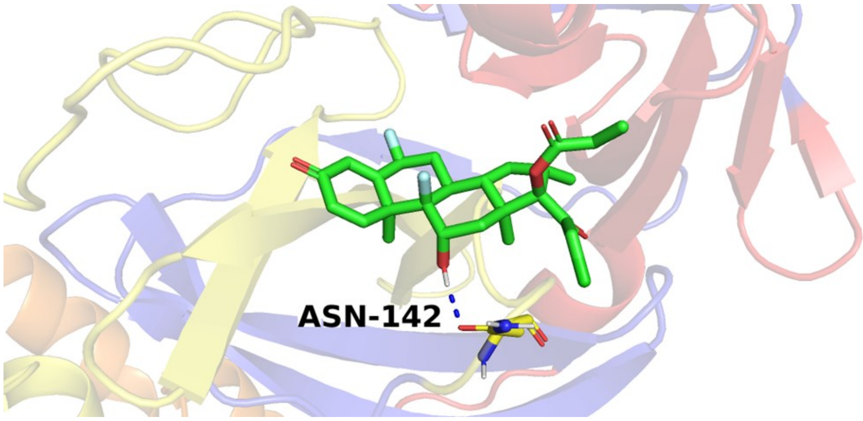
21	**Mometasone furoate**	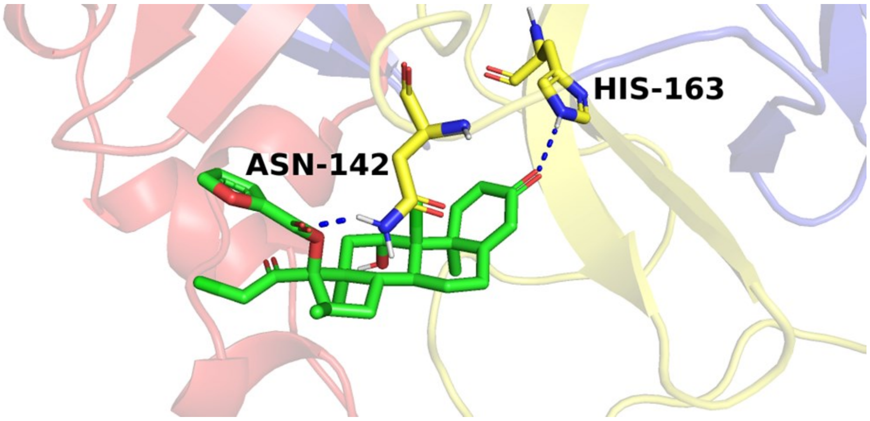

**22**	**Triamcinolone acetonide**	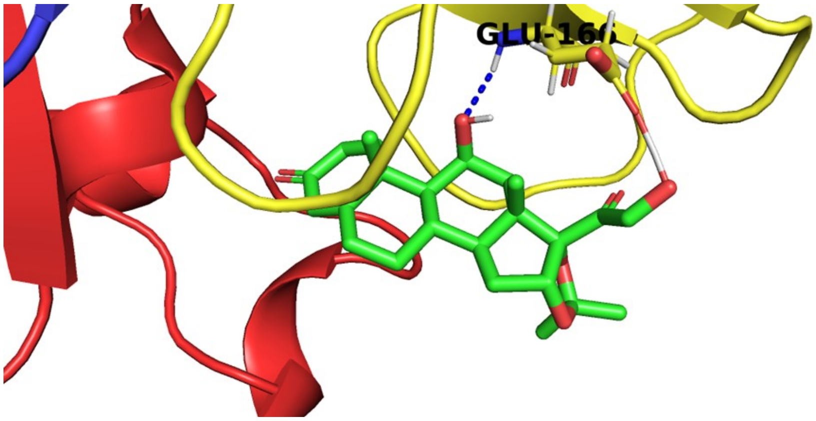
**23**	**N3**	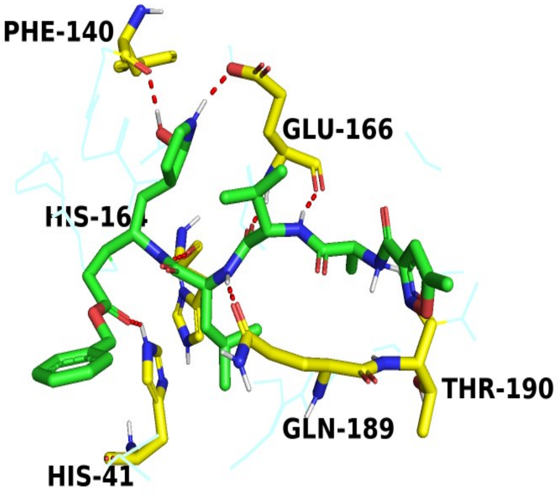

Figure 25: A) Surface of M^{pro} showing the positioning and fitting of the selected compounds, B) surface and maps of the selected compounds compared to N3 inhibitor.

No.	Glucocorticosteroids	A	B
1	Betamethasone	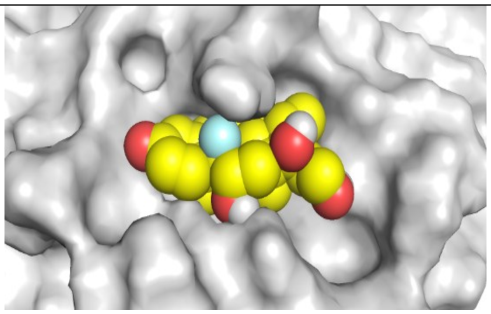	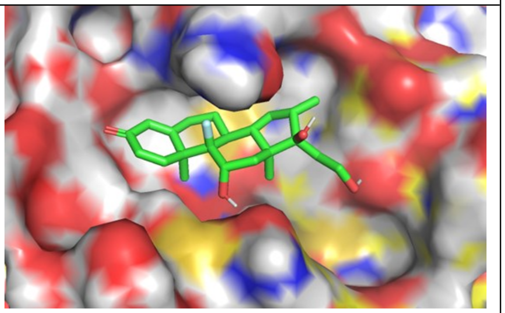
2	Dexamethasone	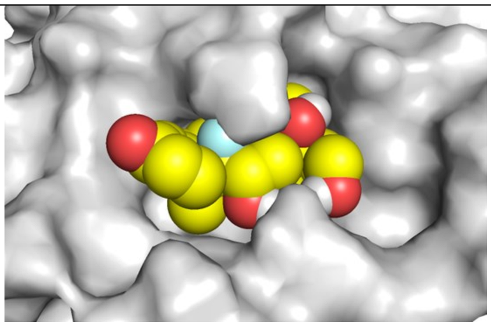	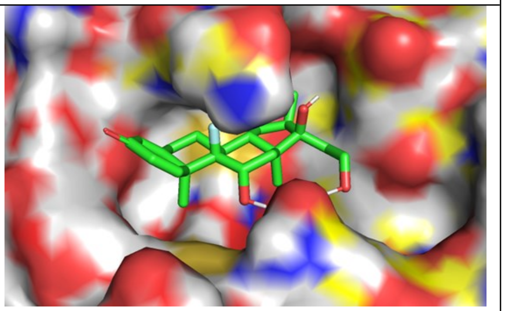
3	Fludrocortisone	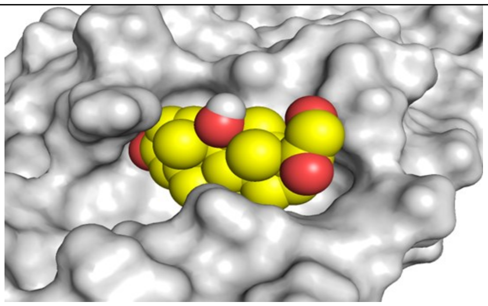	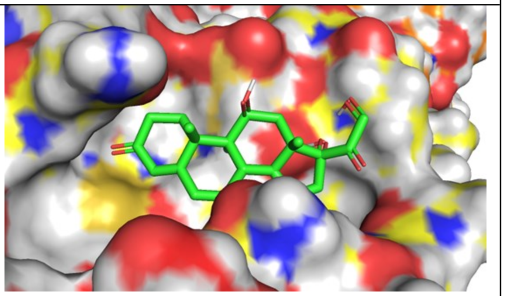
4	Hydrocortisone	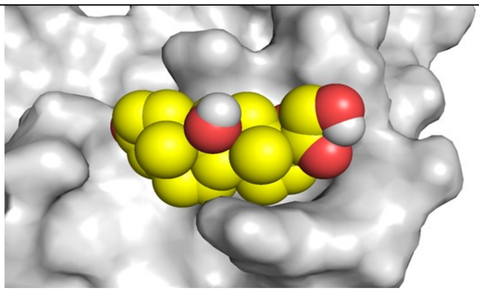	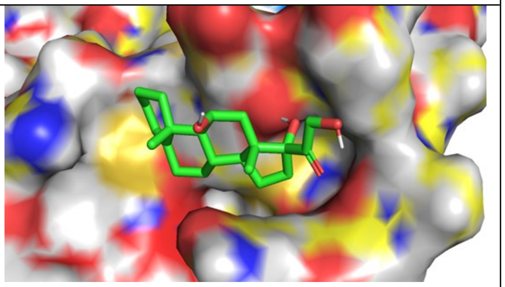

**5**	**Methylprednisolone**	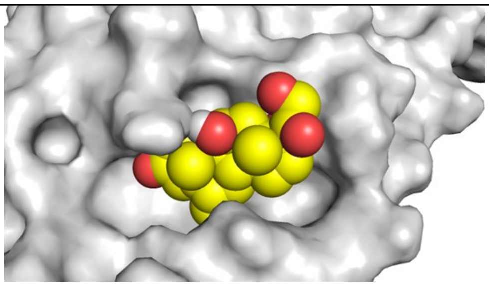	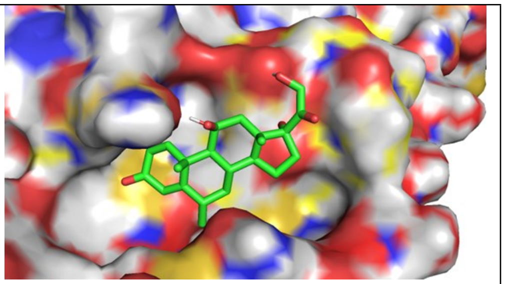
**6**	**Prednisone**	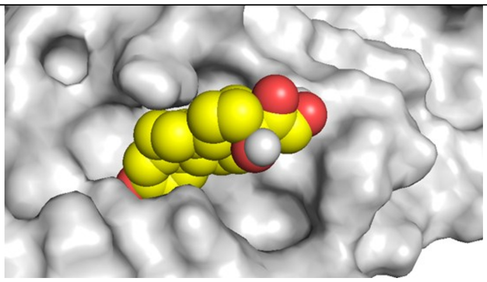	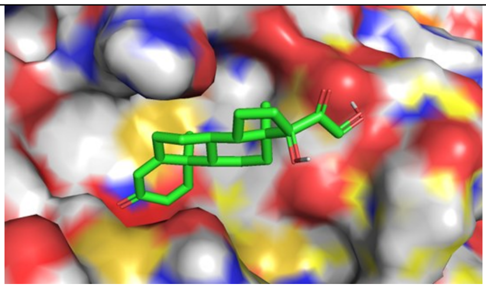
**7**	**prednisolone**	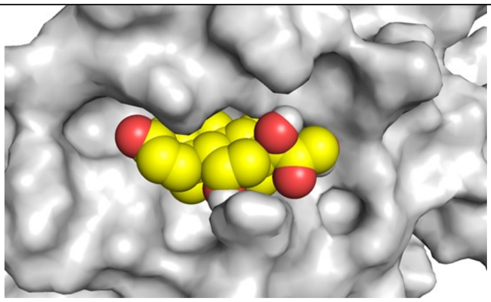	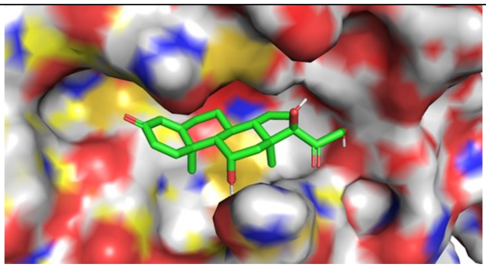
**8**	**Triamcinolone**	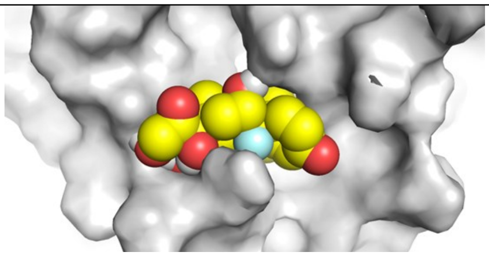	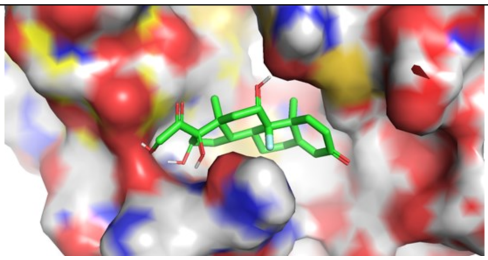
**9**	**Beclomethasone dipropionate**	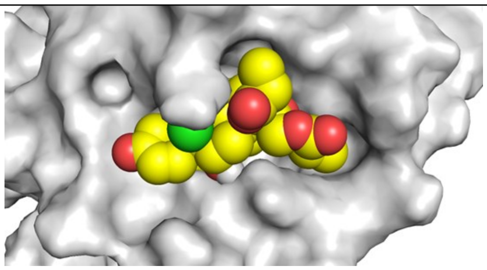	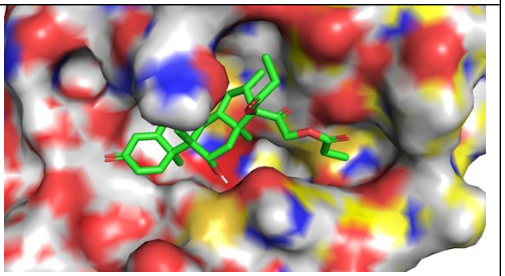

**10**	**Budesonide**	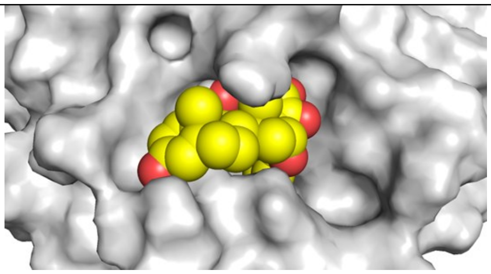	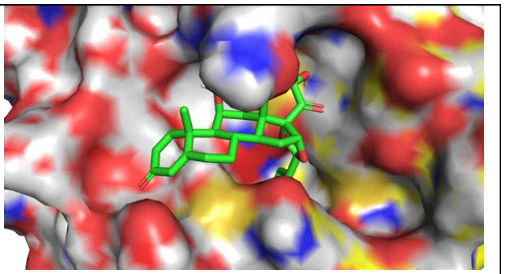
**11**	**Ciclesonide**	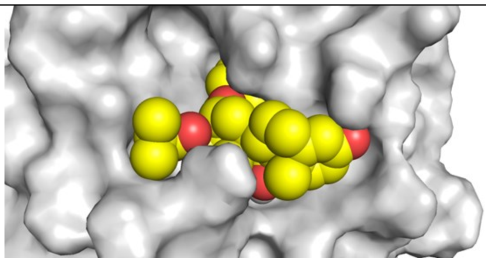	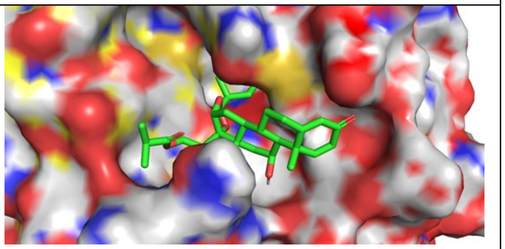
**12**	**Clobetasol propionate**	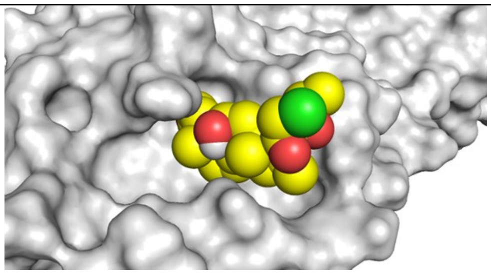	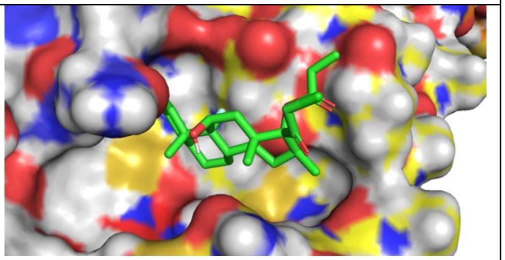
**13**	**Flunisolide**	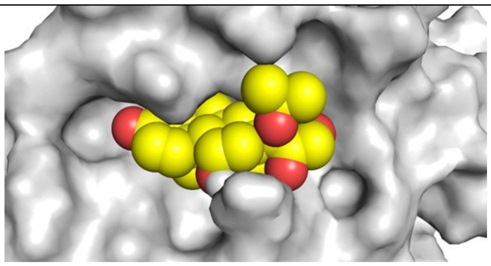	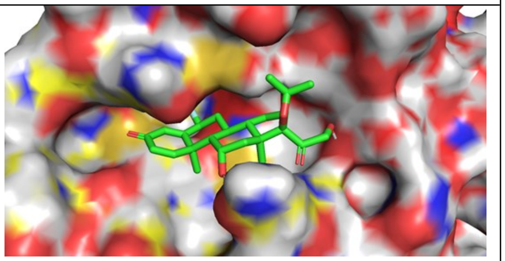
**14**	**Fluocinolone acetonide**	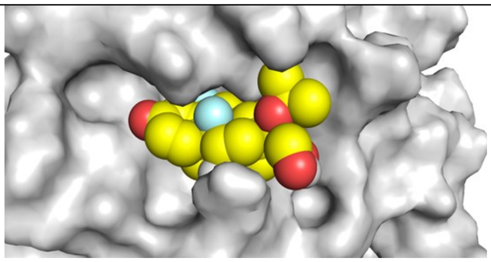	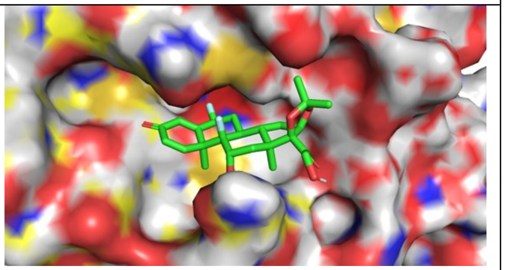

**15**	**Fluocinonide**	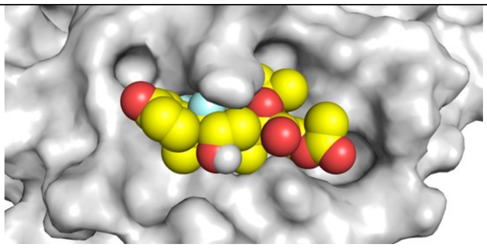	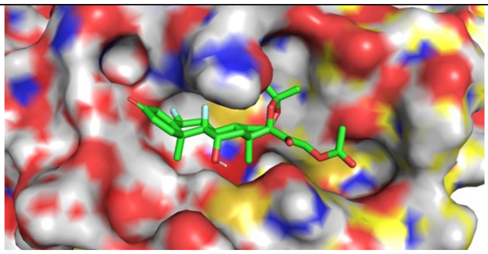
**16**	**Fluorometholone**	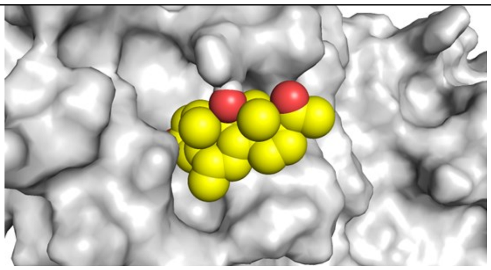	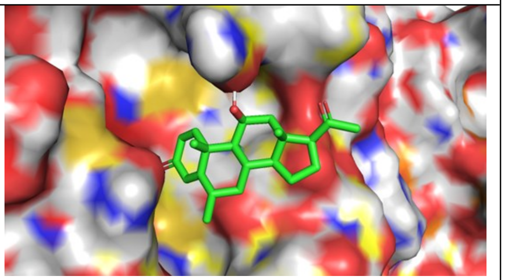
**17**	**Flurandrenolide**	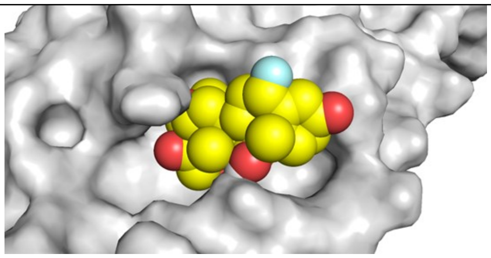	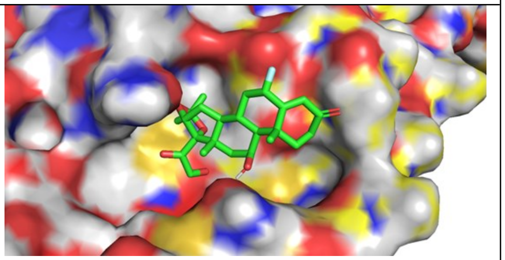
**18**	**Fluticasone propionate**	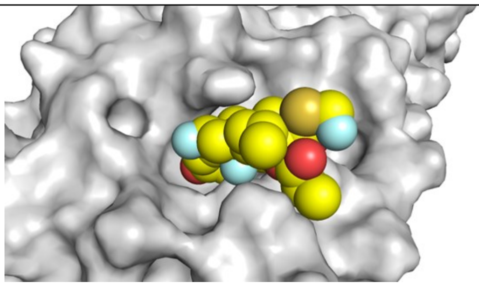	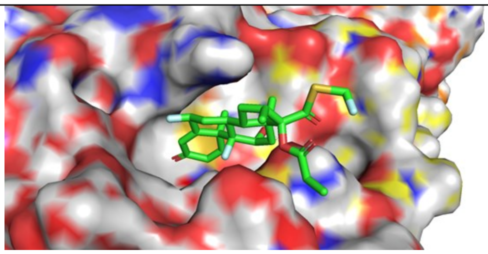
**19**	**Halcinonide**	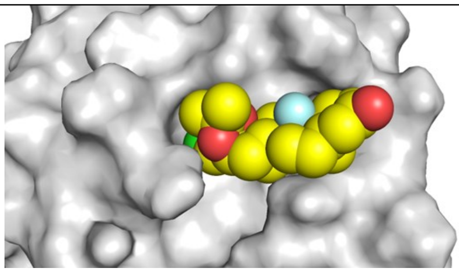	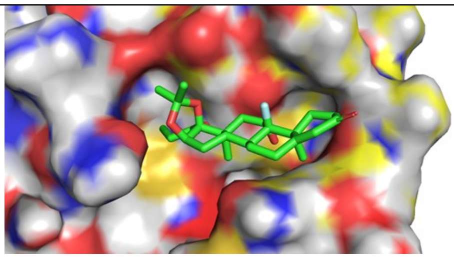

**20**	**Halobetasol propionate**	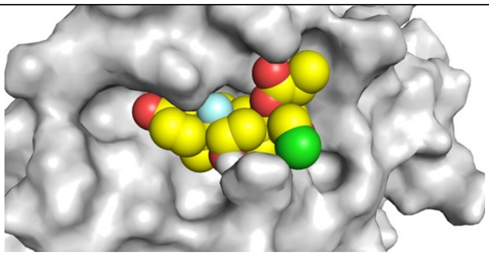	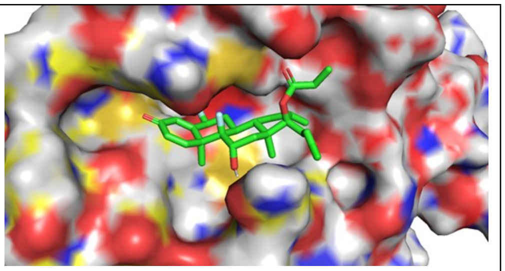
**21**	**Mometasone furoate**	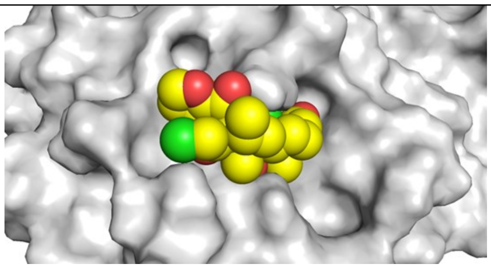	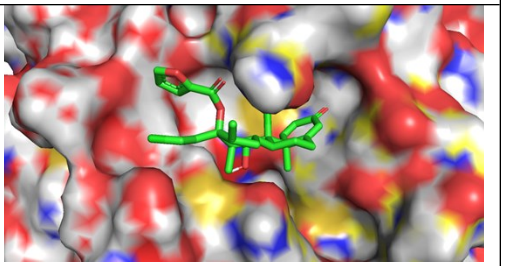
**22**	**Triamcinolone acetonide**	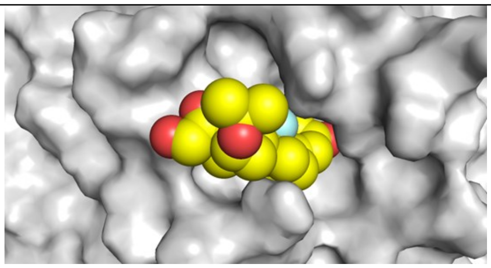	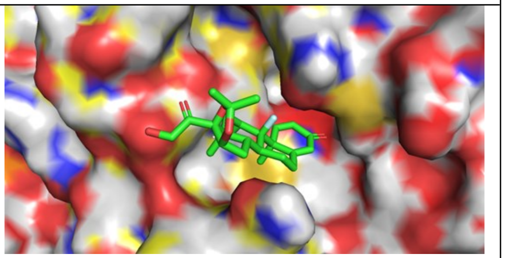
**23**	**N3**	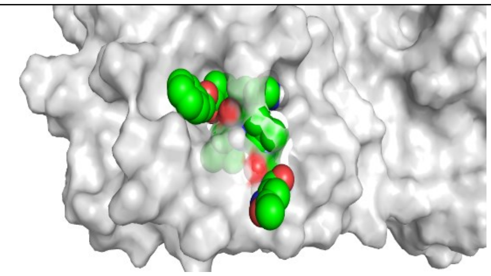	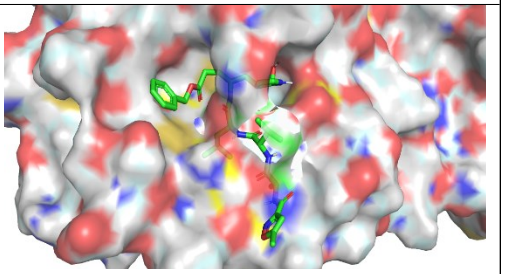

Figure 26: 2 D diagram (a) and 3 D representation (b) of the superimposition of the co-crystallized (red) and the docking pose (green) of N3 in the Covid-19 main protease binding site with RMSD of 1.23 Å.

(a) **(b)**

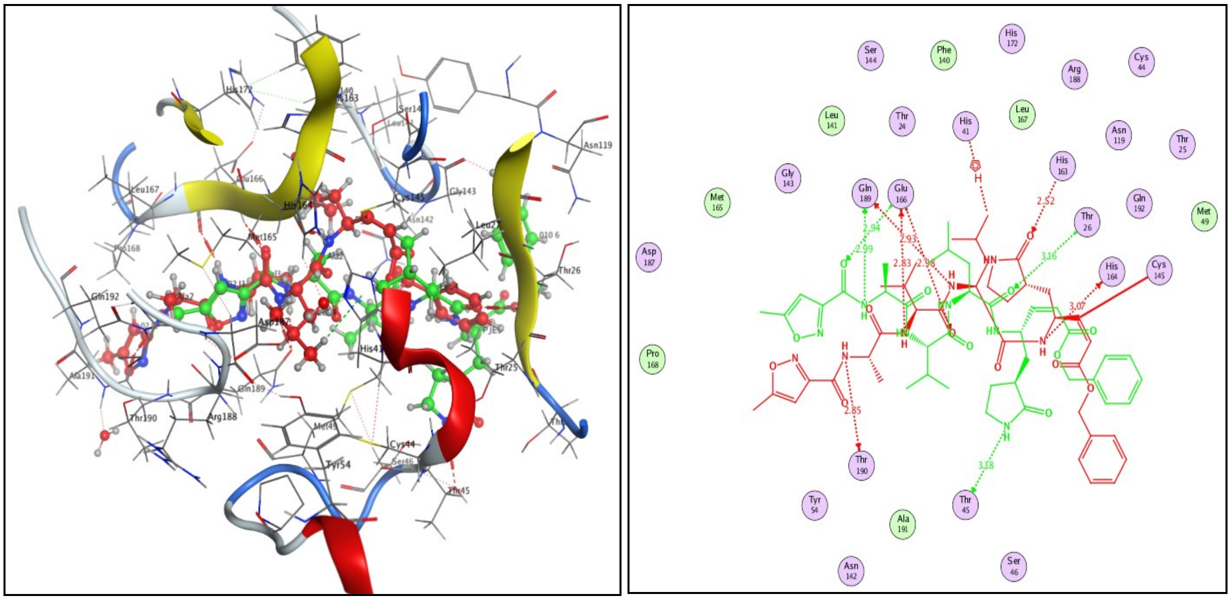
Ser 144
Phe 140
His 172
Arg 188
Cys 44
Leu 141
Thr 24
His 41
Leu 167
Asn 119
Thr 25
Gly 143
His 163
Met 165
Gln 189
Glu 166
Gln 192
Met 49
Thr 26
Asp 187
His 164
Cys 145
Pro 168
Thr 190
Tyr 54
Thr 45
Ala 191
Asn 142
Ser 46
